# The epidemiological and clinical differences between patients with mpox and patients without mpox among suspected cases during the recent global mpox outbreaks: a systematic review and meta-analysis

**DOI:** 10.3389/fcimb.2025.1740617

**Published:** 2026-01-12

**Authors:** Yingying Han, Xingzhao Li, Xin Wang, Zhuan Zhong

**Affiliations:** 1 Department of Neurology, China-Japan Union Hospital of Jilin University, Changchun, Jilin, China; 2 Department of Ultrasound, China-Japan Union Hospital of Jilin University, Changchun, Jilin, China; 3 Hospital, China-Japan Union Hospital of Jilin University, Changchun, Jilin, China; 4 Department of Orthopaedics, The Second Hospital of Jilin University, Changchun, Jilin, China

**Keywords:** clinical characteristic, epidemiological characteristic, meta-analysis, mpox, systematic review

## Abstract

**Background:**

The recent mpox outbreaks have brought challenges to the global health system. We aimed to study the epidemiological and clinical differences between patients with mpox and patients without mpox among suspected cases during and after 2022.

**Methods:**

This study was conducted in accordance with the Preferred Reporting Items for Systematic Reviews and Meta-Analyses (PRISMA) guidelines.

**Results:**

Our meta-analysis included a total of 22 articles regarding 12,850 suspected cases of mpox. The prevalence of mpox was 54%. We found that the proportion of men among the mpox patients was much greater than that among the non-mpox patients (odds ratio (OR)=9.94, 95% confidence interval (CI): 4.37–22.58), and the same trend was also observed (OR = 11.52, 95% CI: 6.22–21.33) for men who have sex with men (MSM). Sexual intercourse (OR = 4.02, 95% CI: 2.63–6.13), multiple sexual partners (OR = 3.36, 95% CI: 1.99–5.68), and anal sex (OR = 2.40, 95% CI: 1.53–3.77) were more common among mpox patients than non-mpox patients. Sexual contact was more strongly associated with mpox infection (OR = 4.39, 95% CI: 1.77–10.92), whereas nonsexual contact (OR = 0.72, 95% CI: 0.63–0.83) and transmission through health services (OR = 0.55, 95% CI: 0.33–0.93) showed weaker associations. Furthermore, there were significant differences between the two patient groups in terms of sexually transmitted infections, symptoms, complications, lesion locations and types of lesions.

**Conclusions:**

Our findings provide a reference for clinicians in the diagnosis and differentiation of mpox. By scientifically understanding these epidemiological and clinical differences, healthcare workers can not only address this epidemic more effectively but also be more fully prepared for the inevitable challenges of future emerging infectious diseases.

**Systematic Review Registration:**

https://www.crd.york.ac.uk/prospero/, identifier CRD420251141869.

## Introduction

Mpox is a re-emerging zoonotic viral disease caused by the mpox virus (Farahat et al., 2022). Mpox virus is a double-stranded DNA virus of the genus Orthopoxvirus of the family Poxviridae that encodes approximately 190 kinds of proteins. These proteins play important roles in DNA replication, transcription, and virion assembly and contribute to promoting activation of and escape from the host immune system (Srivastava et al., 2023). This virus was first reported in the Democratic Republic of the Congo in 1970 (Bunge et al., 2022). The USA reported the first human outbreak outside Africa in July 2003, with 71 nonfatal cases related to 800 specimens from six genera of African rodents imported from Ghana (Centers for Disease Control and Prevention (CDC), 2003). On May 6, 2022, a British national who returned from Nigeria was diagnosed with mpox after a fever rash, marking the beginning of the 2022 epidemic (Girometti et al., 2022). On July 23, 2022, following an accumulation of 3,040 cases in 47 countries, the World Health Organization (WHO) declared mpox as a Public Health Emergency of International Concern, which was lifted on May 11, 2023 (Taylor, 2022; World Health Organization, 2025). There are two distinct clades of the virus. The symptoms caused by Clade I from Central Africa are more severe, with a mortality rate often exceeding 10%. Its ability to spread among humans is relatively limited, and it is mostly transmitted from animals or through close contact. Clade II causes a milder condition, with a mortality rate typically less than 1%, and it is further divided into clades IIa and IIb. Clade IIa is prevalent mainly in West Africa. Clade IIb was the main strain that caused global outbreaks in 2022 and beyond, with significantly higher infectivity than IIa and demonstrating efficient interpersonal transmission ability, usually through close or intimate contact, including sexual networks, especially in nonendemic regions (Antunes et al., 2022; Bunge et al., 2022). The incubation period of mpox is 7 to 14 days, and the longest is 21 days, during which there is no clinical manifestation of viral disease (Hussain et al., 2022). The incubation period is followed by the prodromal period, when individuals may have various symptoms, including lymphadenopathy, fever, headache, myalgia and fatigue (Huhn et al., 2005). Mpox is a systemic disease that presents in most patients with skin lesions, and the vesiculopustules are firm, difficult to deroof, and lack fluid content after deroofing (Khanna et al., 2023).

The definition of epidemiological cases, especially at the beginning of an epidemic, always has high sensitivity and low specificity (El-Gilany, 2021; Nunez et al., 2022). In practice, there may also be differences in the timeliness and consistency of adoption and application in different countries, regions, and even medical institutions. Some healthcare workers lack appropriate training and experience in diagnosing mpox cases, especially in nonendemic areas (Tarín-Vicente et al., 2022; Thornhill et al., 2022). Although cutaneous manifestations are signs of mpox, other diseases, such as syphilis, rickettsial pox, scabies, molluscum contagiosum and varicella zoster, present with similar rashes (Cowen et al., 2023; Hussain et al., 2022). Polymerase chain reaction (PCR) is the gold standard for mpox diagnosis, but in less developed countries or regions, PCR testing cannot achieve complete coverage. For instance, in the Democratic Republic of the Congo, only the national reference laboratory in Kinshasa, the capital, can perform PCR testing, while endemic areas are often located in remote areas (Mande et al., 2022). The purpose of this study was to compare and analyze the clinical and epidemiological characteristics of patients with mpox and patients without mpox among suspected cases, which may help differentiate mpox infection from alternative diagnoses. During and after the 2022 outbreak, the clades of the virus, their transmission routes, outbreak area, lesion site and disease severity were quite different from those of the previous mpox epidemic (Antunes et al., 2022; Ogoina et al., 2020; Thornhill et al., 2022; Yinka-Ogunleye et al., 2019). For the sake of rigorous results, we did not include data prior to 2022.

## Materials and methods

This study was conducted in accordance with the Preferred Reporting Items for Systematic Reviews and Meta-Analyses (PRISMA) statement (PROSPERO registration number: CRD420251141869).

### Eligibility criteria

We included case–control, cohort, and cross-sectional studies. If the number of suspected cases and the number of confirmed cases of mpox were available in a case series, they were also included in our analysis to calculate the prevalence. Other inclusion criteria were as follows: 1) all patients were suspected mpox patients; 2) the epidemiological or clinical characteristics of the mpox and non-mpox patients were described in the article; and 3) the samples were collected from patients during and after the 2022 outbreak of mpox.

The exclusion criteria were as follows: 1) nonhuman studies; 2) data duplication; 3) case reports, reviews or comments; 4) data from before 2022; 5) suspected cases without PCR results for mpox; and 6) a sample size of the experimental group or the control group less than five.

### Information sources

We searched for articles published in PubMed, Embase and Web of Science before July 24, 2025. To retrieve the available data to the maximum extent possible, we did not limit the language of the articles, and the retrieval scope included the title and abstract. In addition, we conducted an initial reference review, which checked the reference lists of the included articles, but no additional articles were found to be suitable for inclusion.

### Search strategy

The search strategy was as follows: ((((monkeypox[Title]) OR (mpox[Title])) OR (mpxv[Title])) OR (mpx[Title])) AND ((((((((suspects[Title/Abstract]) OR (suspected[Title/Abstract])) OR (suspicion[Title/Abstract])) OR (mpox-like[Title/Abstract])) OR (possible[Title/Abstract])) OR (probable[Title/Abstract])) OR (negative[Title/Abstract])) OR (PCR-negative[Title/Abstract])).

### Study selection process

We first deleted duplicate articles by matching titles, authors and journals. We subsequently performed a preliminary screening of the articles by reading the title or abstract. The articles that passed the preliminary screening were further screened by reading the full text to determine which articles were eligible for meta-analysis.

### Data selection process and items

Data extraction was performed by three authors to ensure accuracy. Two of the authors independently screened the data, and disagreements were adjudicated by the third author.

The selected data included sex, age, race, men who have sex with men (MSM) status, exposure, transmission route, presence of sexually transmitted infections (STIs), use of pre-exposure prophylaxis (PrEP), lesion location, lesion type, complications, symptoms, and mpox vaccination status.

### Assessment of study risk of bias

The Newcastle–Ottawa Quality Assessment Scale was used to evaluate the bias risks of the comparative observational studies, and an article with a score≥7 indicated a low risk of bias. The Joanna Briggs Institute Critical Appraisal Checklist was used to assess the risk of bias in case series, and an article that met seven or more criteria was considered to have a low risk of bias.

### Assessment of reporting bias

Because the number of studies included in some of the analyses was less than 10, which was not suitable for funnel plots, we employed Egger’s test to evaluate reporting bias, with a p value>0.05 indicating an absence of bias.

### Statistical analysis

Odds ratios (ORs) and standardized mean differences (SMDs) were used for data analysis and evaluation; ORs were used for dichotomous variables, SMDs were used for continuous variables, and confidence intervals (CIs) were set at 95%. For data with only the sample size and quartile given, we used a transformation formula to calculate the mean and standard deviation (Wan et al., 2014). Heterogeneity was quantified by I^2^ statistics, and the source of heterogeneity was determined via subgroup analysis: I^2^ ≤ 50% indicated low heterogeneity, 50%<I^2^ ≤ 75% indicated moderate heterogeneity, and I^2^>75% indicated high heterogeneity (Higgins et al., 2003). We refer to the studies that do not provide a clear definition of suspected cases or whose definitions are ambiguous or significantly different from those used in other studies as “studies with insufficient definitional information”. After these studies were excluded, a sensitivity analysis was conducted to verify the robustness of the results. A random effects model was used to estimate the effect value. To handle variables with zero cells in the 2-by-2 tables, we applied a continuity correction by adding 0.5 to all four cells of those variables before pooling. The statistical software used was Stata 14.0, and a z test p value<0.05 was considered to indicate statistical significance.

## Results

### Study selection

**Figure 1:**
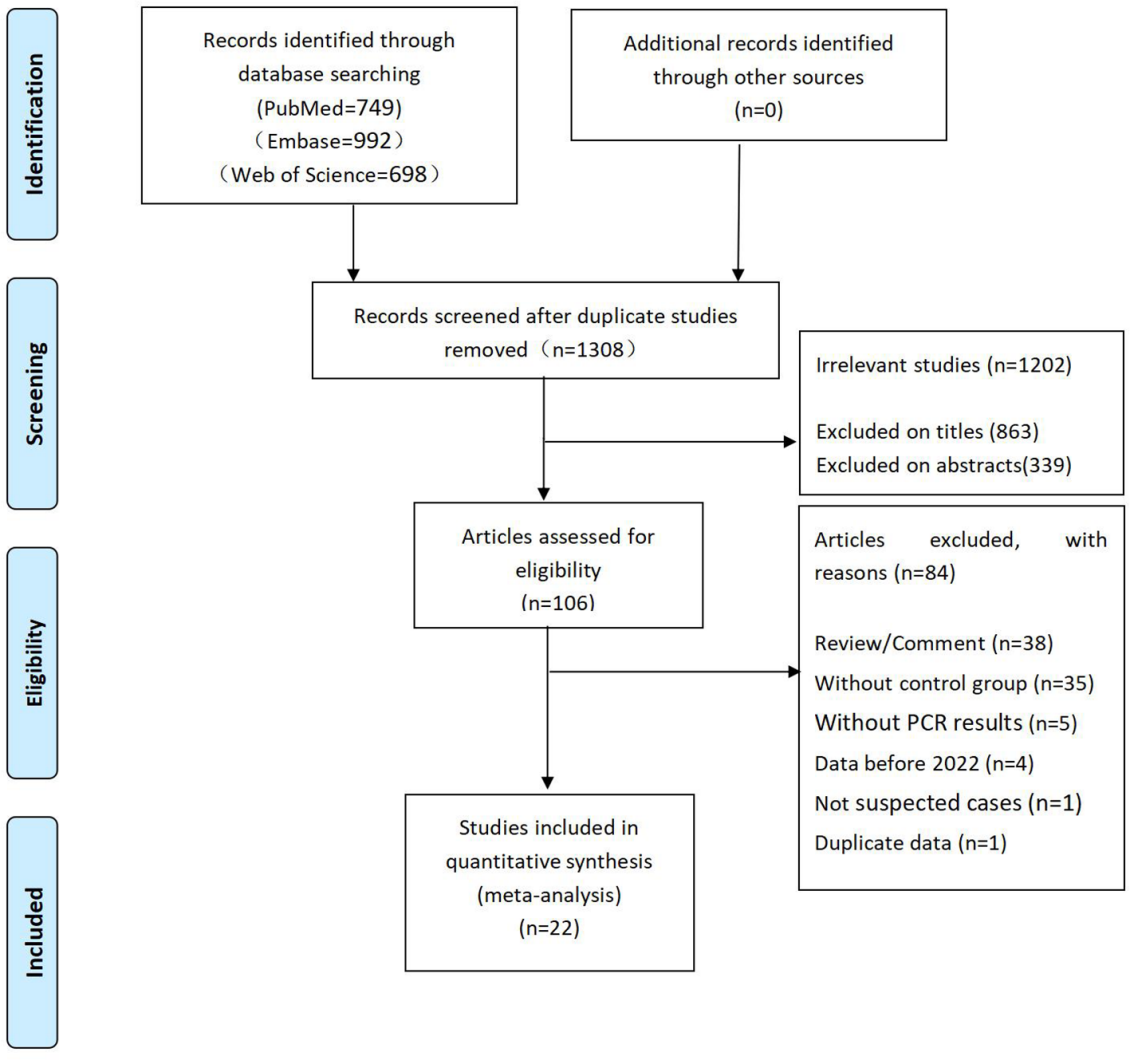
Flow diagram of the article selection process.

We retrieved a total of 2,439 articles from PubMed, Embase and Web of Science and excluded 1131 duplicate articles. A total of 1,202 articles that were found not to be relevant to our study following title and abstract screening were excluded. Of the remaining 106 articles, 84 were further excluded after the full text was screened. A flow diagram of the article selection process is shown in **Figure 1**.

### Risk of bias in studies

The Newcastle–Ottawa Quality Assessment Scale and the Joanna Briggs Institute Critical Appraisal Checklist are described in **Supplementary Tables S1** and **S2**, respectively. We found that most of the articles included had a low risk of bias.

### Characteristics of individual studies

Our meta-analysis included a total of 22 articles, with sample data all from 2022 and later. The data were from 16 countries and included 12,850 suspected mpox cases, each of which was subjected to PCR testing. The definitions of suspected mpox cases are detailed in **Table 1**.

**Table 1:** Characteristics of individual studies.

Study	Study period	Country	Continent	Mpox cases	Non-mpox cases	Definition of suspected cases
Campoy et al., 2024	2022.5.2- 2023.2.2	Spain	Europe	54	32	Patients meeting the WHO’s case definition of probable or suspected mpox issued as of August 25, 2022 (WHO. 2022a).
Silva et al., 2022	2022.6.12-2022.8.19	Brazil	South America	208	134	The samples are all suspected mpox patients, but the inclusion criteria of suspected cases were not explained in the article.
Arboleda et al., 2023	2022.8- 2022.12	Colombia	South America	102	60	The samples are all suspected mpox patients, but the inclusion criteria of suspected cases were not explained in the article.
Hens et al., 2023	2022.5.23-2022.9.20	Belgium	Europe	155	51	Patients meeting the WHO’s case definition of probable or suspected mpox issued as of August 25, 2022 (WHO. 2022a).
Heukelom et al., 2023	2022.5.20-2022.9.15	Netherlands	Europe	135	239	The MSM who present with malaise, and/or ulcerative lesions, and/or proctitis and/or a papular-vesicular-pustular eruption.
Costa et al., 2023	2022.6.18-2022.9.22	Brazil	South America	309	85	Suspected case is defined a person with sudden onset of an acute skin rash suggestive of mpox (deep and well-circumscribed lesions, often with central umbilication; and progression of the lesion through specific sequential stages – macules, papules, vesicles, pustules and crusts) single or multiple on any part of the body (including genital region), associated or not with adenomegaly or report of fever AND having one of the epidemiological links: report of intimate contact with casual partner(s), in the last 21 days prior to the onset of signs and symptoms or contact with a suspected, probable or confirmed case of mpox prior to the onset of symptoms or travel to an endemic country or country with confirmed cases of mpox in the 21 days prior to the onset of signs and symptoms.
Hohan et al., 2024	2022.6- 2023.5	Romania	Europe	18	29	The subjects with suggestive cutaneous or mucosal lesions for suspected mpox.
Purnama et al., 2025	2023.10- 2024.2	Indonesia	Asia	58	155	A suspected case is defined as a person with a recent onset of rash lesions on the skin or mucous membranes, possibly accompanied by symptoms such as fever, chills, myalgias, headaches, lymphadenopathy, low back pain, asthenia, proctitis, or a history of contact with a confirmed case within the last 21 days, alongside travel history to endemic areas.
Estevez et al., 2023	2022.5.18-2022.9.30	Spain	Europe	100	33	A suspected mpox case is defined by an individual clinically presenting with a localized or generalized rash (with vesicular or pustular lesions, especially if they are umbilicated) and one or more of the following signs/symptoms: fever (>38.5 °C), severe headache, myalgia, arthralgia, backache or enlarged lymph nodes.
Acevedo et al., 2023	2022.6.1- 2022.9.30	Chile	South America	961	454	Person with a recent onset rash lesion on the skin or mucous membranes (single or simple) and that may present alone or associated with one or more of the following symptoms: fever greater than 38.5 °C, chills, myalgias, headaches, lymphadenopathy, low back pain, asthenia, proctitis. Or who also has a history of contact with a confirmed case during the last 21 days.
Brosius et al., 2025	2024.5.2- 2024.10.9	Democratic Republic of the Congo	Africa	407	103	The samples are all suspected mpox patients, but the inclusion criteria of suspected cases were not explained in the article.
Shim et al., 2024	2022.6.21-2023.10.31	Korea	Asia	95	65	A suspected patient is defined as someone who exhibits clinical symptoms and an epidemiological link suggesting mpox infection. This category also includes individuals without a confirmed epidemiological link to mpox, such as overseas travel or sexual contact history, but who display clinical symptoms typical of mpox, including skin rashes on the anorectum, genitalia, oral cavity, conjunctiva, or urethra, as well as anal or genital pain. Additionally, it encompasses individuals with a strong epidemiological link to mpox, such as sexual contact with a symptomatic person, but who only exhibit nonspecific symptoms like chills, myalgia, sore throat, fever, sweating, fatigue, headache, body aches, back pain, and lymphadenopathy. Clinical manifestations include an acute rash or pain of unknown origin on the skin or mucous membranes, accompanied by 1 or more of the following symptoms: acute fever (temperature≥38.5 °C), headache, lymphadenopathy (inflammation or enlargement), back pain, myalgia, or dysuria. Epidemiological linkage is assessed based on criteria such as contact with a patient with confirmed or suspected mpox within 21 days prior to symptom onset, travel to a country with an mpox epidemic or an area where cases have been reported since May 2022, or sexual contact with multiple or casual partners.
Lucero-Obusan et al., 2024	2022.5.23-2023.5.24	USA	North America	251	802	The person who has suspected lesions.
Doncell et al., 2022	2022.6.9- 2022.9.15	Argentina	South America	56	26	Situation 1: The patient experiences rash without a definite etiology and proctitis without a definite etiology, and at least one of the following epidemiological events has occurred within 21 days before symptoms appear: -Direct physical contact, including sexual contact, with a suspected or confirmed patient. - Contact with contaminated materials (clothing) of a suspected or confirmed patient. - Close contact with a suspected or confirmed patient without respiratory protection - Having sex with new, multiple or occasional sexual partners. Situation 2: Any person who has been in direct contact with (suspected or confirmed) a mpox patient and has one or more of the following signs or symptoms within 5 to 21 days of exposure risk: sudden onset of fever>38.5 °C, lymphadenopathy, asthenia, headache, myalgia and lumbar pain. Situation 3: Any person who does not present or report a clear epidemiological history but presents a characteristic rash and high clinical suspicion.
Martinez-Arias et al., 2025	2022.6.1- 2023.12.31	Spain	Europe	16	44	Epidemiological criteria: (If during the 21 days before the onset of symptoms one or more of the following background are identified) -A close contact with a confirmed or probable case of mpox. -Antecedents of relationships in high-risk sexual contexts. -History of travel to endemic areas of West/Central Africa where the circulation of the virus has been identified. Clinical criteria: -Presence of vesicular or pustular rash (especially if it is umbilical) in any part of the body. -And, in addition, presence of one or more of the following signs/symptoms: fever (38.5 °C), intense headache, myalgia, arthralgia, back pain and lymphadenopathy.
Ogoina et al., 2024	2022.6.1- 2022.12.30	Nigeria	Africa	137	55	We define a suspected case of mpox by using the Nigeria Centre for Disease Control and Prevention guidelines, as previously described (Nigeria Centre for Disease Control, 2019).
Nunez et al., 2023	2022.5.24-2022.11.21	Mexico	North America	3291	1787	A suspected mpox case (a person with mpox-like illness) is someone with one or more acute skin lesions (of any kind) and at least one of the following symptoms: fever, myalgia, headache, adenopathy, fatigue, arthralgia, and back pain with no other alternative condition explaining the symptoms.
Kengea et al., 2025	2024.8.1- 2024.11.10	Democratic Republic of the Congo	Africa	51	146	Suspected case: any person who has been exposed to a case of mpox and who has acute fever (≥38°C) or who has acute fever and symptoms of mpox, or any person who has one or more maculopapular rashes or scabs.
Rimmer et al., 2023	2022.5.9- 2022.6.29	United Kingdom	Europe	70	70	The patients who meet the UK Health Security Agency case definition for suspected mpox (UK Health Security Agency, 2022).
Moretti et al., 2023	2022.5.28-2022.7.22	Belgium	Europe	85	56	Mpox suspicion is defined in the current study as a patient presenting with general symptoms (at least one of the following: fever, fatigue, headache, back pain, myalgia, and perspiration), and skin or mucosal eruption (vesicular pustular eruption with at least the presence of a scab, or ulceration, or crusted lesion).
Mortier et al., 2023	2022.5.15-2022.11.15	France	Europe	143	229	The person who has clinical signs consistent with mpox according to the treating physician.
Junior et al., 2023	2022.7.12-2023.3.27	Brazil	South America	362	1131	Suspected cases of mpox are considered to be: individuals of any age, with sudden onset of acute rash, single or multiple, on any part of the body, associated or not enlarged lymph nodes or fever, and one of the following: (a) history of intimate contact with stranger(s) and/or casual partner(s) in the last 21 days before the onset of signs/symptoms; (b) epidemiological link with a suspected or confirmed case of mpox before 21 days after the onset of signs/symptoms; (c) epidemiological link with people with a history of travel to an endemic country or with confirmed cases of mpox up to 21 days before the onset of signs/symptoms.

MSM, men who have sex with men; USA, The United States of America; WHO, World Health Organization.

The samples from Nunez et al. (2023) and Herran-Arita et al. (2023) were from the Mexican Ministry of Health, and the study periods were almost the same. Because Nunez et al. included deidentified data on all Mexican individuals with mpox-like illness who were tested from May 24 to November 21, 2022, and the sample size was much larger than that of the study of Herran-Arita et al., we did not include data from Herran-Arita et al. in the meta-analysis to avoid data duplication.

### Results of syntheses

**Figure 2:**
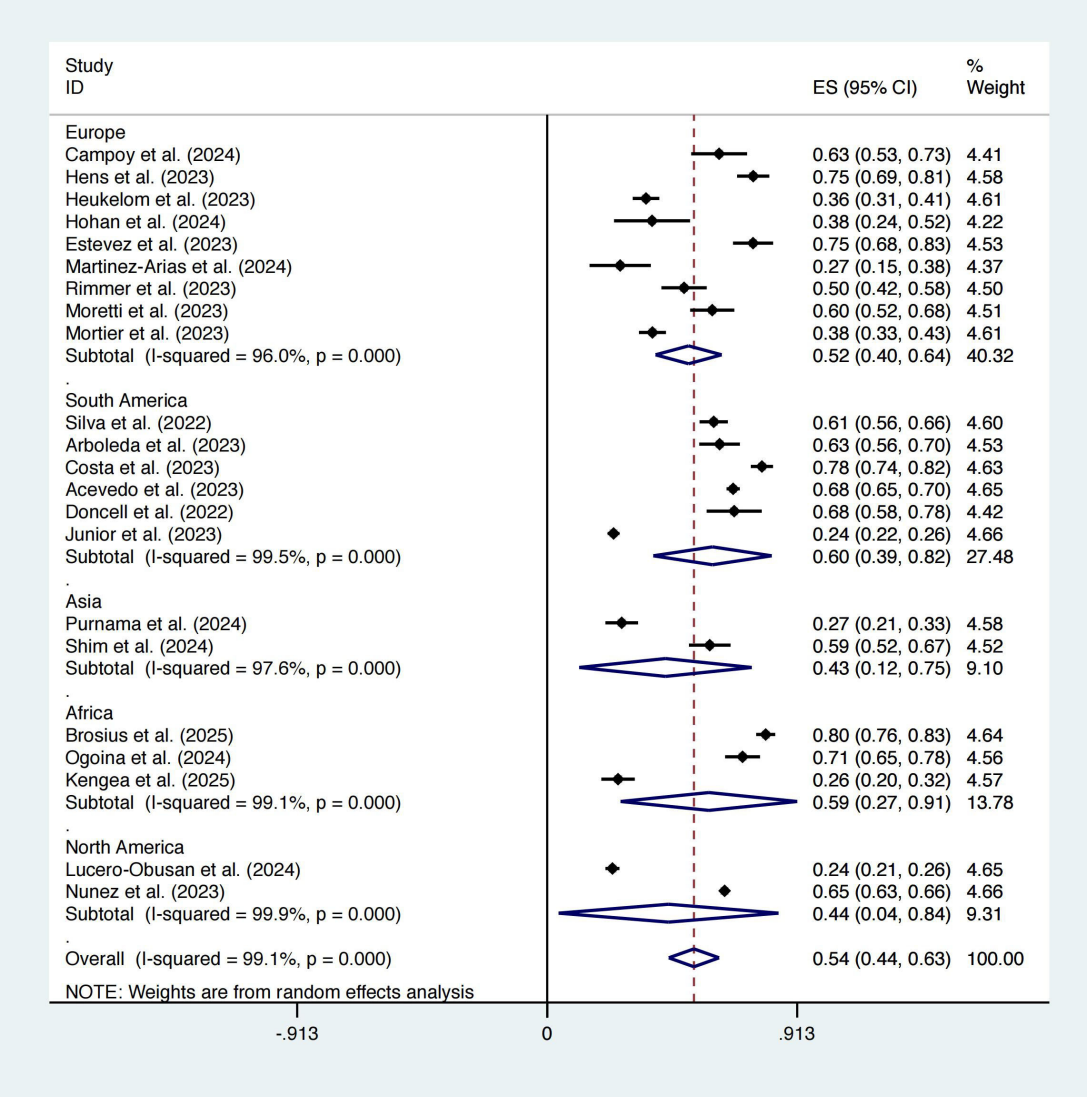
Forest plot of mpox prevalence among suspected cases.

In the study by Rimmer et al (Rimmer et al., 2023), the sample size of the control group was matched according to the experimental group, so we did not include its data in the calculation of prevalence. In our findings, the prevalence of mpox was 54% (95% CI: 44%–63%; **Figure 2**) among suspected cases. Subgroup analysis revealed that South America had the highest prevalence (60%, 95% CI: 39%–82%) and that Asia had the lowest prevalence (44%, 95% CI: 4%–84%).

The epidemiological and clinical differences between mpox patients and non-mpox patients are listed in **Table 2** and **Supplementary Figures S1**-**S54**. The proportion of men among the mpox patients was much greater than that among the non-mpox patients (OR = 9.94, 95% CI: 4.37–22.58), and the same was also observed among MSM (OR = 11.52, 95% CI: 6.22–21.33). Age (SMD = 0.12, 95% CI: -0.05–0.28), race (Black (OR = 1.52, 95% CI: 1.00–2.33); White (OR = 0.77, 95% CI: 0.51–1.17); Asian (OR = 0.26, 95% CI: 0.04–1.69)) and previous mpox vaccination status (OR = 0.54, 95% CI: 0.26–1.11) were not significantly correlated with mpox virus infection.

**Table 2:** Epidemiological and clinical characteristics of mpox and non-mpox patients.

Items		Number of included articles	Number of included articles ^*^	OR	OR^*^	SMD	SMD^*^	95% CI	95% CI^*^	I^2^	I^2*^	p	p^*^
Age		17	11			0.12	0.12	-0.05–0.28	-0.04–0.29	92.3%	87.5%	0.170	0.152
Sex (men)		20	15	9.94	10.65			4.37–22.58	4.01–28.28	93.0%	92.1%	<0.001	<0.001
MSM		8	6	11.52	13.50			6.22–21.33	5.98–30.48	85.0%	85.2%	<0.001	<0.001
Race													
	Black	4	2	1.52	1.34			1.00–2.33	0.53–3.36	74.5%	87.5%	0.052	0.537
	White	4	2	0.77	1.00			0.51–1.17	0.76–1.32	74.7%	0.0%	0.224	0.985
	Asian	2	1	0.26	NA			0.04–1.69	NA	0.8%	NA	0.157	NA
Mpox vaccination		6	4	0.54	0.85			0.26–1.11	0.53–1.39	82.5%	0.0%	0.093	0.525
Recent exposure													
	Contact with previous mpox patients	4	4	2.53	NA			1.56–4.12	NA	40.0%	NA	<0.001	NA
	Sexual intercourse	7	4	4.02	4.00			2.63–6.13	1.94–8.24	53.6%	63.4%	<0.001	<0.001
	Anal sex	2	1	2.40	NA			1.53–3.77	NA	0.0%	NA	<0.001	NA
	Oral sex	3	2	1.29	1.50			0.50–3.34	0.31–7.29	78.1%	88.5%	0.606	0.614
	Multiple sexual partners	7	6	3.36	3.78			1.99–5.68	2.18–6.53	78.8%	77.6%	<0.001	<0.001
	Travel	9	6	1.20	1.37			0.91–1.59	1.04–1.80	32.9%	0.0%	0.204	0.026
Transmission routes													
	Health services	3	3	0.55	NA			0.33–0.93	NA	0.0%	NA	0.025	NA
	Sexual contact	4	3	4.39	7.82			1.77–10.92	5.78–10.59	85.6%	9.8%	0.001	<0.001
	Nonsexual contact	2	2	0.72	NA			0.63–0.83	NA	0.0%	NA	<0.001	NA
STIs													
	HIV	18	13	3.60	3.37			2.18–5.97	1.75–6.51	94.9%	95.9%	<0.001	<0.001
	Syphilis	7	3	1.89	2.40			1.07–3.35	1.01–5.69	77.0%	74.4%	0.028	0.047
	Gonorrhea	6	2	2.20	1.71			1.30–3.72	0.71–4.12	52.9%	0.0%	0.003	0.233
	Hepatitis B virus	2	1	1.84	NA			0.45–7.47	NA	0.0%	NA	0.392	NA
	Hepatitis C virus	4	3	2.96	2.18			0.95–9.18	0.56–8.49	56.5%	65.2%	0.061	0.260
	Chlamydia	5	3	1.66	1.77			0.94–2.94	0.45–6.98	46.0%	0.0%	0.079	0.418
PrEP		6	3	2.03	2.37			1.24–3.33	1.17–4.79	66.4%	51.3%	<0.001	0.017
Locations of lesions													
	Palms	3	3	0.83	NA			0.73–0.94	NA	0.0%	NA	0.003	NA
	Soles	4	4	0.62	NA			0.54–0.71	NA	0.0%	NA	<0.001	NA
	Neck	2	2	0.78	NA			0.69–0.89	NA	0.0%	NA	<0.001	NA
	Face	7	7	0.99	NA			0.71–1.38	NA	67.8%	NA	0.965	NA
	Head	4	3	0.90	0.90			0.80–1.02	0.79–1.02	0.0%	0.0%	0.097	0.086
	Arms	5	5	0.94	NA			0.72–1.24	NA	48.7%	NA	0.676	NA
	Legs	5	5	0.86	NA			0.72–1.03	NA	17.2%	NA	0.101	NA
	Trunk	4	3	1.13	1.08			0.74–1.72	0.60–1.96	44.7%	56.1%	0.563	0.792
	Oral cavity	5	5	0.96	NA			0.52–1.74	NA	30.5%	NA	0.882	NA
	Genitals	10	7	2.90	2.85			2.16–3.89	1.96–4.15	55.1%	49.8%	<0.001	<0.001
	Perianal region	6	5	3.20	2.72			1.94–5.28	1.76–4.22	44.2%	25.8%	<0.001	<0.001
Types of lesions													
	Maculae	4	3	0.64	0.65			0.58–0.72	0.58–0.72	0.0%	0.0%	<0.001	<0.001
	Papulae	5	4	0.86	1.02			0.63–1.19	0.90–1.17	59.0%	2.7%	0.367	0.718
	Vesicles	6	5	1.003	0.87			0.76–1.32	0.71–1.07	64.9%	33.0%	0.985	0.189
	Pustules	7	5	2.08	1.68			1.55–2.80	2.24–2.99	69.6%	62.1%	<0.001	<0.001
	Scabs	6	5	1.12	1.22			0.83–1.51	0.92–1.62	61.0%	55.0%	0.472	0.172
Complications													
	Proctitis	6	3	4.13	5.5			1.91–8.95	2.71–11.13	71.8%	0.0%	<0.001	<0.001
	Bacterial infection	3	2	1.43	2.88			0.39–5.27	0.75–11.05	47.8%	0.0%	0.587	0.124
	Urethritis	2	2	2.81	NA			0.33–24.04	NA	52.2%		0.346	NA
Symptoms													
	Systemic symptoms	5	4	2.58	2.58			1.56–4.27	1.28–5.18	61.2%	70.5%	<0.001	0.008
	Cough	3	2	0.85	0.85			0.72–1.00	0.63–1.15	0.0%	1.4%	0.050	0.305
	Fever	14	11	2.38	2.24			1.80–3.15	1.65–3.02	77.8%	77.7%	<0.001	<0.001
	Lymphadenopathy	10	8	3.41	3.34			2.72–4.28	2.59–4.30	44.1%	48.4%	<0.001	<0.001
	Headache	4	1	0.90	NA			0.80–1.02	NA	0.0%	NA	0.097	NA
	Sore throat	7	5	1.18	1.13			0.88–1.59	1.01–1.27	38.6%	0.0%	0.270	0.031
	Asthenia	8	7	1.27	1.19			0.92–1.75	0.84–1.68	56.3%	56.3%	0.143	0.335
	Fatigue	5	3	1.16	1.12			0.60–2.22	0.35–3.58	67.4%	81.0%	0.664	0.855
	Myalgia	12	10	1.72	1.63			1.32–2.23	1.24–2.15	70.3%	71.3%	<0.001	<0.001
	Diarrhea	3	2	2.24	2.42			1.20–4.19	0.76–7.69	28.2%	64.1%	0.011	0.136
	Nausea	2	1	0.92	NA			0.63–1.34	NA	17.7%	NA	0.667	NA
	Arthralgia	6	4	1.07	1.06			0.62–1.86	0.95–1.19	56.2%	0.0%	0.800	0.279

*Results of the sensitivity analysis.

Health services, These services are not limited to the occupational transmission among health workers but also include infections patients may acquire during medical services.

MSM, men who have sex with men; NA, not applicable; OR, odds ratio; PrEP, pre-exposure prophylaxis; SMD, standardized mean differences; STI, sexually transmitted infection.

We compared the recent exposures of the two groups of patients and reported that contact with previous mpox patients was more common among mpox patients (OR = 2.53, 95% CI: 1.56–4.12). Recent sexual intercourse (OR = 4.02, 95% CI: 2.63–6.13) and having multiple sexual partners (OR = 3.36, 95% CI: 1.99–5.68) were more commonly reported among mpox patients compared to non-mpox patients. We further analyzed data related to sexual behavior and found that, although oral sex occurrence was not significantly different between mpox patients and non-mpox patients (OR = 1.29, 95% CI: 0.50–3.34), anal sex was significantly more common among mpox patients (OR = 2.40, 95% CI: 1.53–3.77). We also compared the recent travel circumstances of the two groups, and there was no obvious difference (OR = 1.20, 95% CI: 0.91–1.59).

There was also a significant difference between the transmission routes of patients with mpox and those without mpox. Sexual contact was more strongly associated with mpox infection (OR = 4.39, 95% CI: 1.77–10.92), whereas nonsexual contact (OR = 0.72, 95% CI: 0.63–0.83) and transmission through health services (OR = 0.55, 95% CI: 0.33–0.93) showed weaker associations. Patients with mpox also showed high susceptibility to STIs, among which the number of patients infected with HIV (OR = 3.60, 95% CI: 2.18–5.97), syphilis (OR = 1.89, 95% CI: 1.07–3.35) or gonorrhea (OR = 2.20, 95% CI: 1.30–3.72) was significantly greater than that of patients not infected with mpox; however, there was no significant difference in the findings of infection with hepatitis B virus (OR = 1.84, 95% CI: 0.45–7.47), hepatitis C virus (OR = 2.96, 95% CI: 0.95–9.18) or chlamydia (OR = 1.66, 95% CI: 0.94–2.94) between groups. We also found that among the HIV-negative population, more mpox patients used PrEP to prevent HIV infection (OR = 2.03, 95% CI: 1.24–3.33).

We conducted a detailed study on the lesions of the two groups of patients. The lesions of mpox patients were more distributed in the genitals (OR = 2.90, 95% CI: 2.16–3.89) and perianal region (OR = 3.20, 95% CI: 1.94–5.28) but less distributed in the palms (OR = 0.83, 95% CI: 0.73–0.94), soles (OR = 0.62, 95% CI: 0.54–0.71) and neck (OR = 0.78, 95% CI: 0.69–0.89). There was no significant difference in lesion distribution across the oral cavity (OR = 0.96, 95% CI: 0.52–1.74), face (OR = 0.99, 95% CI: 0.71–1.38), head (OR = 0.90, 95% CI: 0.80–1.02), arms (OR = 0.94, 95% CI: 0.72–1.24), legs (OR = 0.86, 95% CI: 0.72–1.03) or trunk (OR = 1.13, 95% CI: 0.74–1.72). We compared five types of lesions and found that pustules (OR = 2.08, 95% CI: 1.55–2.80) were more common in mpox patients, whereas maculae (OR = 0.64, 95% CI: 0.58–0.72) were more common in non-mpox patients. Numbers of patients who had papulae (OR = 0.86, 95% CI: 0.63–1.19), vesicles (OR = 1.03, 95% CI: 0.76–1.32), and scabs (OR = 1.12, 95% CI: 0.83–1.51) did not significantly differ between the two groups.

In terms of complications, our study revealed that patients with mpox were more prone to proctitis (OR = 4.13, 95% CI: 1.91–8.95), while bacterial infection (OR = 1.43, 95% CI: 0.39–5.27) and urethritis (OR = 2.81, 95% CI: 0.33–24.04) incidences were not significantly different between mpox patients and non-mpox patients. Systemic symptoms were more common in mpox patients (OR = 2.58, 95% CI: 1.56–4.27). In terms of specific symptoms, the symptoms that were most likely to occur in mpox patients included fever (OR = 2.38, 95% CI: 1.80–3.15), lymphadenopathy (OR = 3.41, 95% CI: 2.72–4.28), myalgia (OR = 1.72, 95% CI: 1.32–2.23) and diarrhea (OR = 2.24, 95% CI: 1.20–4.19), whereas symptoms such as cough (OR = 0.85, 95% CI: 0.72–1.00), headache (OR = 0.90, 95% CI: 0.80–1.02), sore throat (OR = 1.18, 95% CI: 0.88–1.59), asthenia (OR = 1.27, 95% CI: 0.92–1.75), fatigue (OR = 1.16, 95% CI: 0.60–2.22), nausea (OR = 0.92, 95% CI: 0.63–1.34) and arthralgia (OR = 1.07, 95% CI: 0.62–1.86) did not significantly differ between groups.

### Reporting biases

Egger**’**s test was used for reporting bias analysis, and the results revealed that most of our findings had a low risk of bias (**Figure 3** and **Supplementary Figures S55**-**S96**).

**Figure 3:**
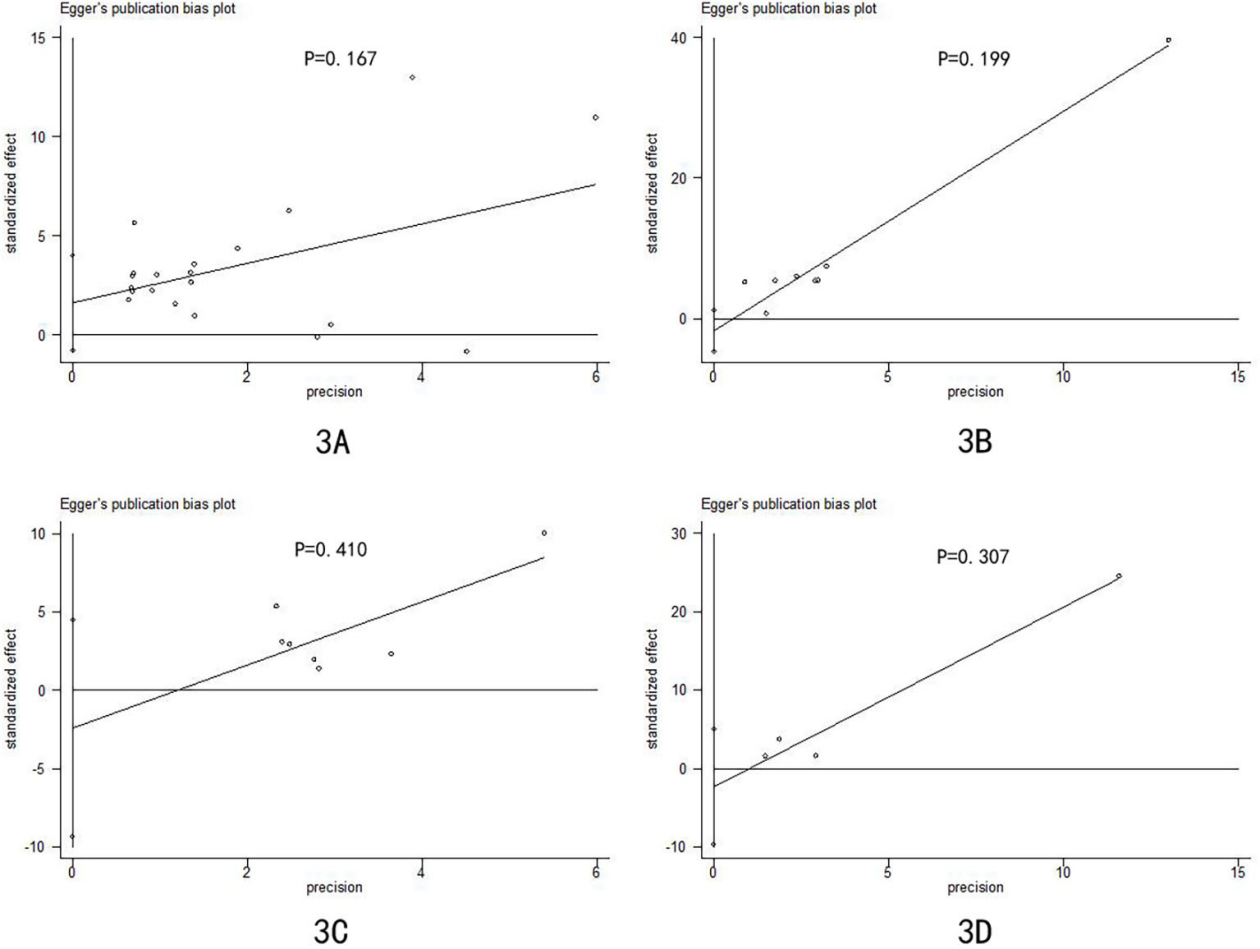
Egger’s publication bias plot of differences between mpox patients and non-mpox patients: **(A)** sex; **(B)** men who have sex with men; **(C)** multiple sexual partners; and **(D)** transmission through sexual contact.

### Heterogeneity

Most of our results showed low or moderate heterogeneity. For a few results with high heterogeneity and data from more than five included studies, we attempted to perform subgroup analysis within the continents where the included studies were located to explore the sources of heterogeneity. With respect to the results shown in **Supplementary Figure S18** and **Supplementary Figure S44**, the heterogeneity between subgroups decreased significantly, indicating that the regional differences between studies were the main source of heterogeneity.

### Sensitivity analysis

**Figure 4:**
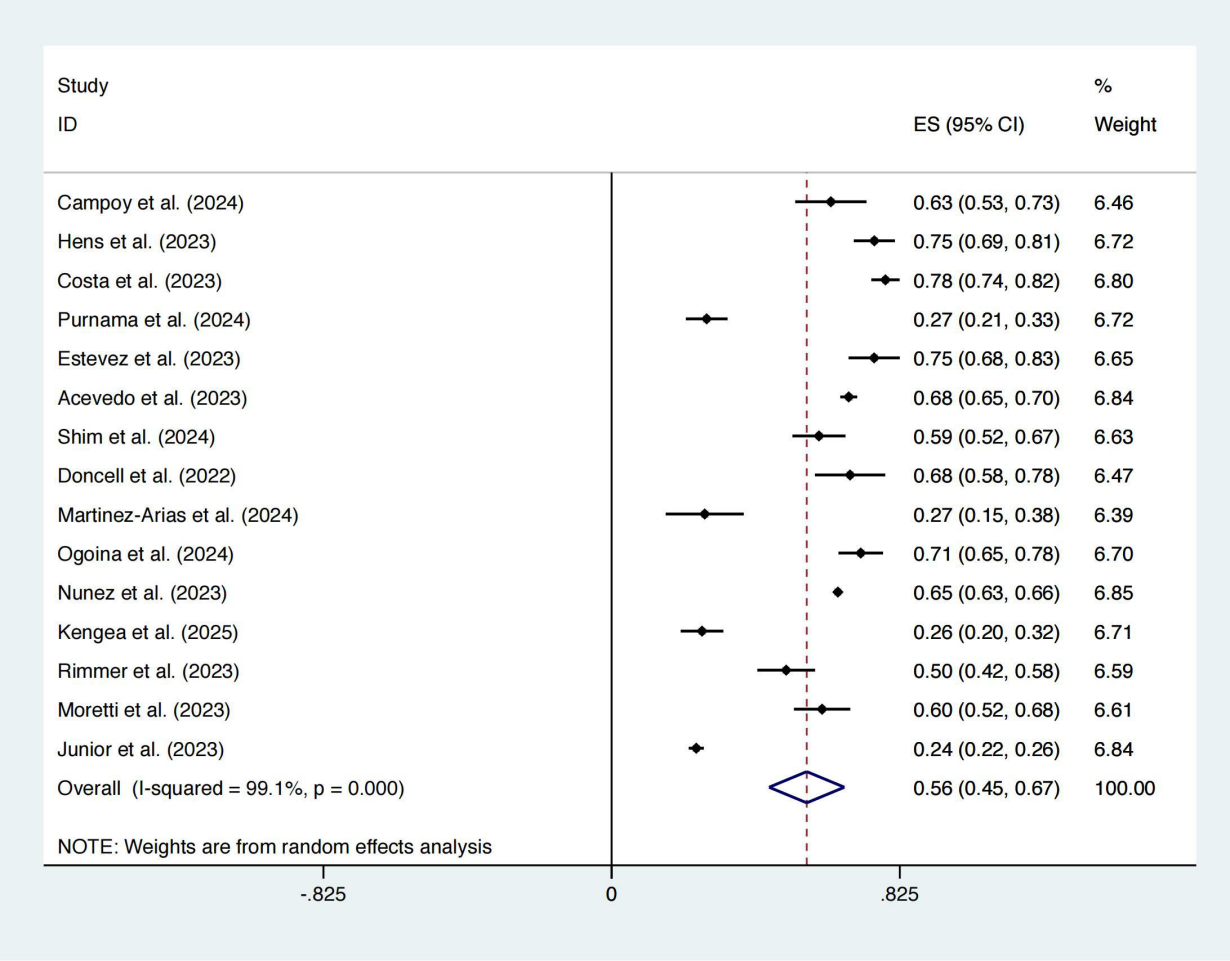
Forest plot of sensitivity analysis conducted after excluding studies with insufficient definitional information: prevalence.

A total of seven studies fell into the category of studies with insufficient definitional information (Arboleda et al., 2023; Brosius et al., 2025; Heukelom et al., 2023; Hohan et al., 2024; Lucero-Obusan et al., 2024; Mortier et al., 2023; Silva et al., 2022). After sensitivity analysis, the prevalence was 56% (95% CI: 45%–67%; I² = 99.1%; **Figure 4**), with no significant change in heterogeneity, indicating high robustness.

Our study analyzed more than 50 variables. To facilitate comparison with the sensitivity analysis results, we included these findings in **Table 2** rather than presenting them separately. For some variables, since studies with insufficient definitional information were not included in the quantitative synthesis, no sensitivity analysis was performed. For other variables, because only one study remained after removing studies with insufficient definitional information, no sensitivity analysis was performed. For all variables with at least 10 included studies, the results of the sensitivity analysis demonstrated high robustness, with no significant changes in OR values or heterogeneity. For some variables with fewer included studies, excluding studies with insufficient definitional information significantly altered their OR values or heterogeneity. This might be because, for those variables, the relatively small number of included studies combined with the high proportion of excluded studies meant that individual studies might have a significant influence on the combined results, leading to notable changes in the sensitivity analysis results.

## Discussion

The recent global mpox outbreaks attracted significant attention from the field of evidence-based medicine (Brochero et al., 2025). Our study focused on the epidemiological and clinical differences between mpox and non-mpox patients among suspected cases. The prevalence of mpox was as high as 54%, which was much higher than the infection risk of the general population. It is highly reasonable and accurate for clinicians to list such patients as suspected cases, reflecting an active and dense virus transmission network. More than half of the suspected cases were confirmed, emphasizing a necessity for maintaining a high level of vigilance regarding such epidemiological and clinical characteristics.

Our study revealed that the predominance of men among the confirmed patients (OR = 9.94) and the large OR of MSM (OR = 11.52) were the most significant demographic characteristics compared with patients without mpox. In terms of transmission routes, sexual contact appeared to be the primary mode of virus spread during and after the 2022 outbreak of mpox. Nonsexual contact and transmission through health services were relatively rare. During June and July 2022, adult mpox virus infections were detected at four educational institutions in England. However, no secondary cases occurred among the 340 exposed students and more than 100 exposed staff members (Ladhani et al., 2022). A study from Colorado, USA, revealed that 313 healthcare workers who had different degrees of contact with mpox patients during patient care or through contaminated materials did not have mpox transmission (28% of healthcare workers were considered to have had high- or intermediate-risk exposures) (Marshall et al., 2022). However, in addition to sexual behavior, other, unexpected types of transmission do exist. For example, poor hygiene practices/instrument cleaning was associated with 20 mpox cases associated with a Spanish tattoo and piercing establishment (Garcia et al., 2022).

The exposure findings are consistent with sexual transmission as a key route: confirmed patients were more likely to report recent sexual intercourse, multiple sexual partners, and anal sex. The sexual networks among MSM are similar to those of other groups, but the core groups are more densely connected, with frequent partner changes and multiple concurrent partners, reducing the likelihood of the virus encountering transmission barriers (Kupferschmidt, 2022). Together, these behavioral factors are consistent with an efficient virus transmission model: dense sexual networks may facilitate transmission pathways; having multiple sexual partners could expand the number of potentially exposed individuals; and anal sex might be associated with a higher likelihood of transmission due to the fragile mucosal tissue of the anus, which is more susceptible to micro- or macrodamage.

Our study revealed that the prevalence of HIV, syphilis and gonorrhea in confirmed patients was significantly greater, suggesting the importance of screening for STIs during the evaluation of suspected mpox cases and highlighting the well-established fact that having an STI amplifies the risk of acquiring additional STIs (Gandhi et al., 2019). Notably, among HIV-negative people, more mpox patients used PrEP. However, this association does not imply that PrEP drugs increase mpox susceptibility. It may be explained by the disproportionately high proportion of MSM among the mpox patients. Costa et al. reported that PrEP users tended to have more sexual partners, and this measure was adopted mainly by MSM with a university education level or who had completed higher education (Costa et al., 2023). The high prevalence of proctitis in mpox patients is also related to MSM. A study by Hens et al. revealed that the anorectum was the site of most intense viral replication in patients with mpox who reported anal sex (Hens et al., 2023). Valerio et al. reported that it was important for doctors to test for mpox in any patient with proctitis among MSM. A rectal swab should be taken for mpox virus PCR, even if the patient does not appear to have mucocutaneous lesions at the time (Valerio et al., 2023).

One point that needs to be emphasized is that although the finding that MSM is significantly associated with mpox infection has certain guiding value for concentrating public health resources and implementing precise interventions (such as targeted health education, vaccination, and promotion of pre-exposure prophylaxis), this epidemiological characteristic must be viewed with caution to avoid stigma and discrimination against the MSM community. In fact, some countries and regions even criminalize same-sex relationships among MSM (Ogoina et al., 2024). The WHO has warned that stigma toward specific groups may hinder patients from seeking timely medical treatment, undermine community trust, and ultimately weaken the effectiveness of epidemic prevention (WHO, 2022b). Importantly, mpox can infect individuals of any sexual orientation. Public health information transmission and intervention measures should not only effectively identify and cover high-risk groups but also balance ethical principles that promote social inclusion and ensure health equity.

Skin lesions in confirmed patients were primarily concentrated in the genital and perianal areas. This “genital–perianal” distribution is somewhat consistent with the hypothesis of “inoculation transmission”, which suggests that during sexual contact, the virus may be directly inoculated into tiny wounds in these parts through close friction between skin and skin or between skin and mucosa, thus completing initial replication and causing lesions (Patrocinio-Jesus and Peruzzu, 2022). In contrast, the lesions of patients without mpox were more common in the palms, soles and neck. This distribution was more in line with the typical manifestations of other pathogens (such as hand-foot-mouth disease) or noninfectious diseases (such as atopic dermatitis) (Esposito and Principi, 2018; Ramirez-Marin and Silverberg, 2022). We also found that pustules were more common in mpox patients, which was in line with the natural course of mpox virus infection. Mpox skin lesions usually go through the stages of maculae, papulae, vesicles, pustules and scabs. The appearance of pustules marks the inflammatory reaction peak and is a relatively specific stage of the disease (Lim et al., 2024).

In terms of symptoms, mpox patients showed stronger characteristics of systemic viremia. Fever, lymphadenopathy, myalgia and diarrhea are all signs that the virus has spread in the body and has triggered a systemic immune response. Among these, lymphadenopathy is considered to be a key clinical characteristic that distinguishes mpox from chickenpox (Patel et al., 2023). However, respiratory symptoms such as cough and sore throat did not significantly differ between mpox patients and non-mpox patients, further reducing the importance of respiratory transmission during and after 2022 epidemic.

Non-mpox patients and mpox patients may show some specific differences in their rashes according to different causes. Taking viral infection as an example, the cutaneous manifestations of secondary syphilis are diverse. In addition to presenting as papulae or pustules, it can also present as condylomata lata, mucous patches, or split papulae. The latter manifestations are specific to syphilis, as no such similarities are found in mpox; thus, these are among the clinical characteristics that differentiate mpox and syphilis (Cohen et al., 2013). Moreover, secondary syphilis rashes are not pruritic and can be minimal enough to be ignored. At the same time, lesions related to mpox are usually painful (Hussain et al., 2022). Herpes zoster lesions often appear everywhere in waves, and the pain intensity is much greater than that of mpox lesions. In some individuals, the pain may be so severe that anesthetic administration is needed (Freer and Pistello, 2018). In many cases, the pain persists for several months after the rash subsides in a phenomenon termed postherpetic neuralgia (Hussain et al., 2022). Once all lesions form scabs, herpes zoster lesions are usually regarded as having low infectivity, even though viral DNA is still recoverable by PCR at this stage (Mols et al., 2013). In contrast, scabs associated with mpox lesions are considered highly infectious until complete re-epithelialization occurs (Cowen et al., 2023).

In our study, various confounding factors may have influenced the pooled estimates to varying degrees. Owing to the increased prevalence of behaviors such as dense sexual networks and multiple sexual partners among young MSM populations, age may indirectly regulate certain ORs by affecting sexual behavior patterns. Our study revealed no statistically significant difference in age between mpox and non-mpox patients, suggesting that age might not be a strong confounding factor in our sample. We conducted a subgroup analysis by region and found that mpox prevalence varied significantly across continents (60% in South America and 44% in Asia). In our heterogeneity analysis, we found that for a few highly heterogeneous results, regional subgroup analysis could significantly reduce heterogeneity, supporting regions as an important moderating variable. Nevertheless, our sample included data from 16 countries, which provided good geographical diversity, and most results showed low to moderate heterogeneity. This suggests that regional differences might not be the primary confounding source of all associations; HIV infection is a potential confounder on which we focused. Our results showed that there were more HIV-infected individuals among mpox patients, and PrEP use was more common among HIV-negative individuals. This indicates that HIV infection or HIV-related risk behaviors (such as multiple sexual partners) might be collinear with mpox infection and that sexual behavior-related ORs might be partially confounded by the high-risk behaviors of the HIV-infected population.

### Limitations

Our study has two main limitations. First, there may be inherent biases in the case definition, as the criteria for identifying suspected mpox cases in the included studies were not uniform. Second, there may be information bias in this study. Information regarding recent exposure history and sexual behavior was obtained mainly through patient self-reports. This sensitive information may be affected by recall bias or social expectation bias (such as reluctance to report certain sexual behaviors), thereby influencing the accurate assessment of the strength of the association between relevant risk factors.

## Conclusions

We performed a detailed study on the epidemiological and clinical differences between mpox and non-mpox patients among suspected cases during the recent global mpox outbreaks, which provides some reference for clinicians in the diagnosis and differentiation of mpox. These differential characteristics are probabilistic indicators rather than definitive diagnostic tests. Their clinical utility lies in increasing or reducing the prior probability of clinical diagnosis, thereby optimizing the use of medical resources. During triage or initial diagnosis, especially when PCR testing is delayed, if suspected cases exhibit these characteristics, it is advisable to trigger isolation measures and prioritize testing.

The recent global mpox epidemic was a profound lesson, once again highlighting the complex mode of interaction between novel infectious diseases and specific social behavior networks. By scientifically understanding these models, healthcare workers can not only address this epidemic more effectively but also be more fully prepared for the inevitable challenges of future emerging infectious diseases.

## Data Availability

The original contributions presented in the study are included in the article/**Supplementary Material**. Further inquiries can be directed to the corresponding author.
