# Supplementary material for: The epidemiological and clinical differences between patients with mpox and patients without mpox among suspected cases during the recent global mpox outbreaks: a systematic review and meta-analysis
Source: Front Cell Infect Microbiol. 2026 Jan 12;15:1740617. doi: 10.3389/fcimb.2025.1740617 (PMC12832785; doi:10.3389/fcimb.2025.1740617)
Supplement: Supplementary file 1 [file DataSheet1.doc]

**Supplementary materials**

**Table S1 :** Newcastle–Ottawa Quality Assessment Scale.

| Study | Selection | Comparability | Outcome | Quality assessment |
| --- | --- | --- | --- | --- |
| Campoy et al., 2024 | 4 | 1 | 3 | Good |
| Silva et al., 2022 | 4 | 1 | 2 | Good |
| Arboleda et al., 2023 | 4 | 1 | 1 | Fair |
| Hens et al., 2023 | 4 | 2 | 3 | Good |
| Heukelom et al., 2023 | 3 | 1 | 3 | Good |
| Costa et al., 2023 | 4 | 1 | 3 | Good |
| Purnama et al., 2025 | 4 | 1 | 3 | Good |
| Estevez et al., 2023 | 4 | 1 | 3 | Good |
| Acevedo et al., 2023 | 4 | 2 | 2 | Good |
| Brosius et al., 2025 | 4 | 1 | 3 | Good |
| Shim et al., 2024 | 4 | 1 | 3 | Good |
| Lucero-Obusan et al., 2024 | 4 | 1 | 3 | Good |
| Doncell et al., 2022 | 4 | 1 | 3 | Good |
| Martinez-Arias et al., 2025 | 4 | 1 | 3 | Good |
| Ogoina et al., 2024 | 4 | 1 | 3 | Good |
| Nunez et al., 2023 | 4 | 1 | 3 | Good |
| Kengea et al., 2025 | 4 | 1 | 3 | Good |
| Rimmer et al., 2023 | 4 | 1 | 3 | Good |
| Moretti et al., 2023 | 4 | 1 | 3 | Good |
| Mortier et al., 2023 | 4 | 1 | 3 | Good |
| Junior et al., 2023 | 4 | 1 | 3 | Good |

**Table S2 : Joanna Briggs Institute Critical Appraisal Checklist.**

| Study | Clear criteria for inclusion | Consecutive inclusion | Uniform measurement | Valid identification of condition | Complete demographic reporting | Complete clinical reporting | Clear reporting of follow-up/outcomes | Sufficient follow-up | Clear presentation of results | Appropriate statistical methods |
| --- | --- | --- | --- | --- | --- | --- | --- | --- | --- | --- |
| Hohan et al., 2024 | Yes | Yes | Yes | Yes | Yes | Yes | Unclear | Unclear | Yes | Yes |


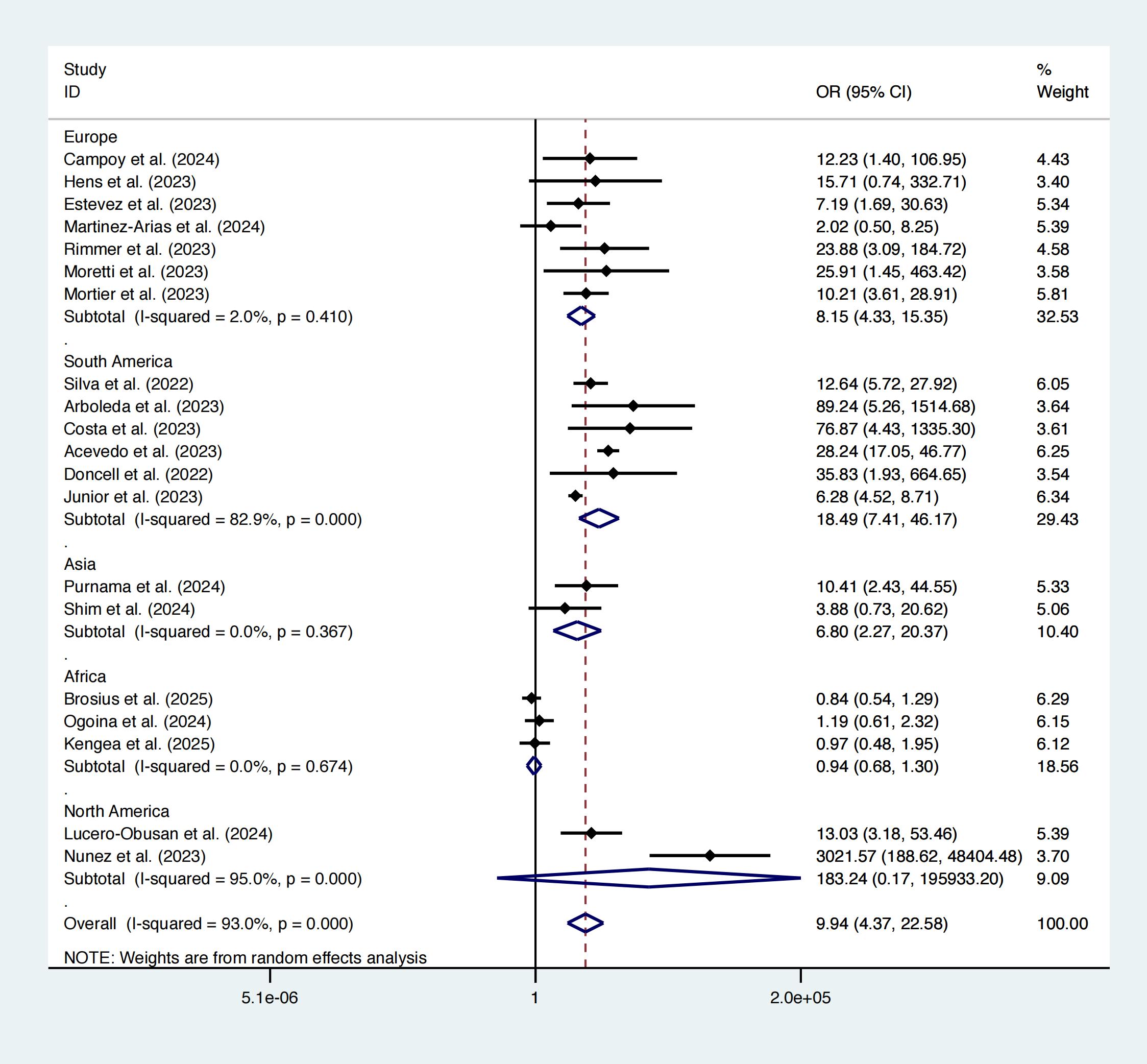


**Figure S1:** Forest plot of differences in sex between mpox patients and non-mpox patients.


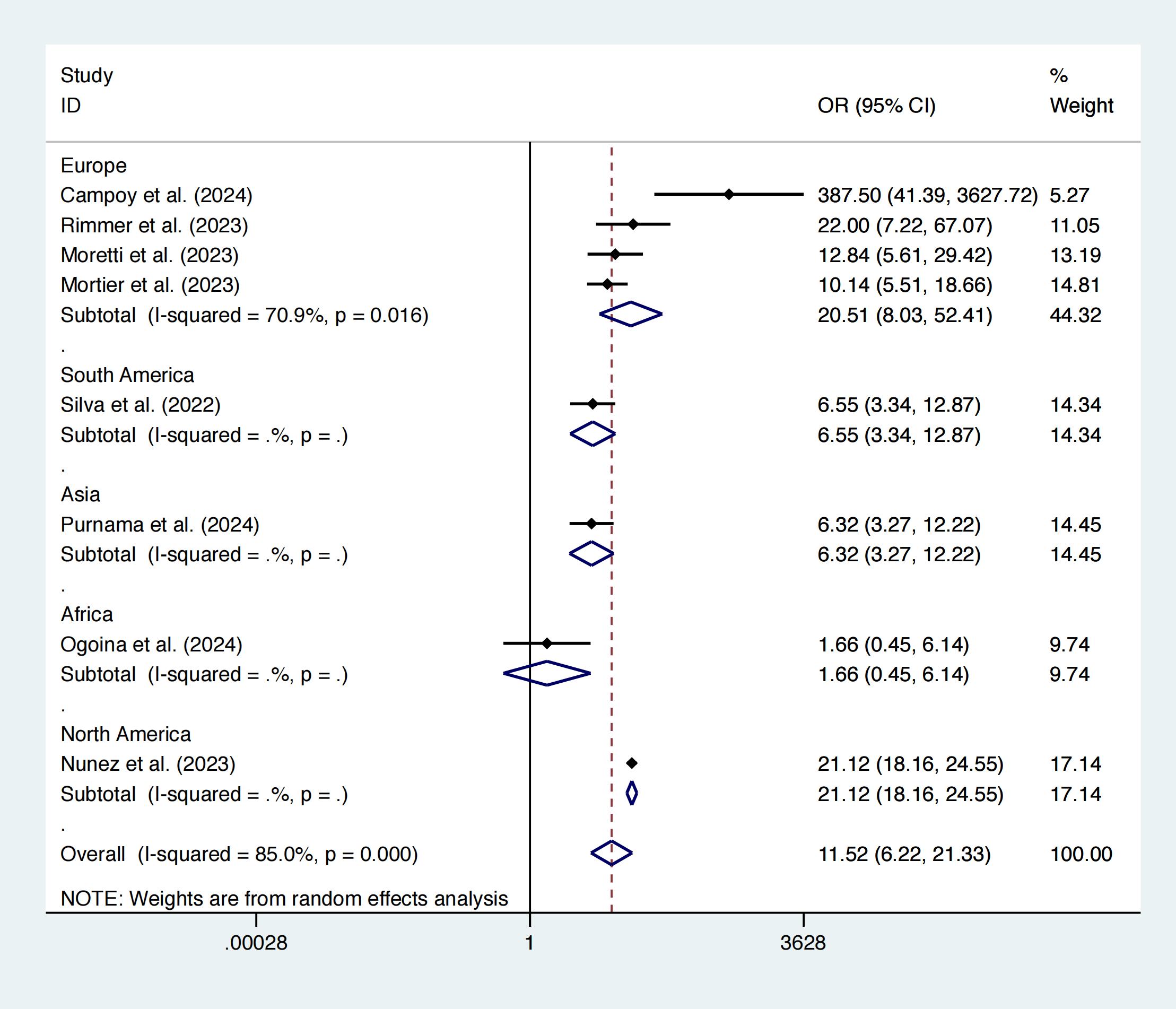


**Figure S2:** Forest plot of differences in men who have sex with men between mpox patients and non-mpox patients.


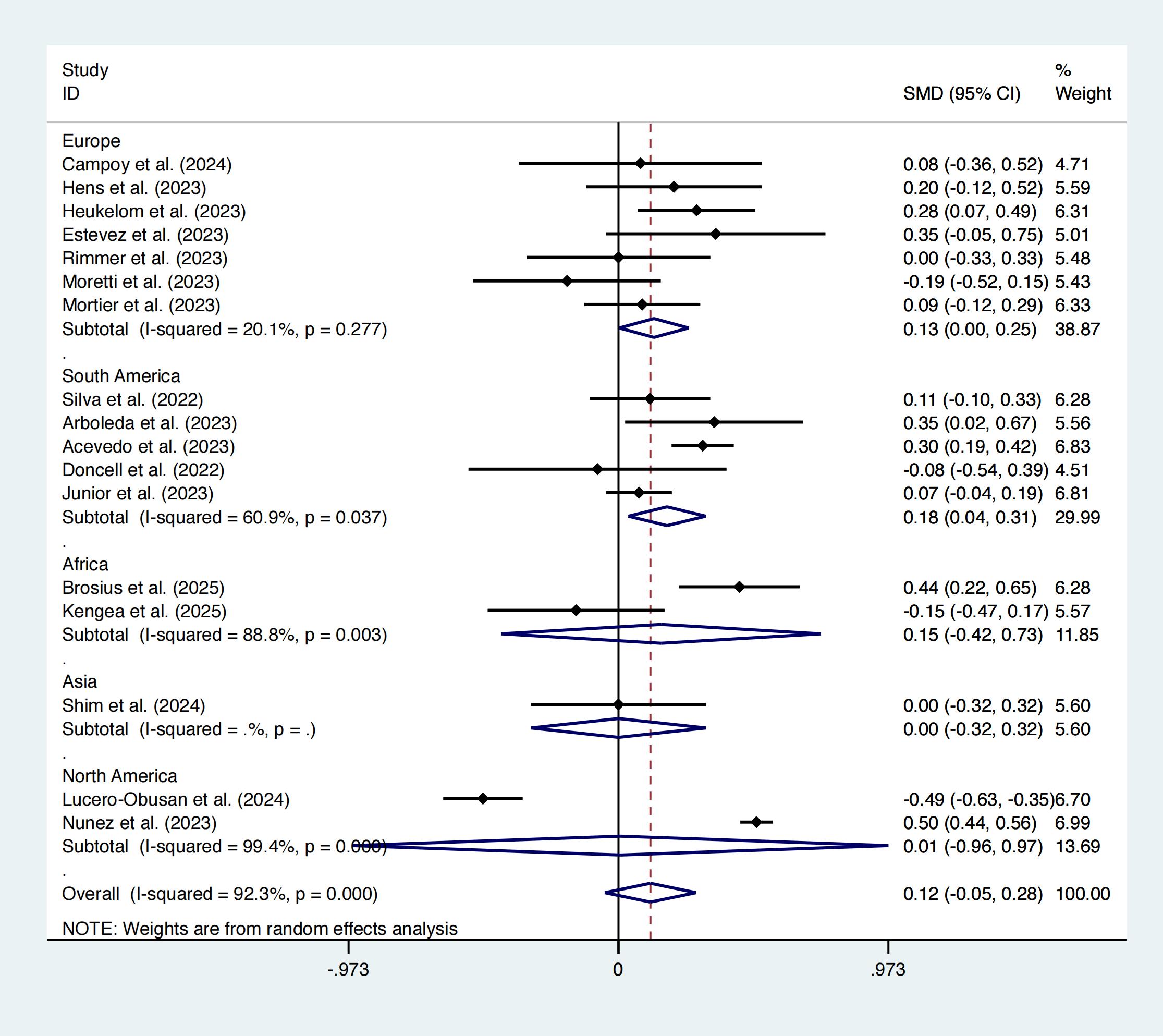


**Figure S3:** Forest plot of differences in age between mpox patients and non-mpox patients.


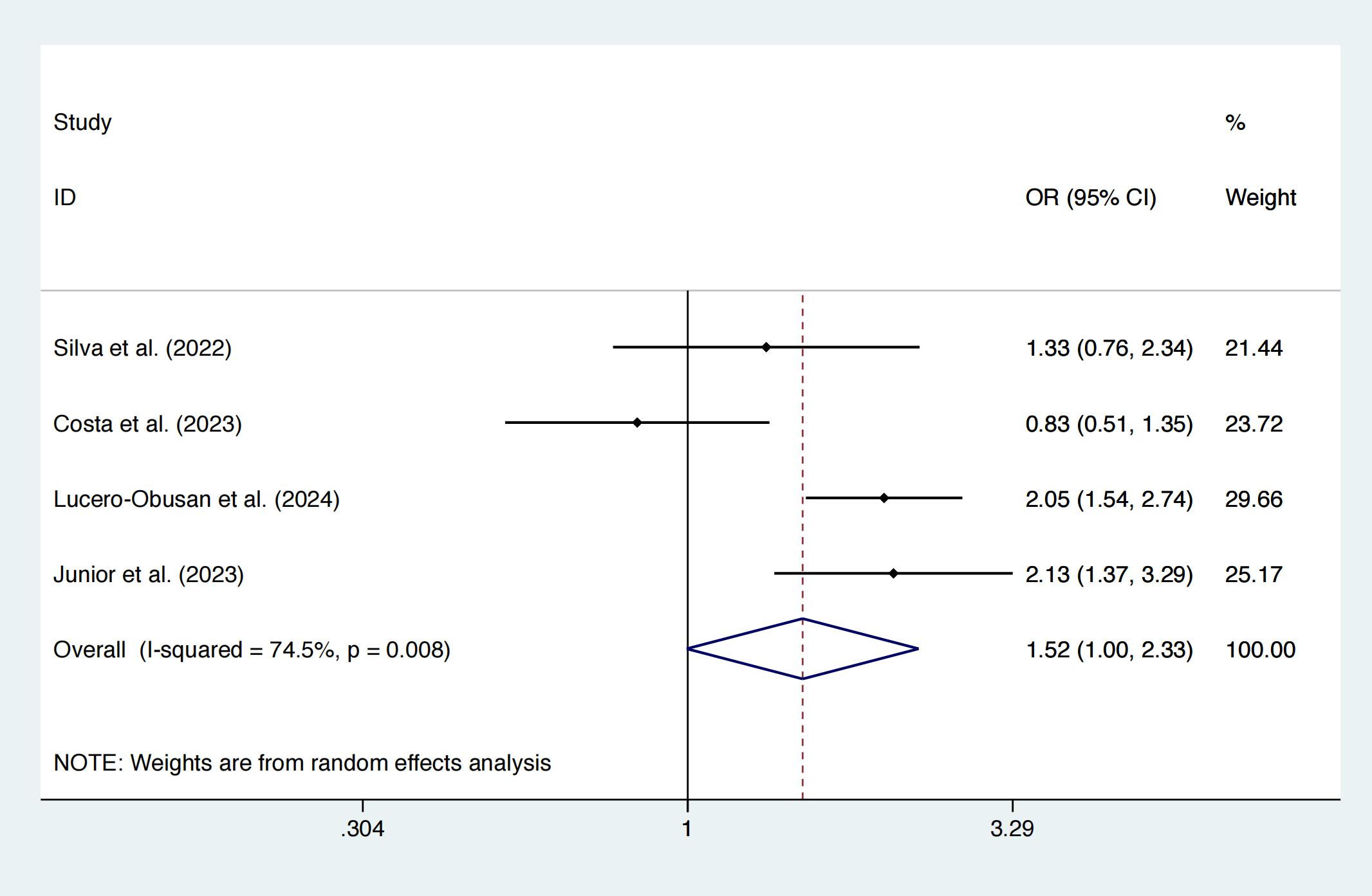


**Figure S4:** Forest plot of differences in race between mpox patients and non-mpox patients: Black.


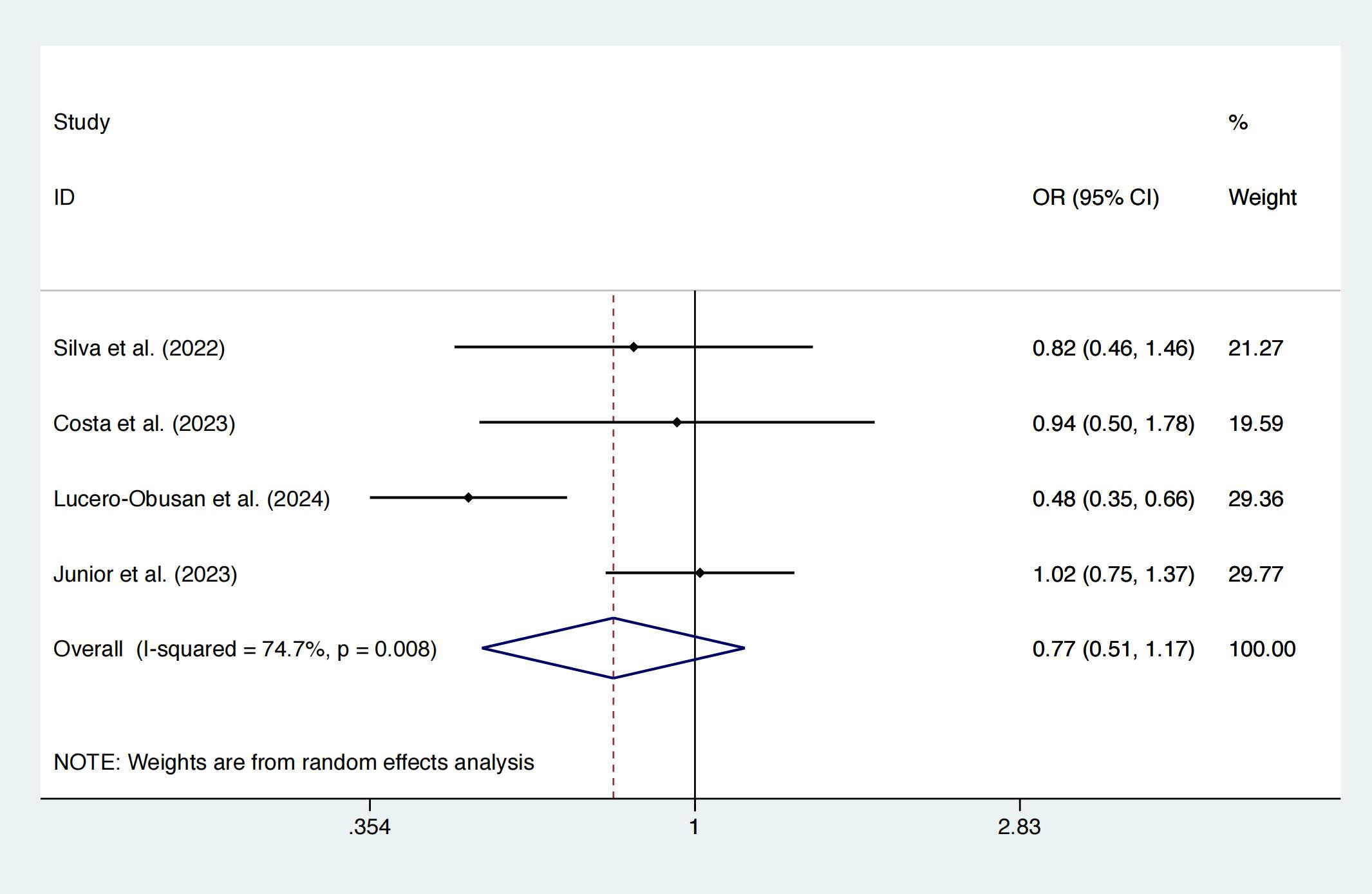


**Figure S5:** Forest plot of differences in race between mpox patients and non-mpox patients: White.


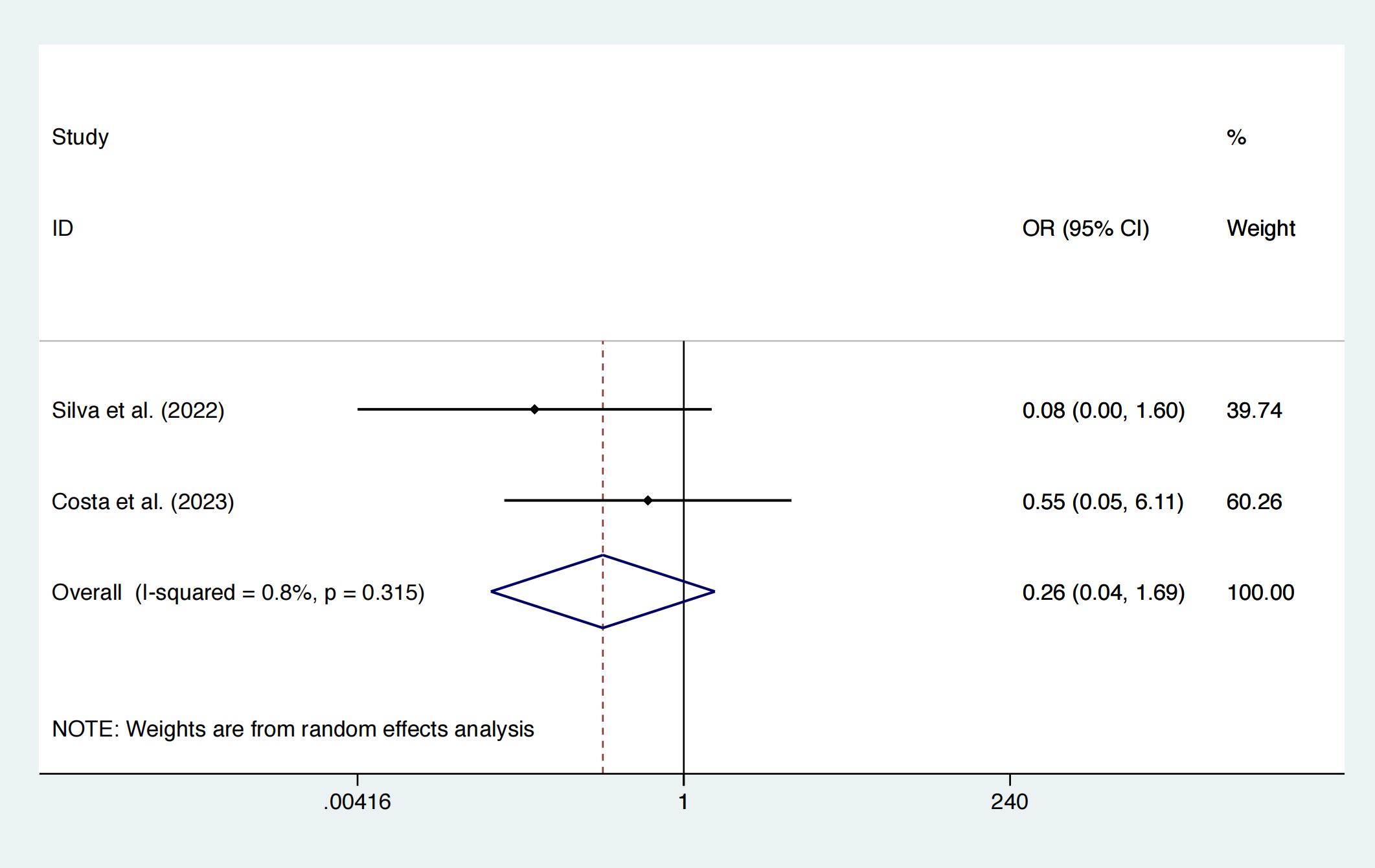


**Figure S6:** Forest plot of differences in race between mpox patients and non-mpox patients: Asian.


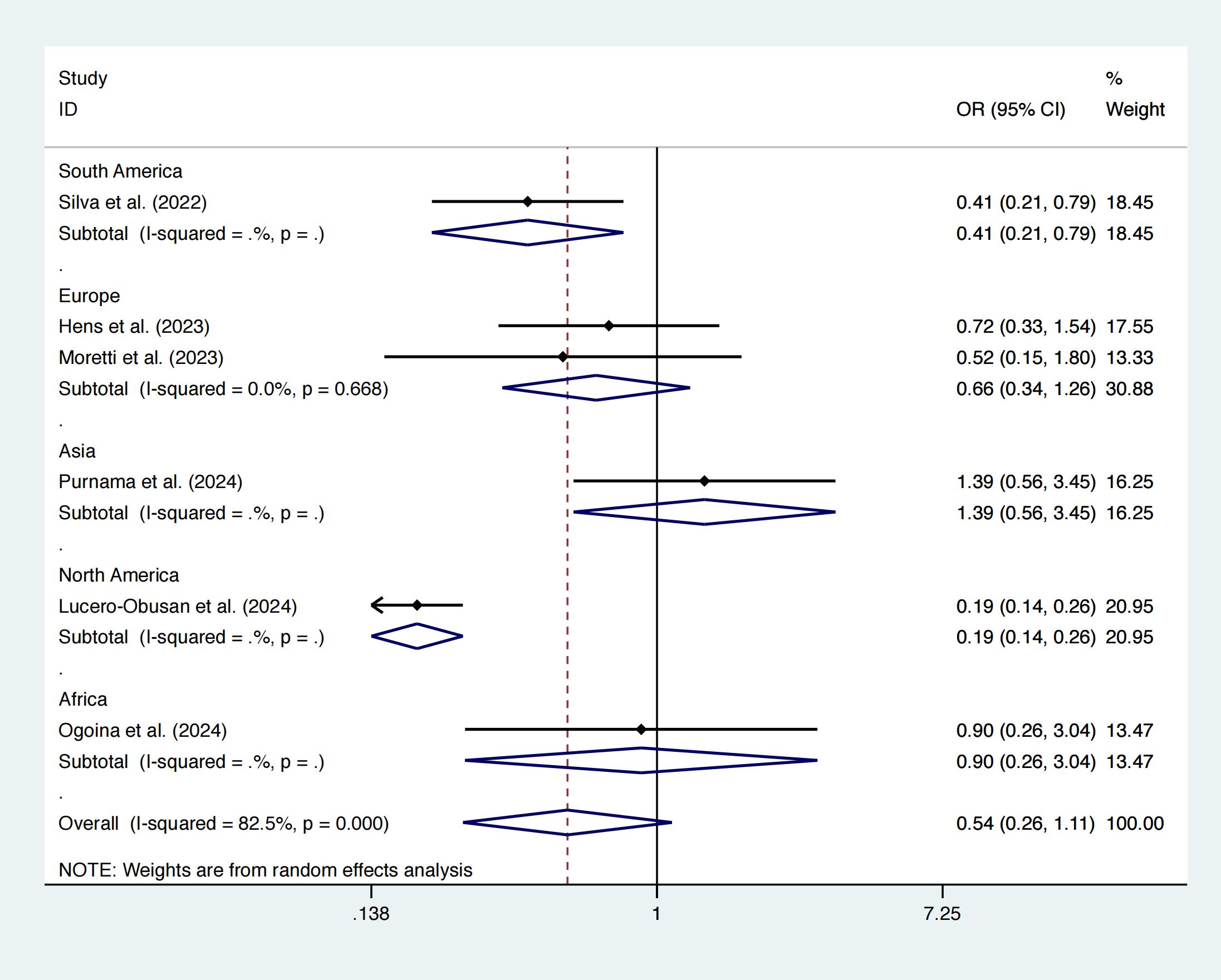


**Figure S7:** Forest plot of differences in previous mpox vaccination between mpox patients and non-mpox patients.


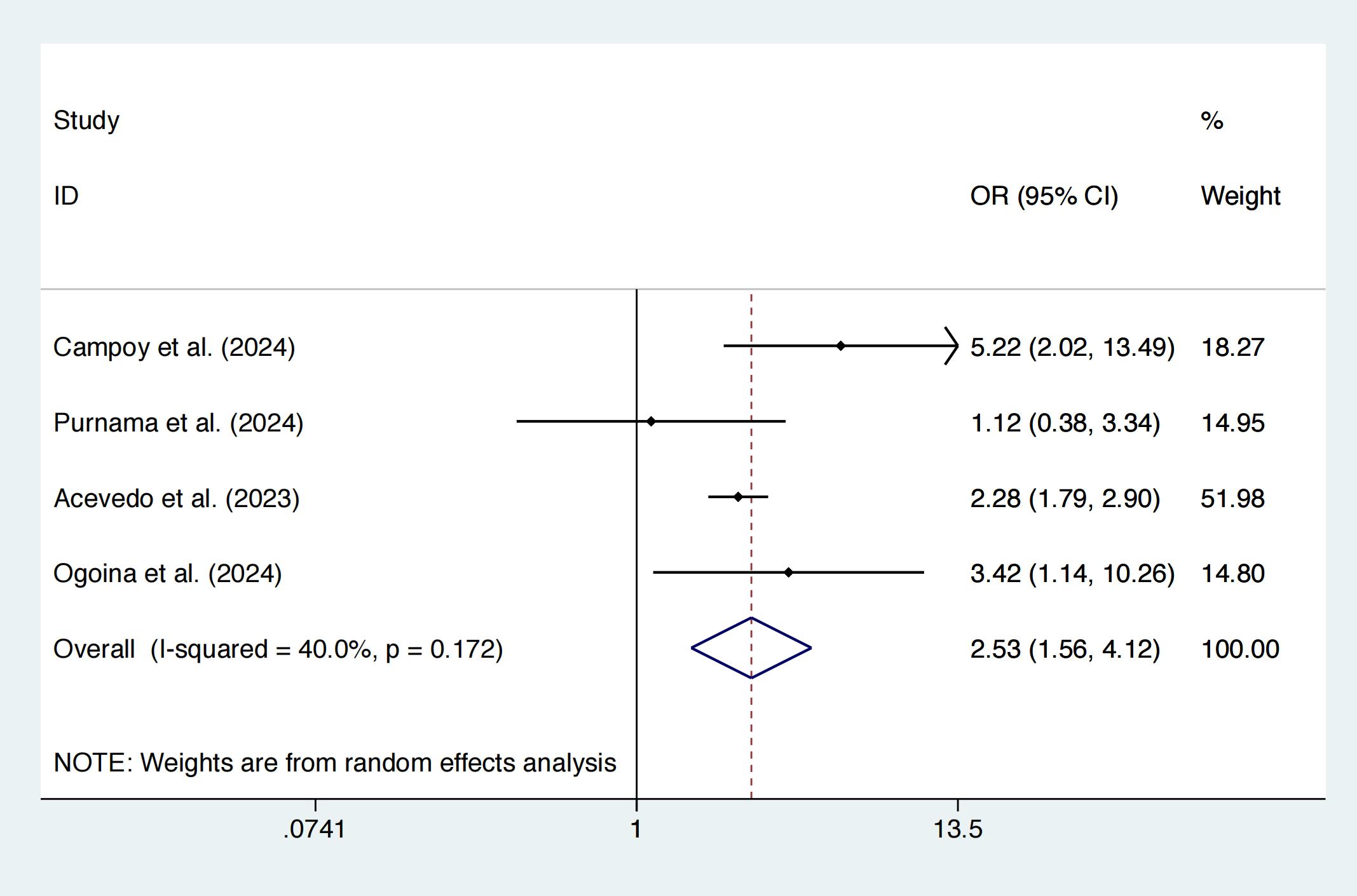


**Figure S8:** Forest plot of differences in recent exposure between mpox patients and non-mpox patients: contact with previous mpox patients.


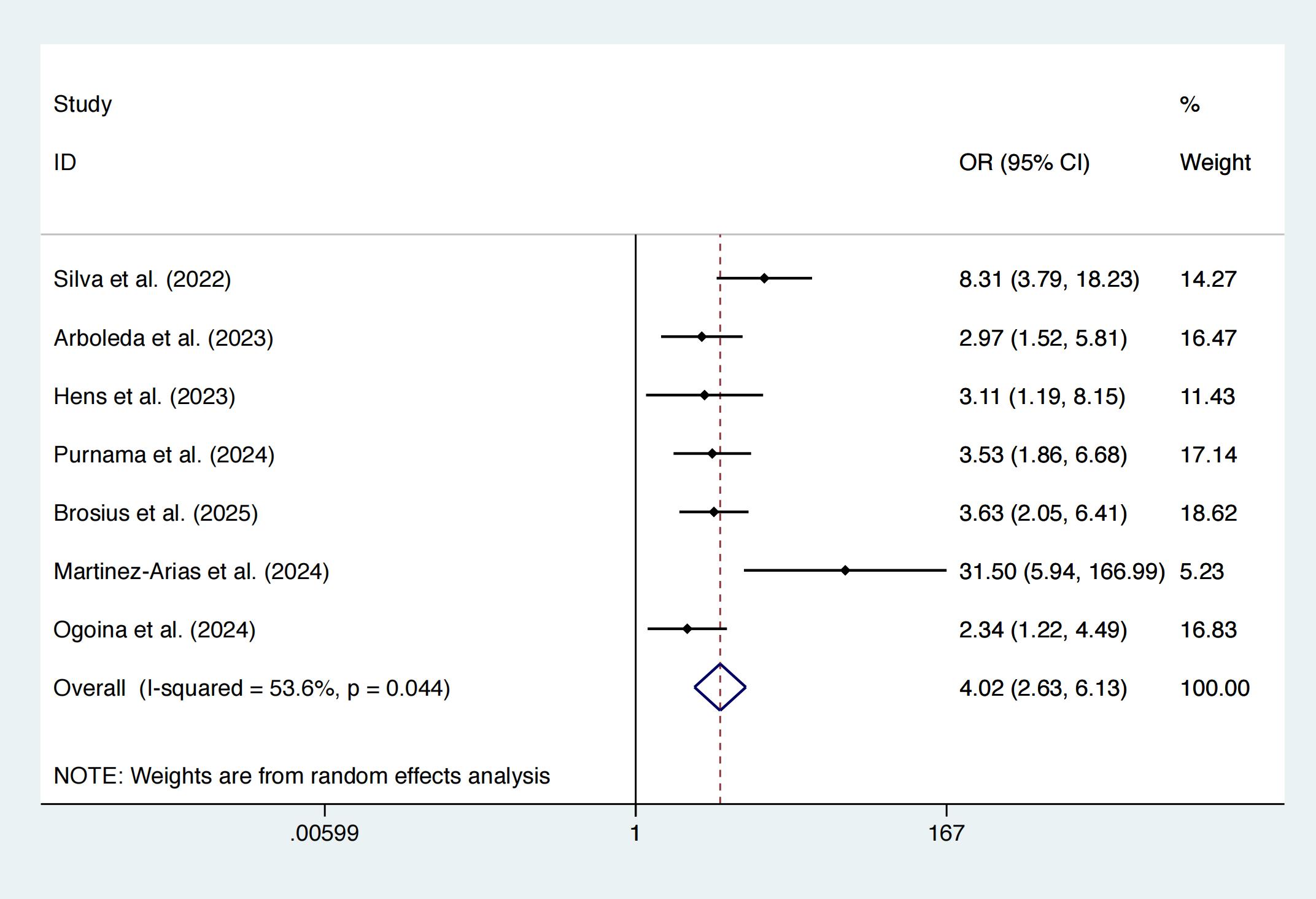


**Figure S9:** Forest plot of differences in recent exposure between mpox patients and non-mpox patients: sexual intercourse.


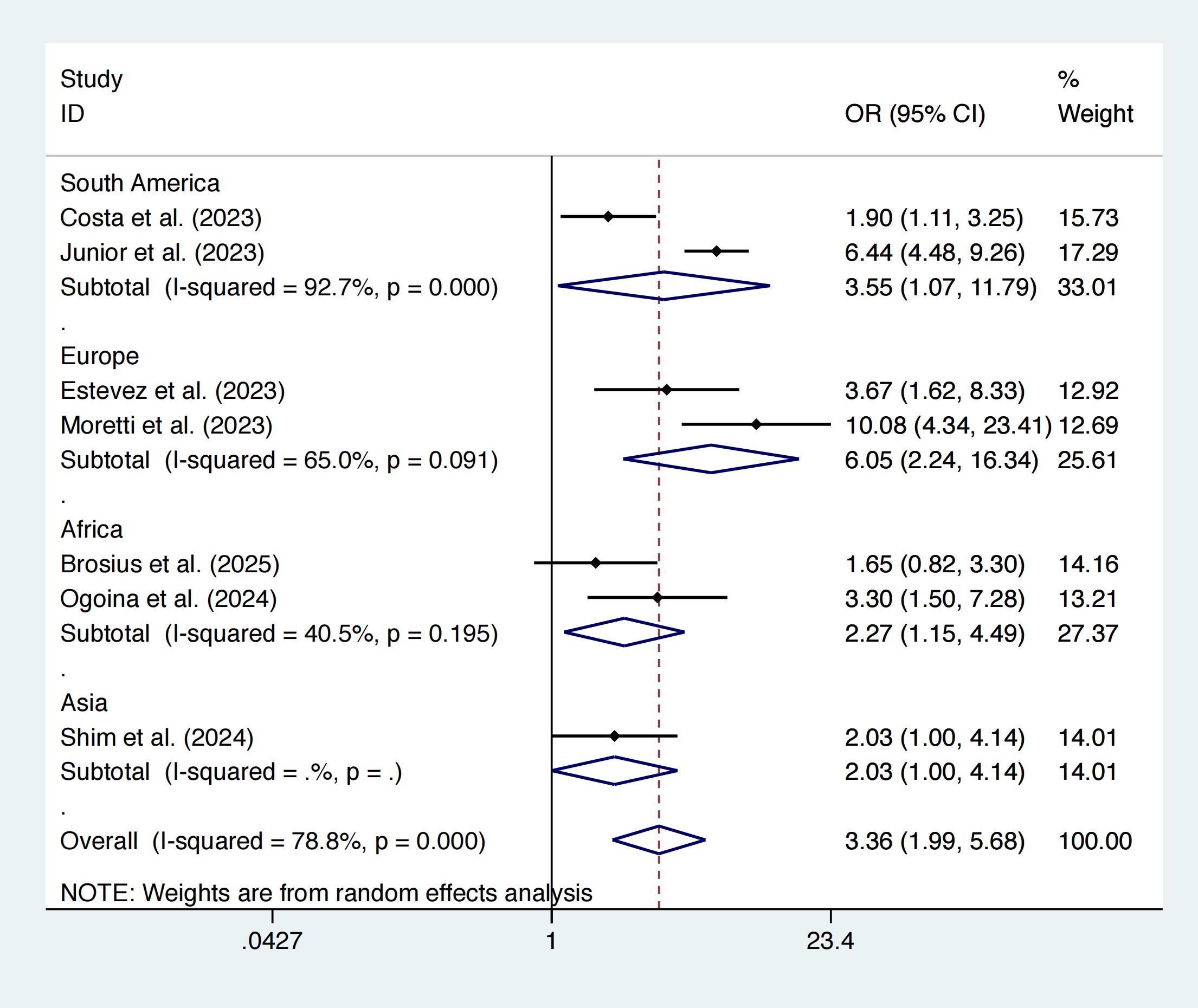


**Figure S10:** Forest plot of differences in recent exposure between mpox patients and non-mpox patients: multiple sexual partners.


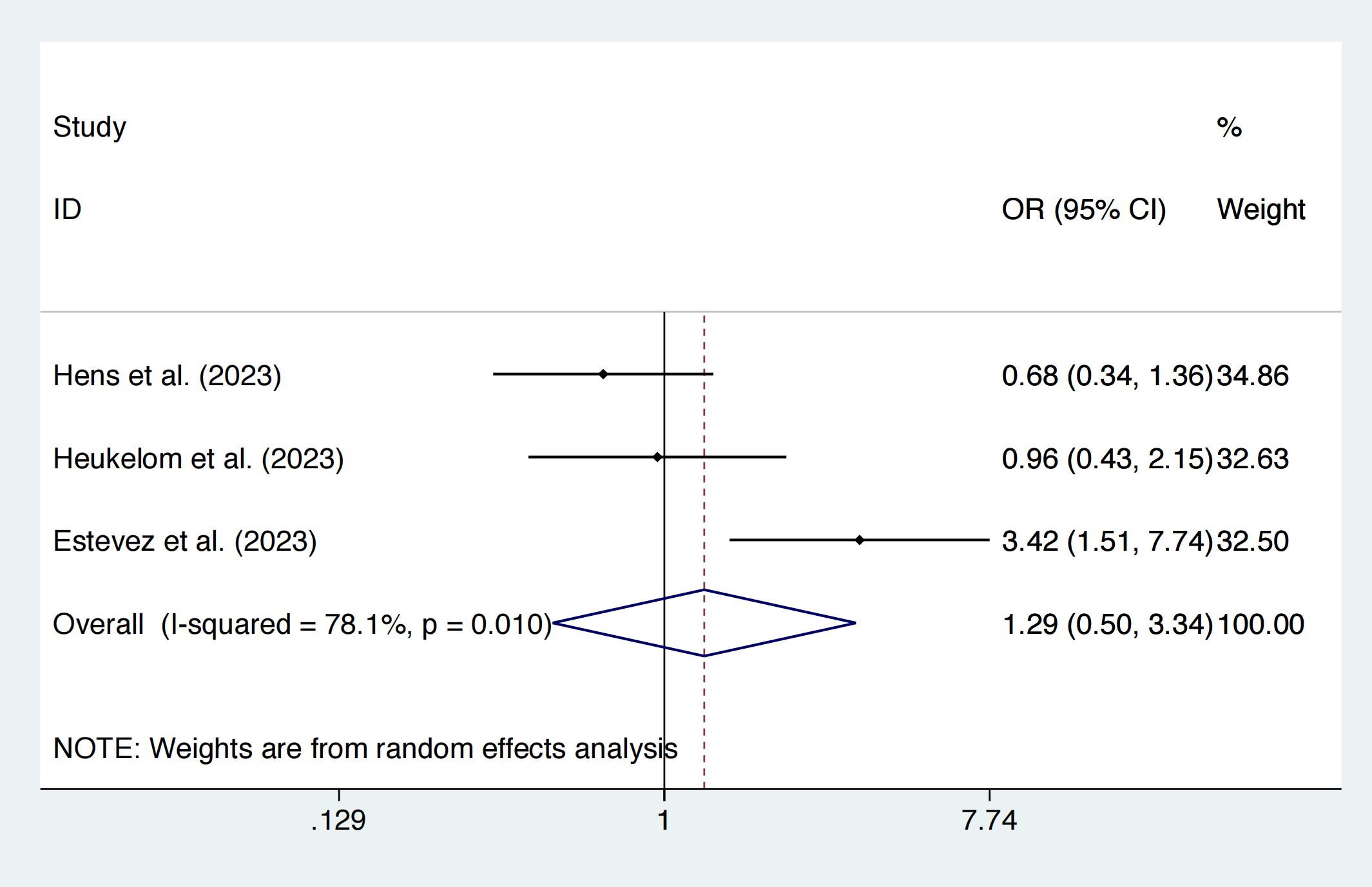


**Figure S11:** Forest plot of differences in recent exposure between mpox patients and non-mpox patients: oral sex.


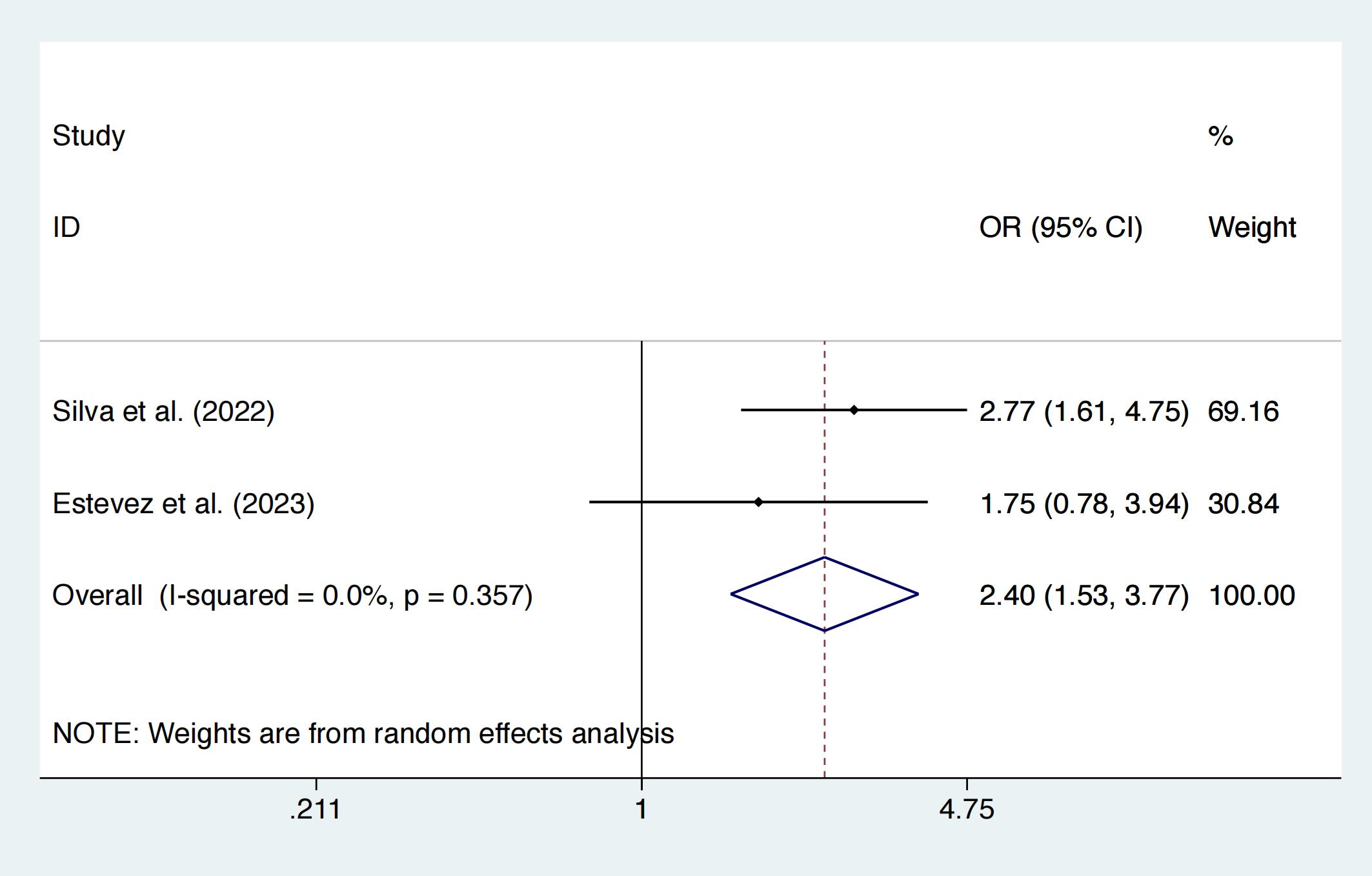


**Figure S12:** Forest plot of differences in recent exposure between mpox patients and non-mpox patients: anal sex.


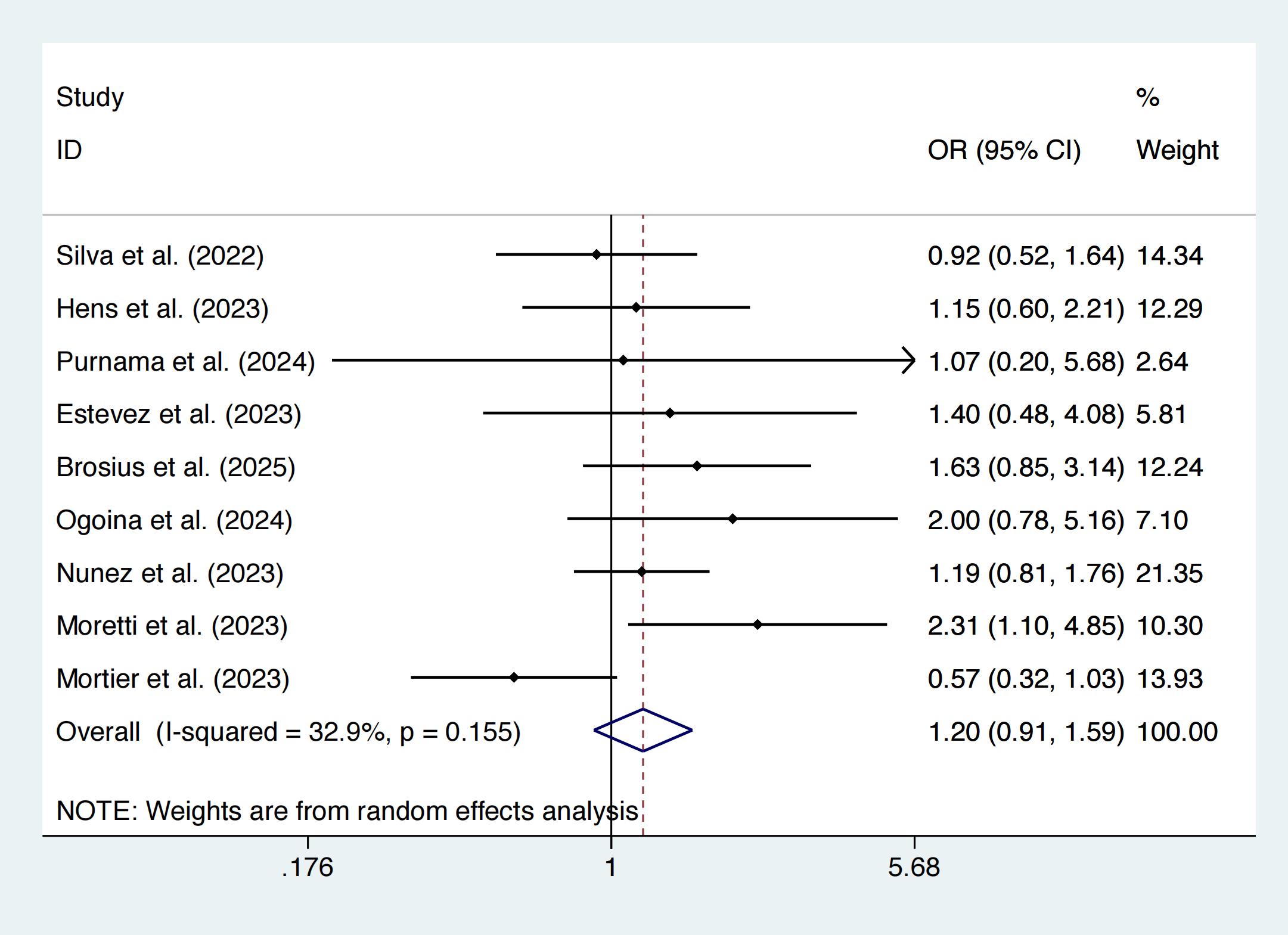


**Figure S13:** Forest plot of differences in recent exposure between mpox patients and non-mpox patients: travel.


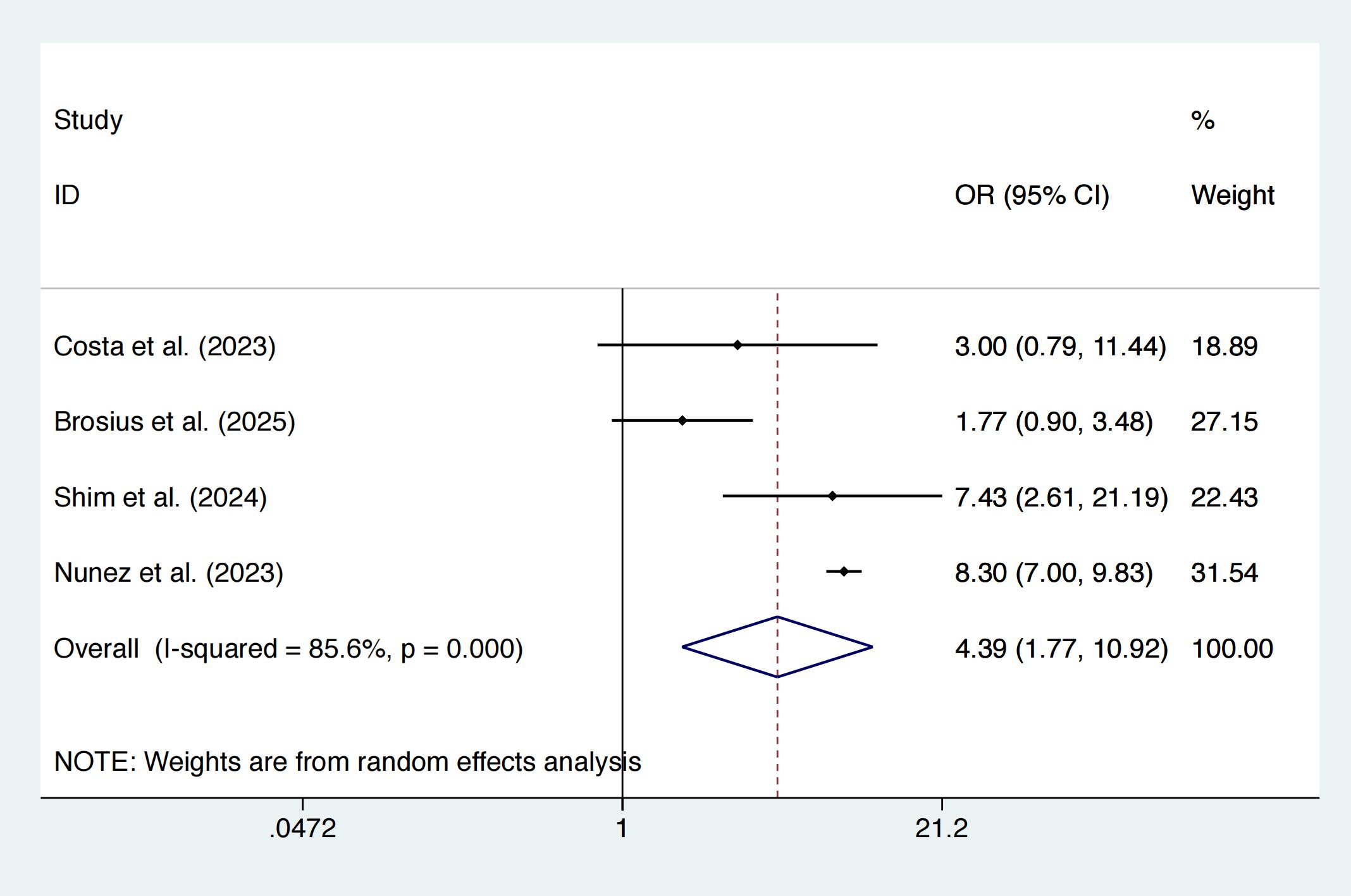


**Figure S14:** Forest plot of differences in transmission routes between mpox patients and non-mpox patients: sexual contact.


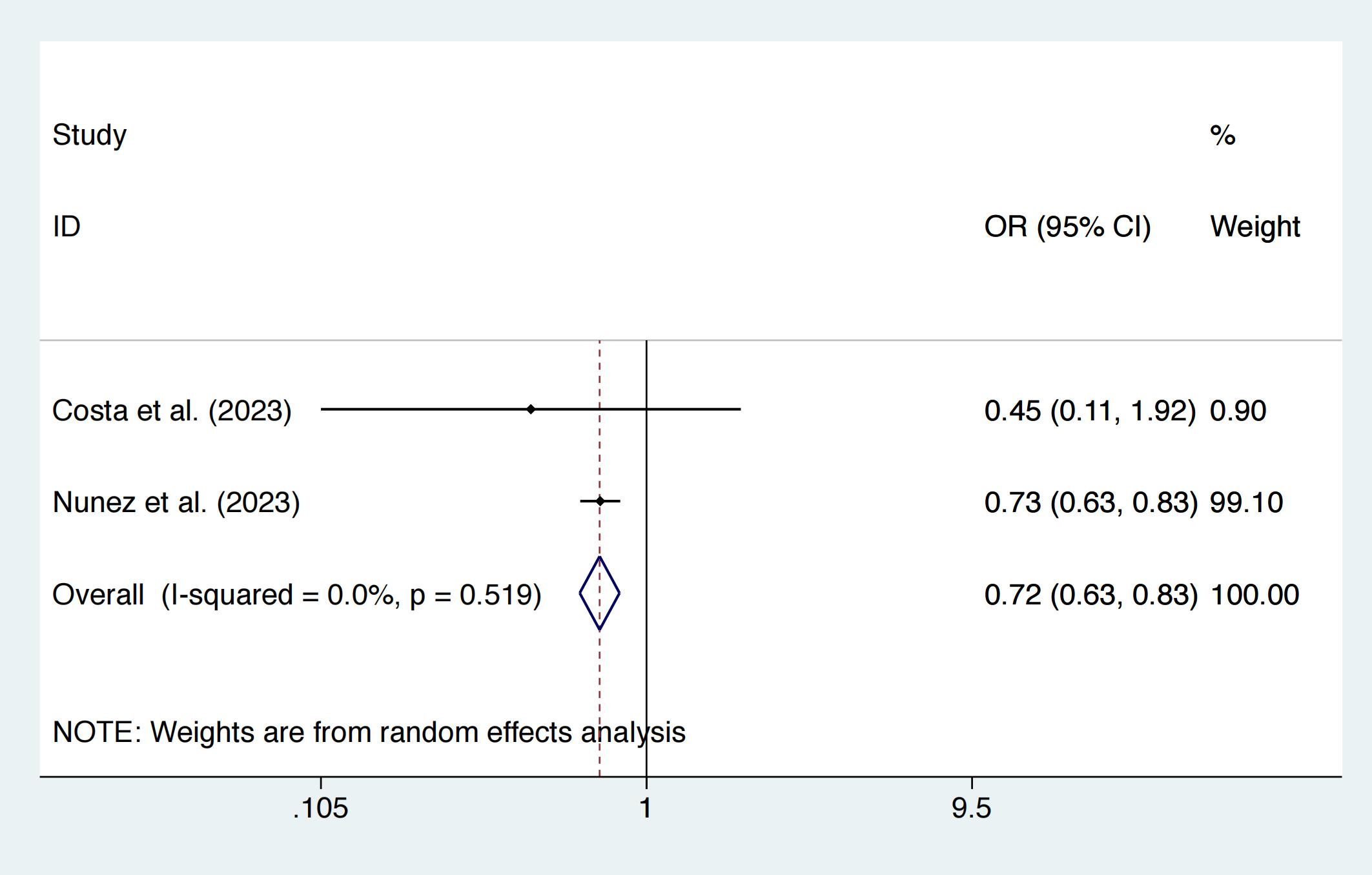


**Figure S15:** Forest plot of differences in transmission routes between mpox patients and non-mpox patients: nonsexual contact.


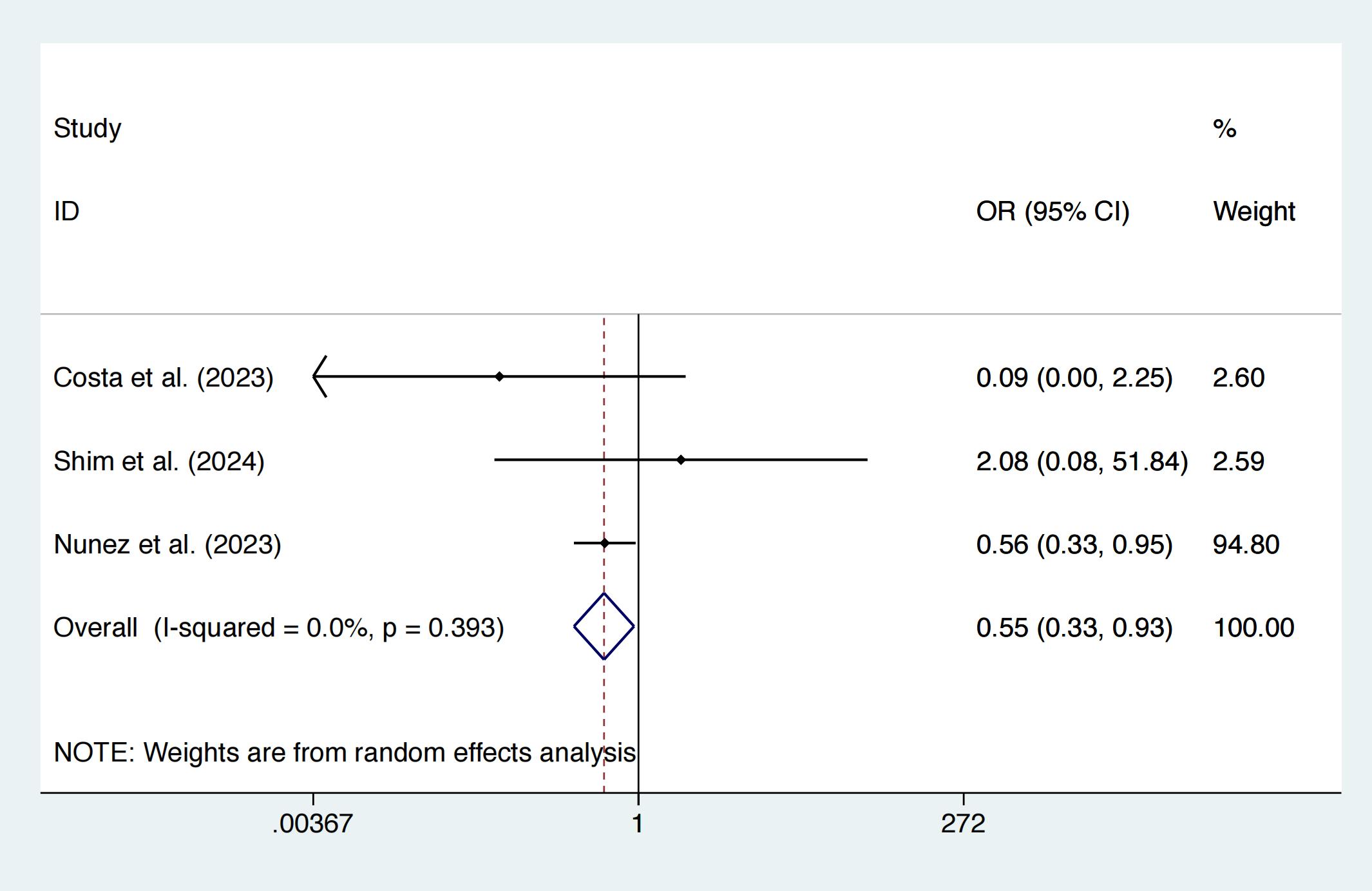


**Figure S16:** Forest plot of differences in transmission routes between mpox patients and non-mpox patients: health services.


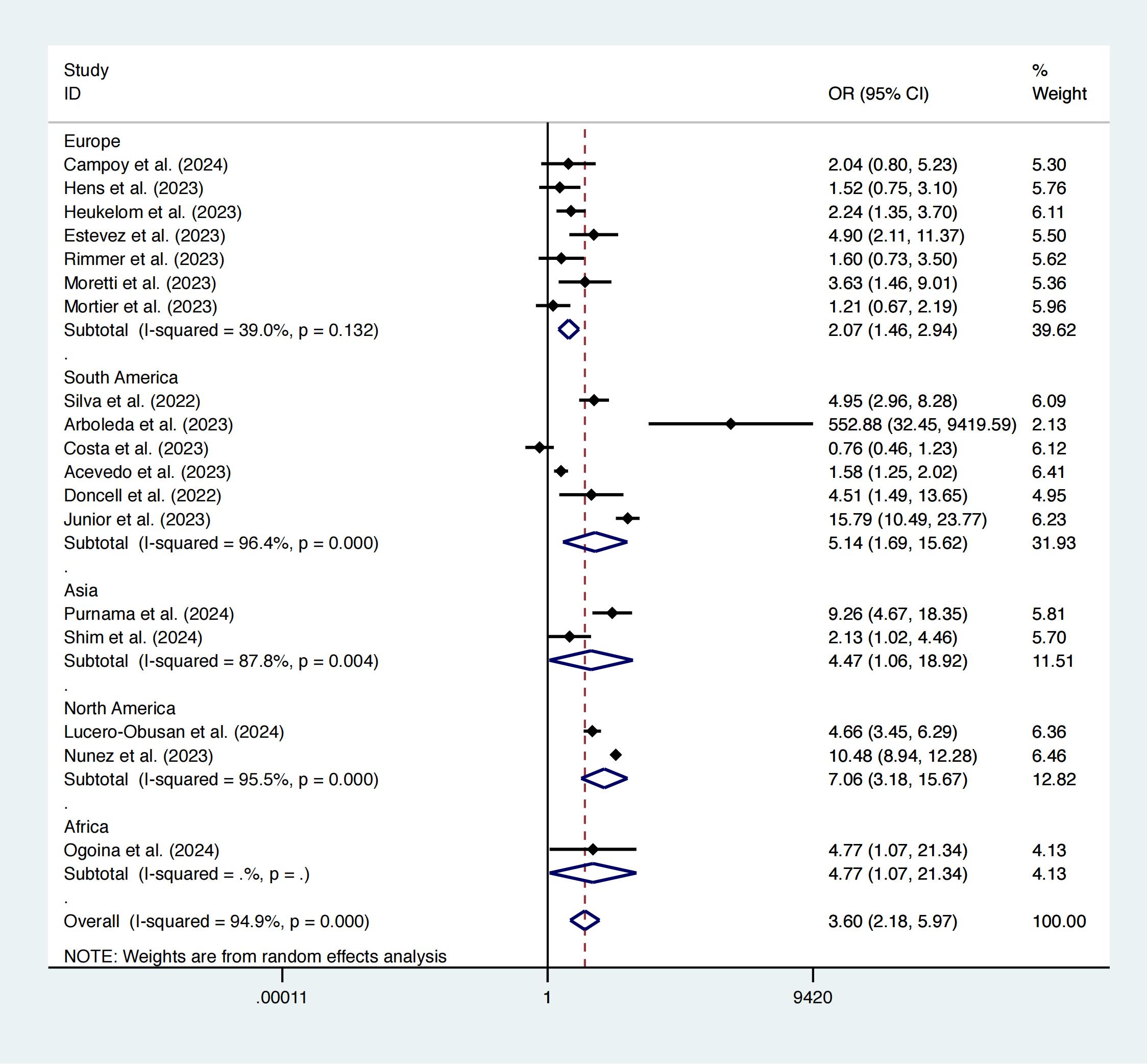


**Figure S17:** Forest plot of differences in sexually transmitted infections between mpox patients and non-mpox patients: human immunodeficiency virus.


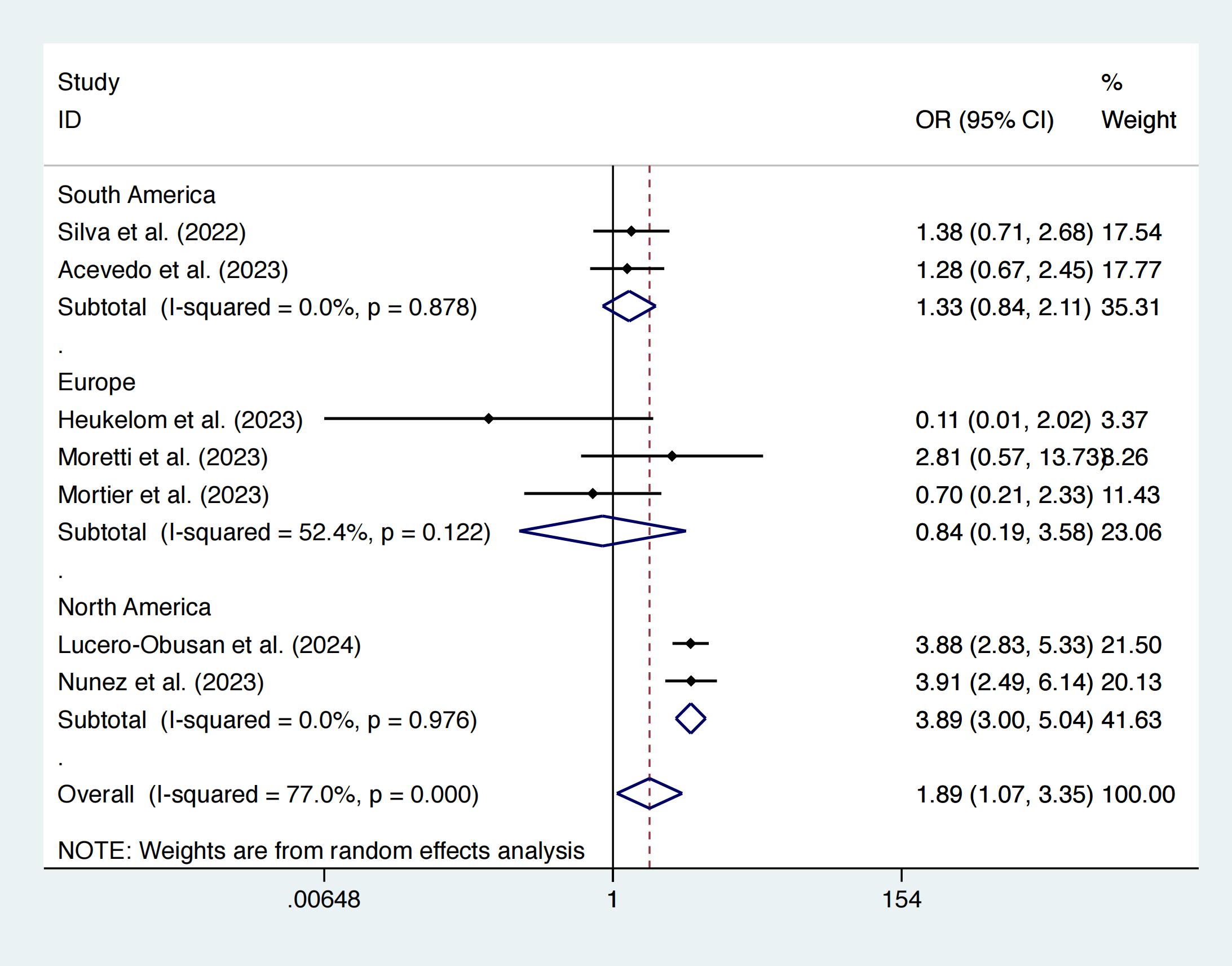


**Figure S18:** Forest plot of differences in sexually transmitted infections between mpox patients and non-mpox patients: syphilis.


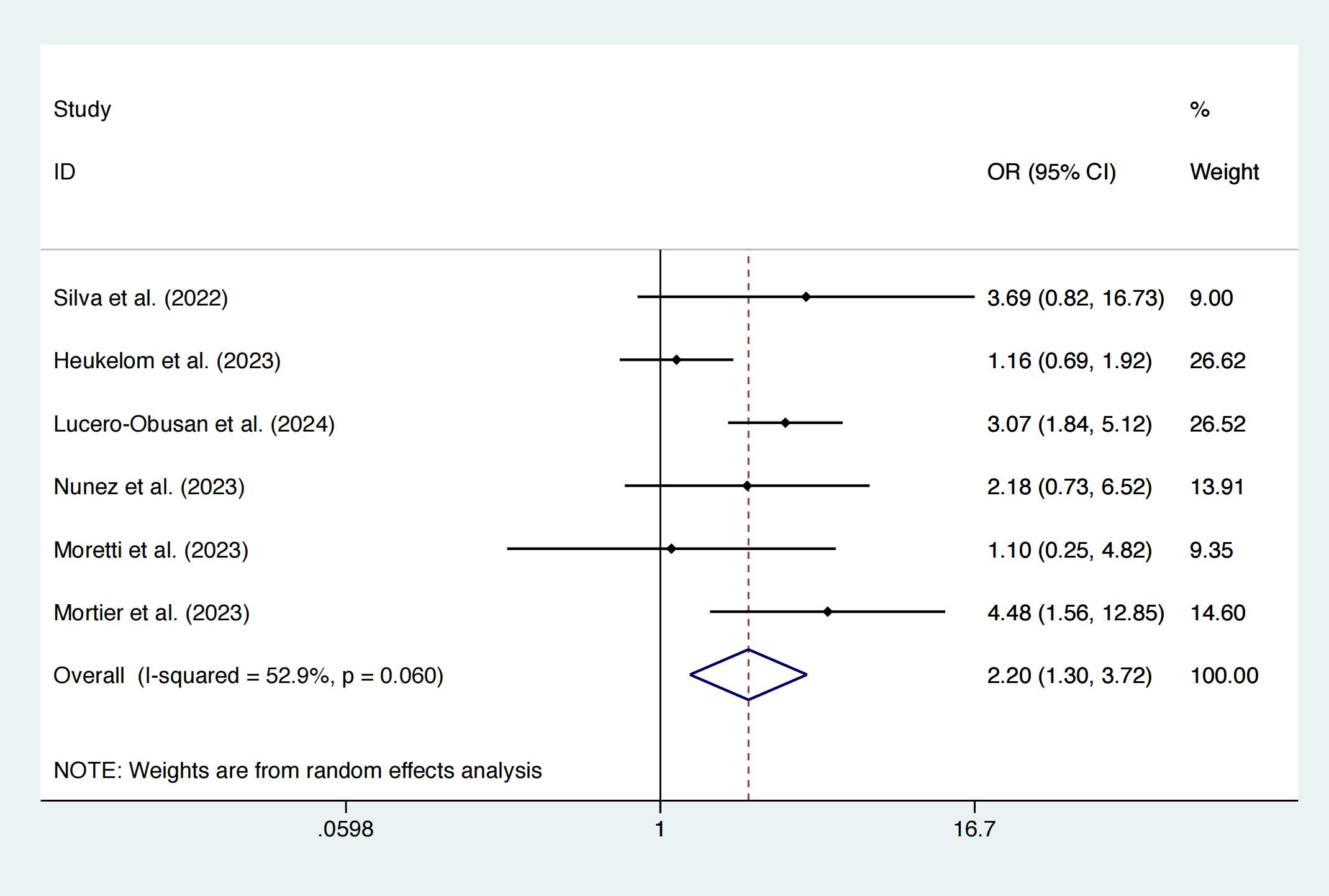


**Figure S19:** Forest plot of differences in sexually transmitted infections between mpox patients and non-mpox patients: gonorrhea.


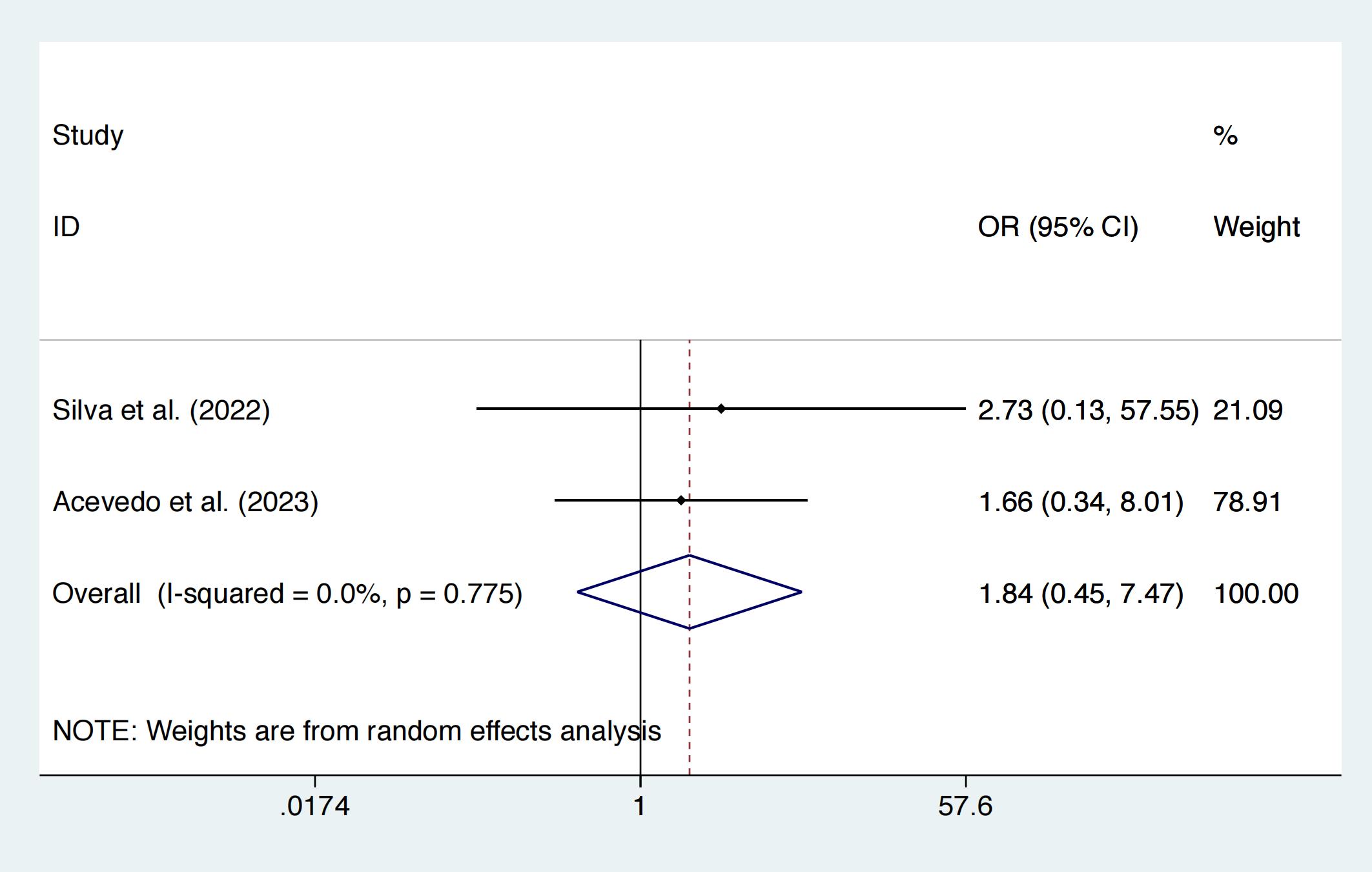


**Figure S20:** Forest plot of differences in sexually transmitted infections between mpox patients and non-mpox patients: hepatitis B virus.


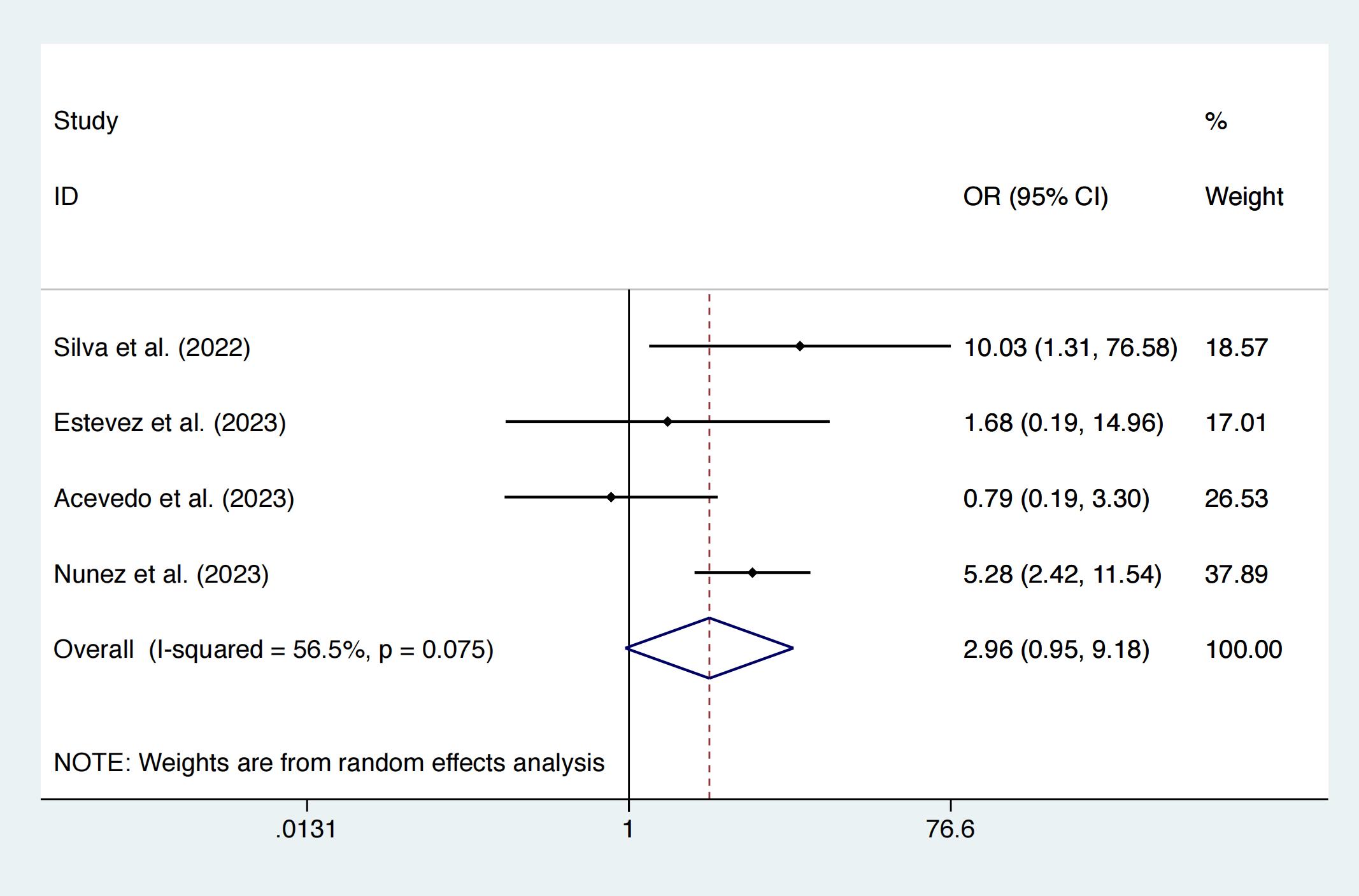


**Figure S21:** Forest plot of differences in sexually transmitted infections between mpox patients and non-mpox patients: hepatitis C virus.


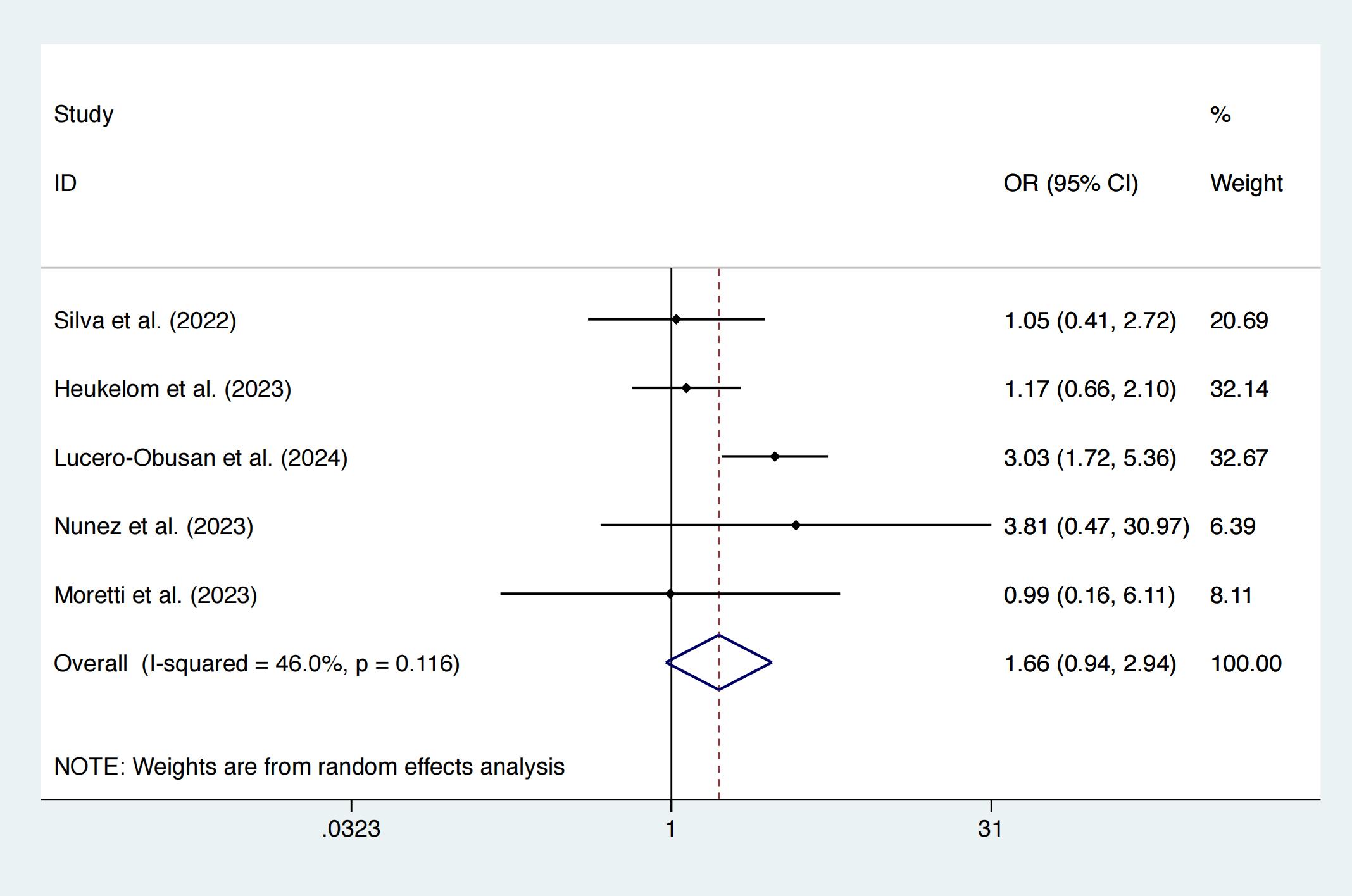


**Figure S22:** Forest plot of differences in sexually transmitted infections between mpox patients and non-mpox patients: chlamydia.


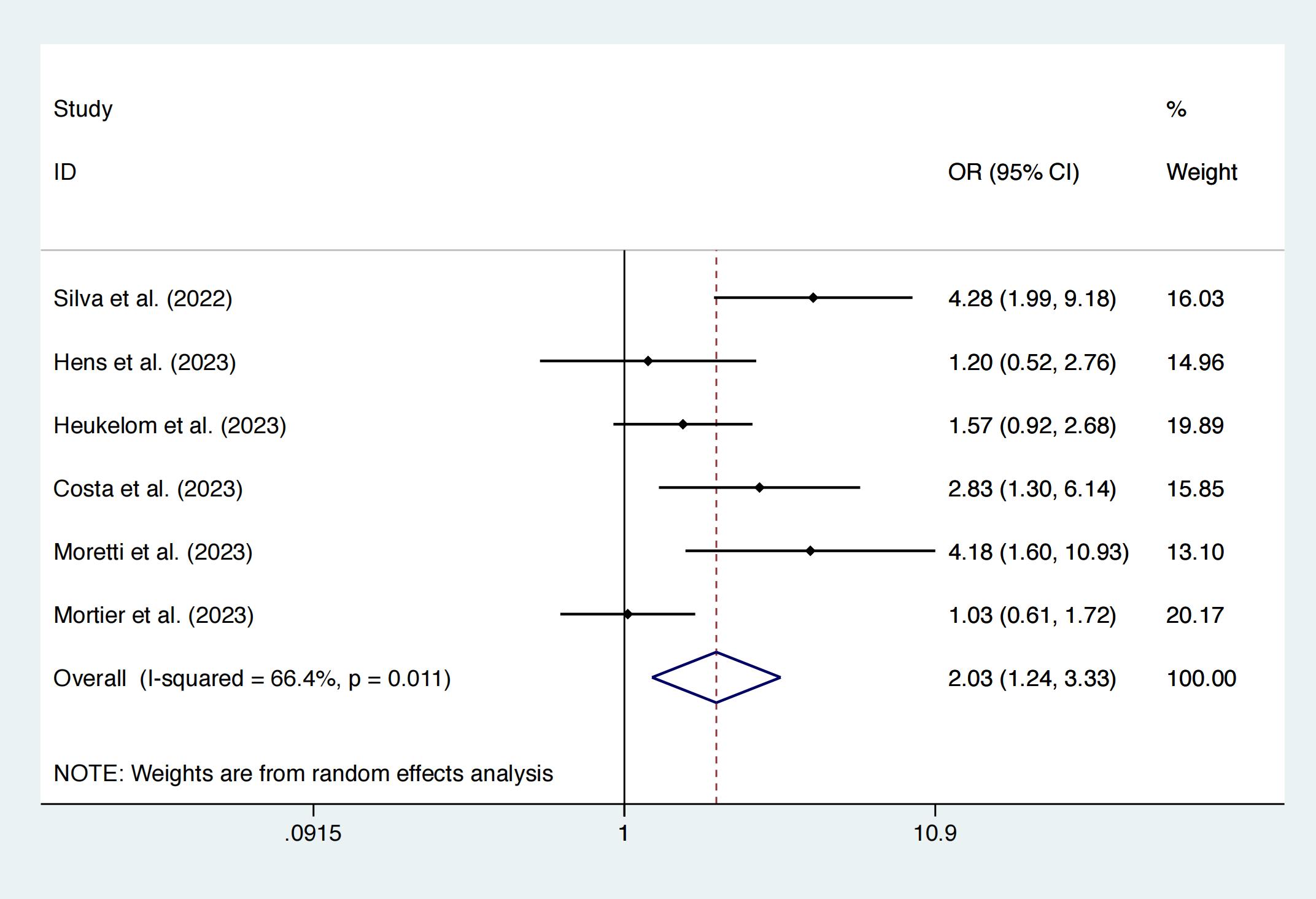


**Figure S23:** Forest plot of differences in sexually transmitted infections between mpox patients and non-mpox patients: pre-exposure prophylaxis.


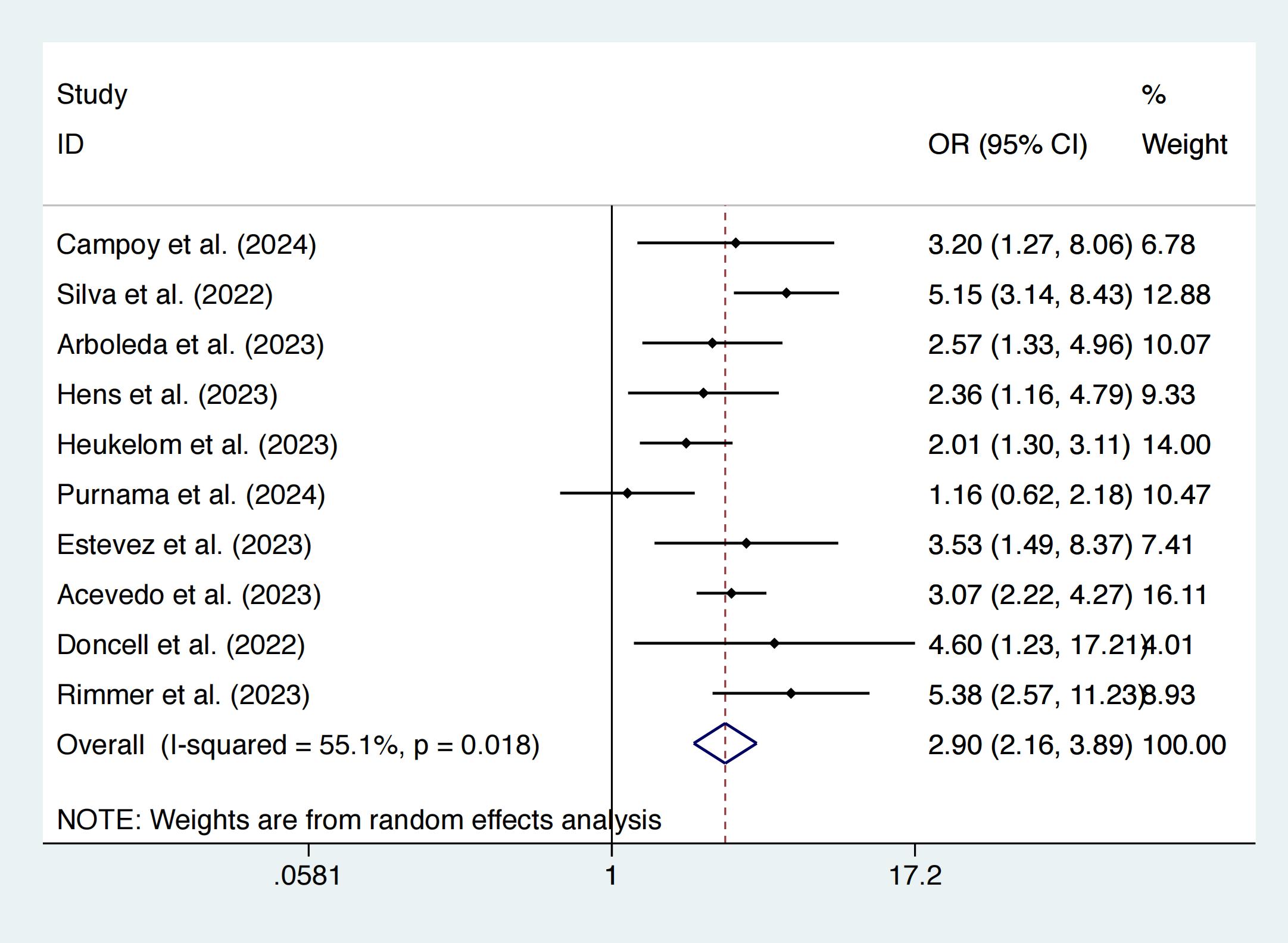


**Figure S24:** Forest plot of differences in the locations of lesions between mpox patients and non-mpox patients: genitals.


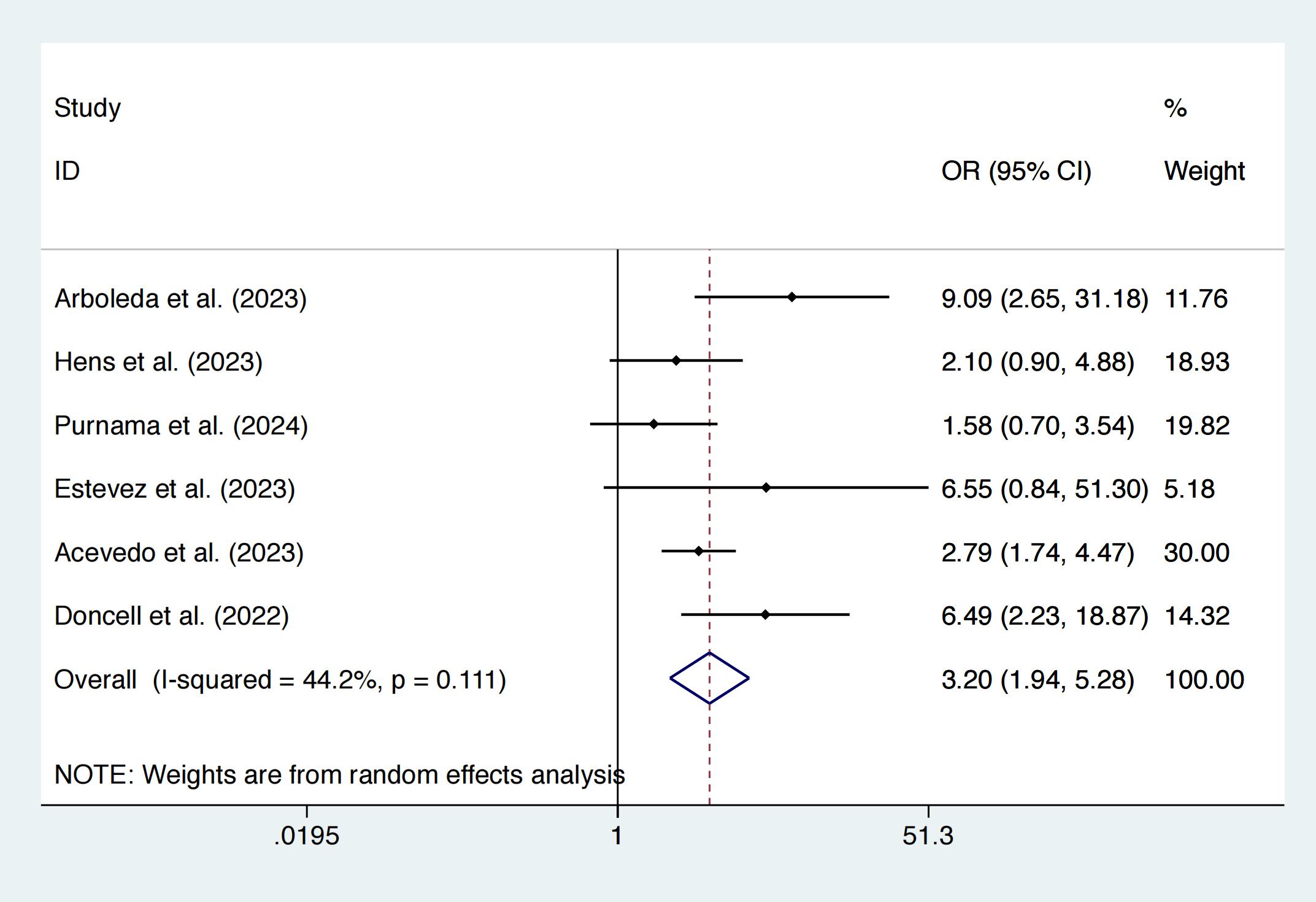


**Figure S25:** Forest plot of differences in the locations of lesions between mpox patients and non-mpox patients: perianal region.


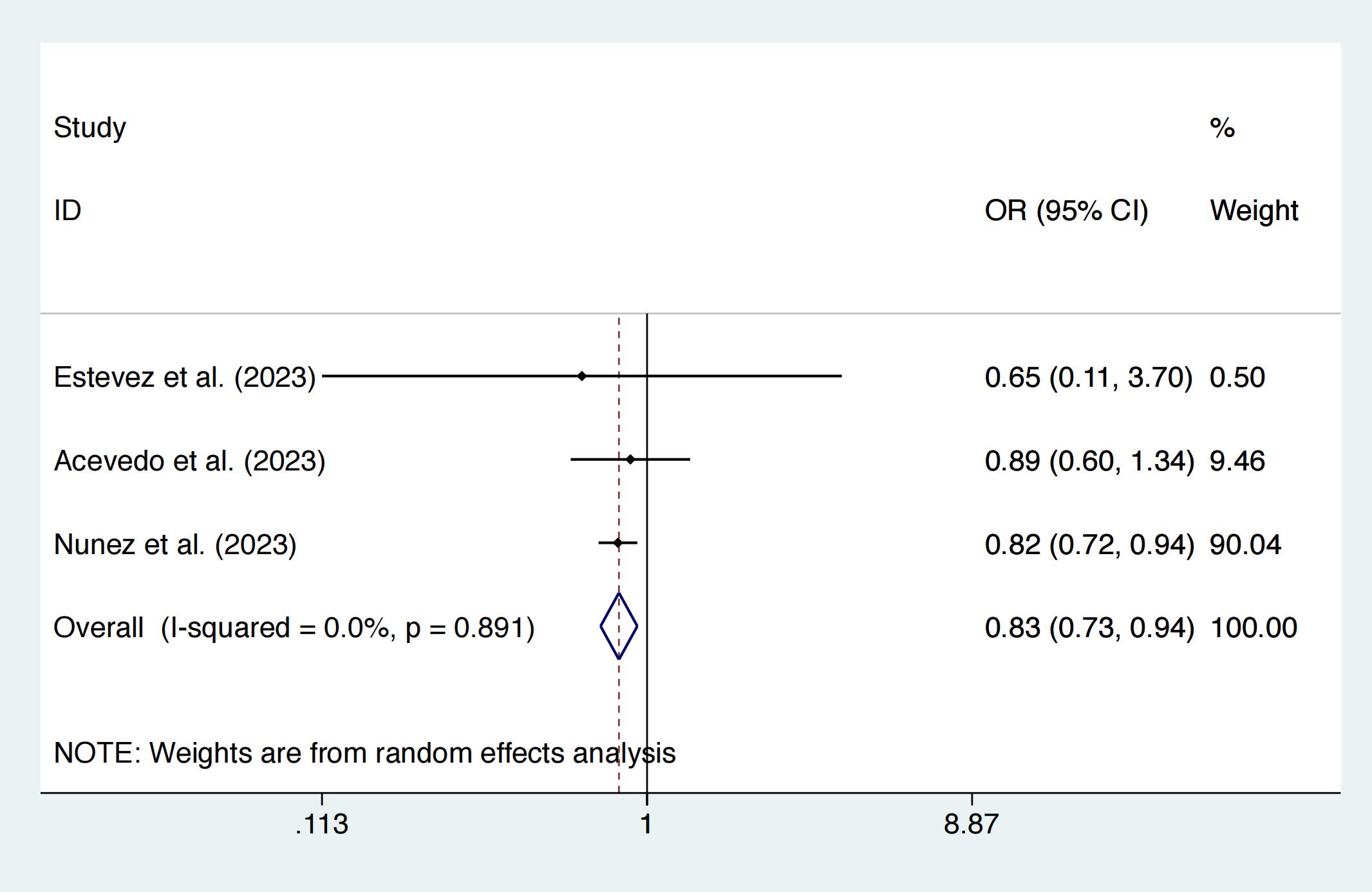


**Figure S26:** Forest plot of differences in the locations of lesions between mpox patients and non-mpox patients: palms.


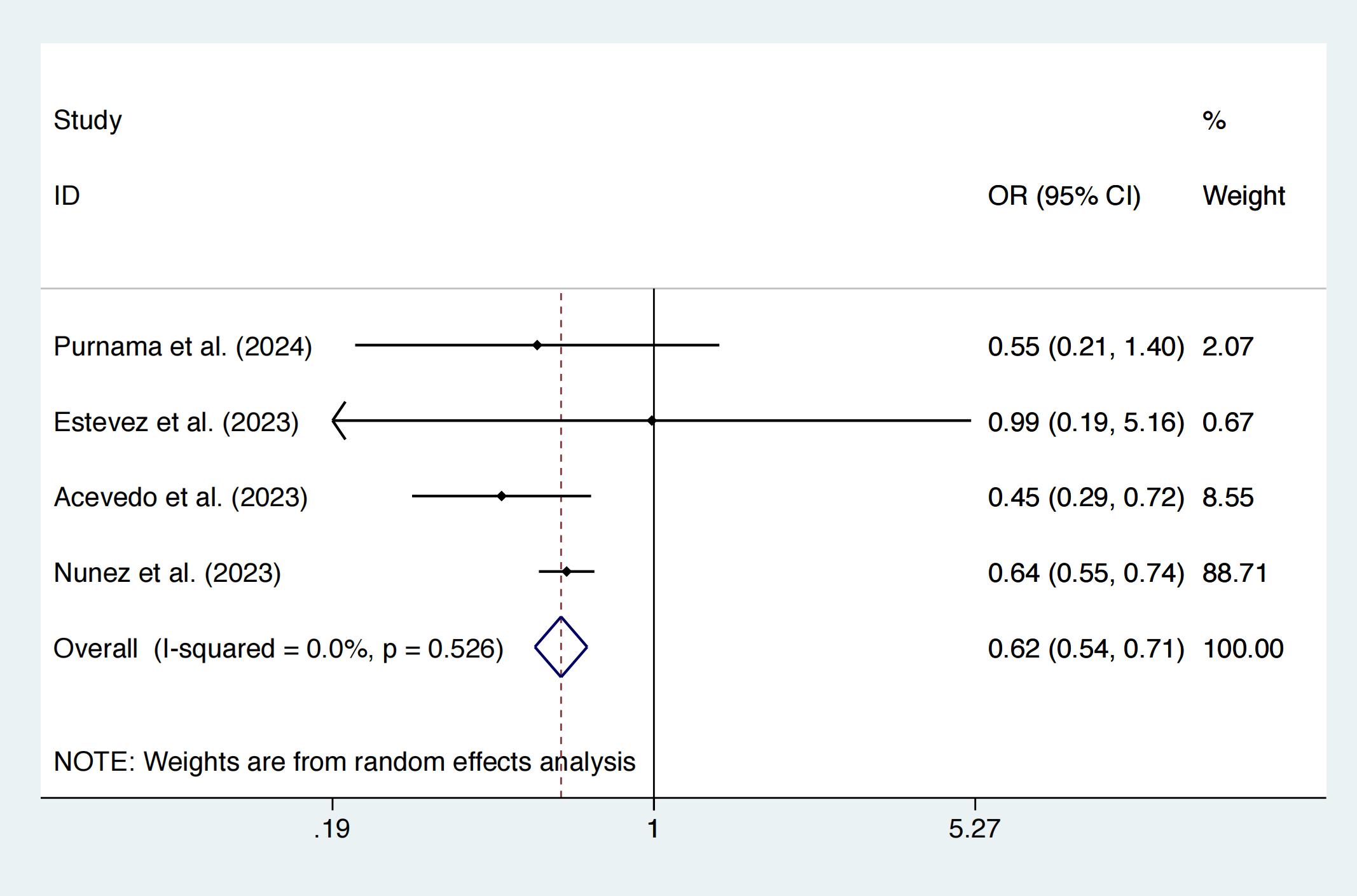


**Figure S27:** Forest plot of differences in the locations of lesions between mpox patients and non-mpox patients: soles.


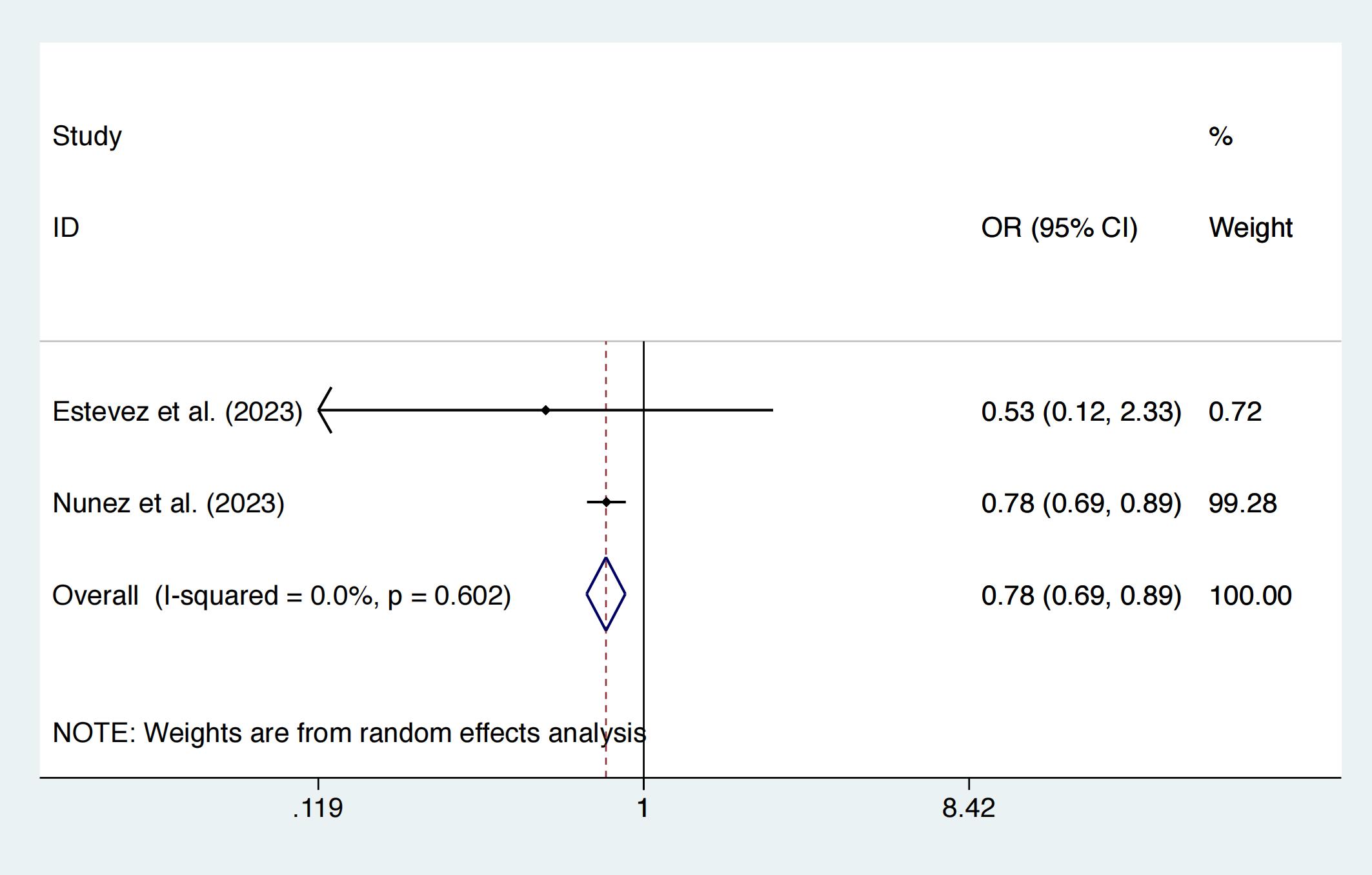


**Figure S28:** Forest plot of differences in the locations of lesions between mpox patients and non-mpox patients: neck.


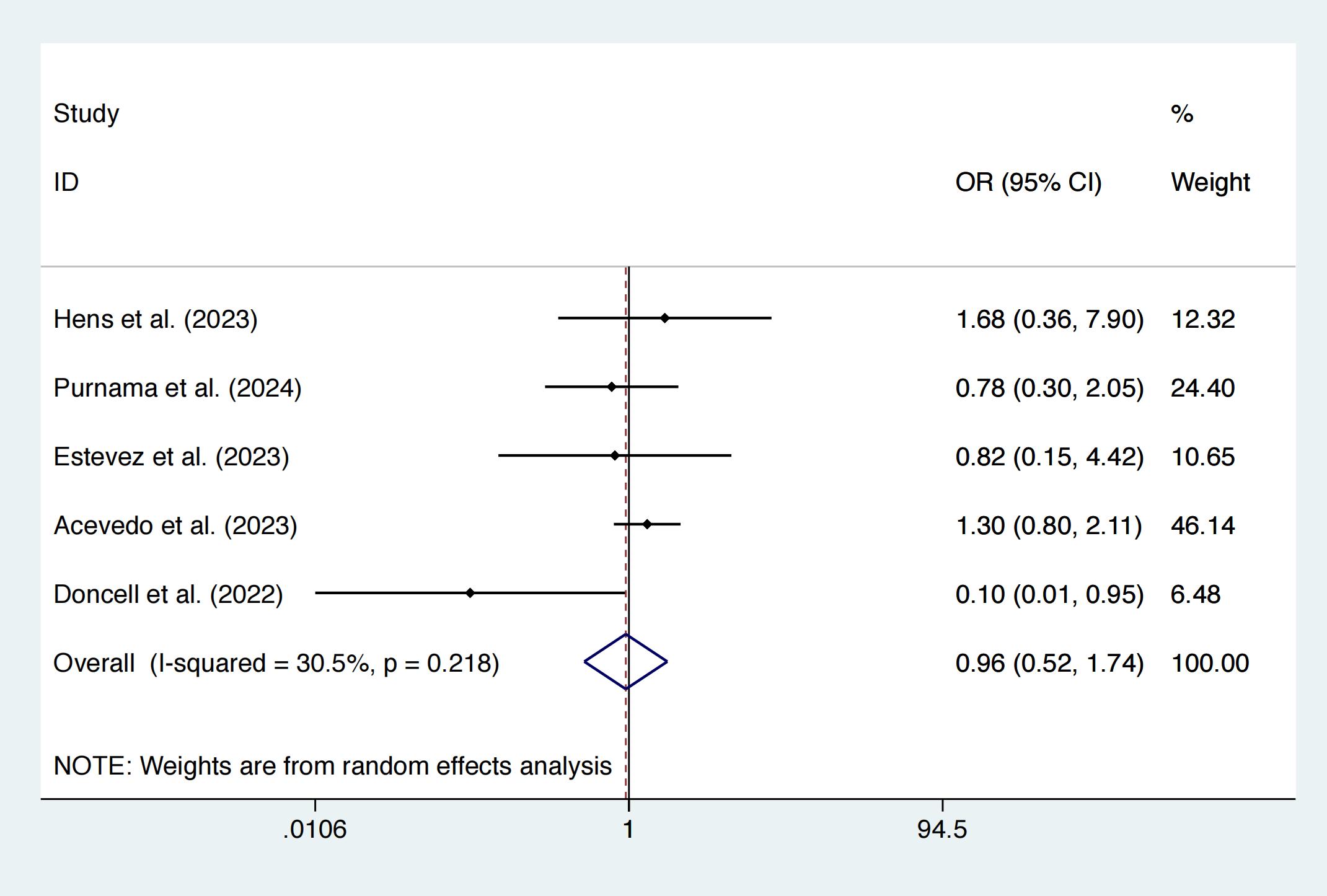


**Figure S29:** Forest plot of differences in the locations of lesions between mpox patients and non-mpox patients: oral cavity.


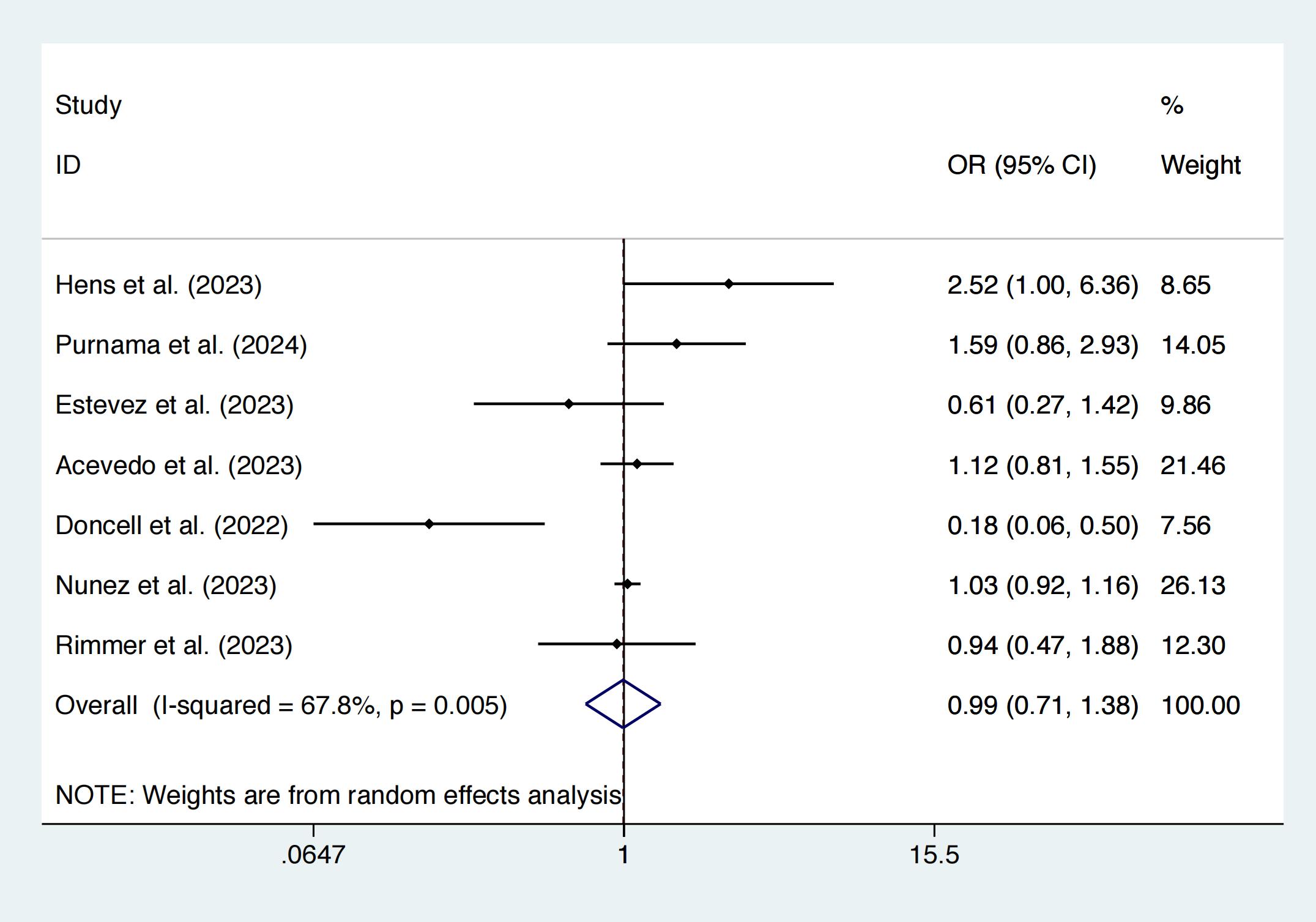


**Figure S30:** Forest plot of differences in the locations of lesions between mpox patients and non-mpox patients: face.


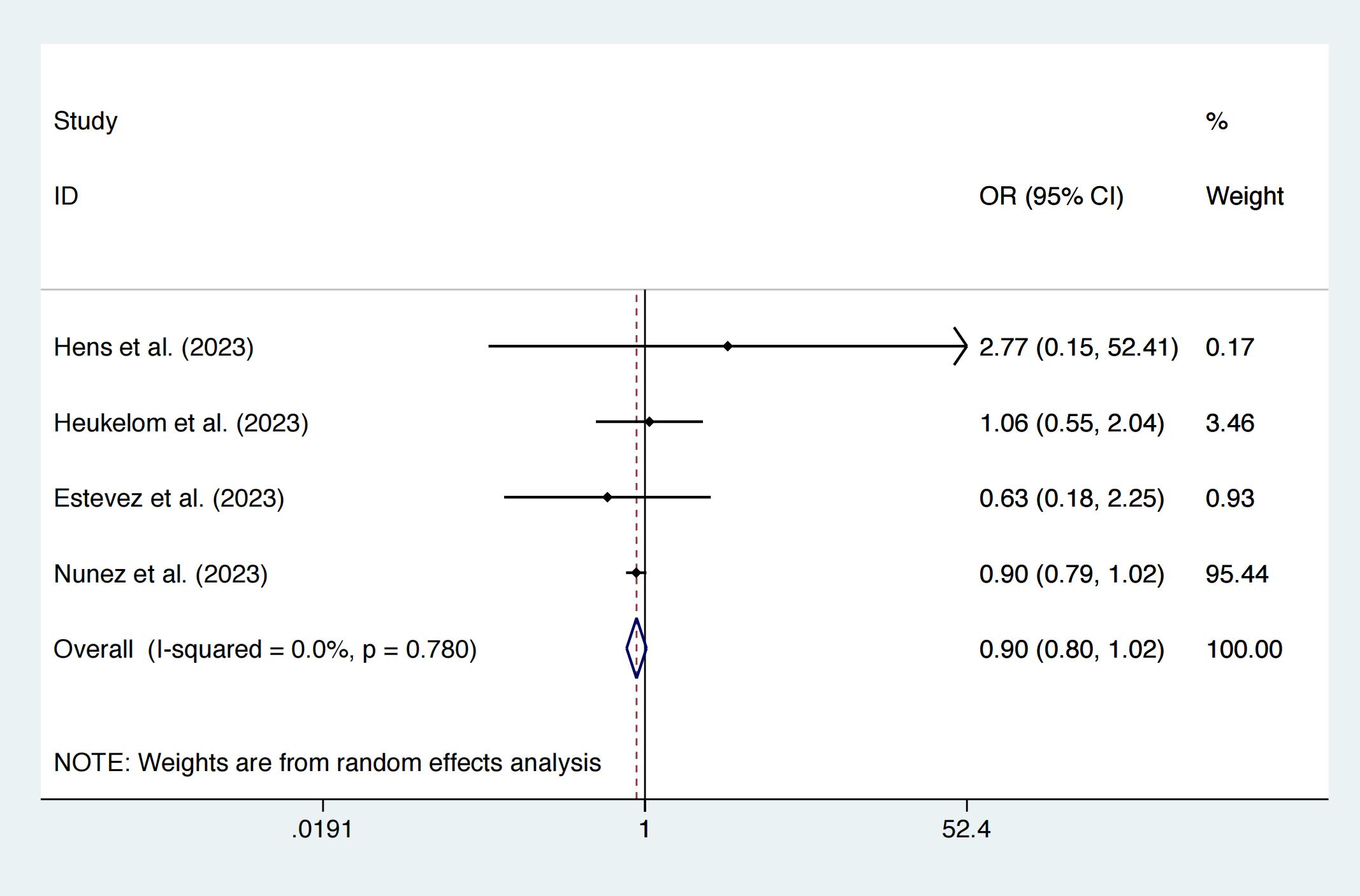


**Figure S31:** Forest plot of differences in the locations of lesions between mpox patients and non-mpox patients: head.


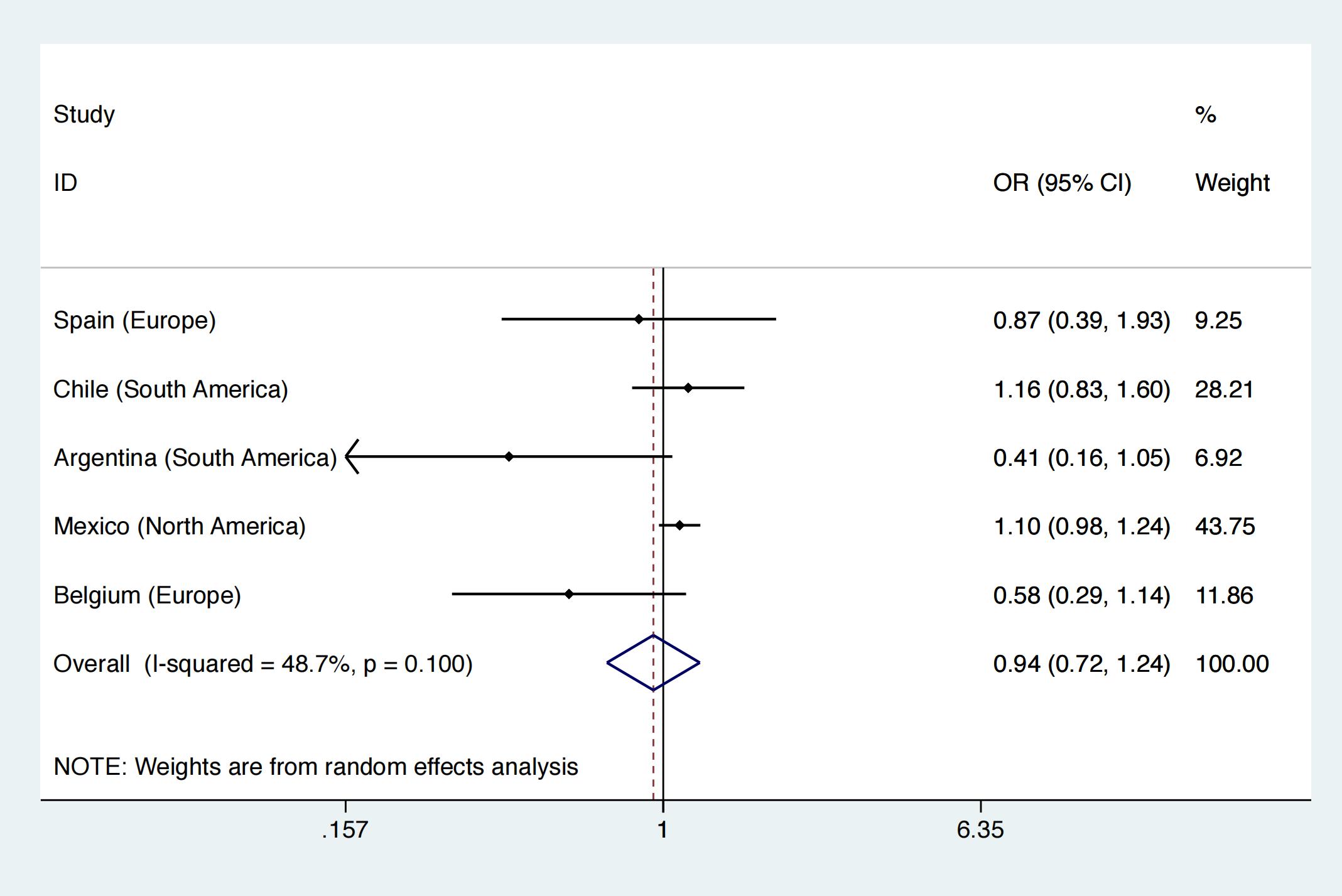


**Figure S32:** Forest plot of differences in the locations of lesions between mpox patients and non-mpox patients: arms.


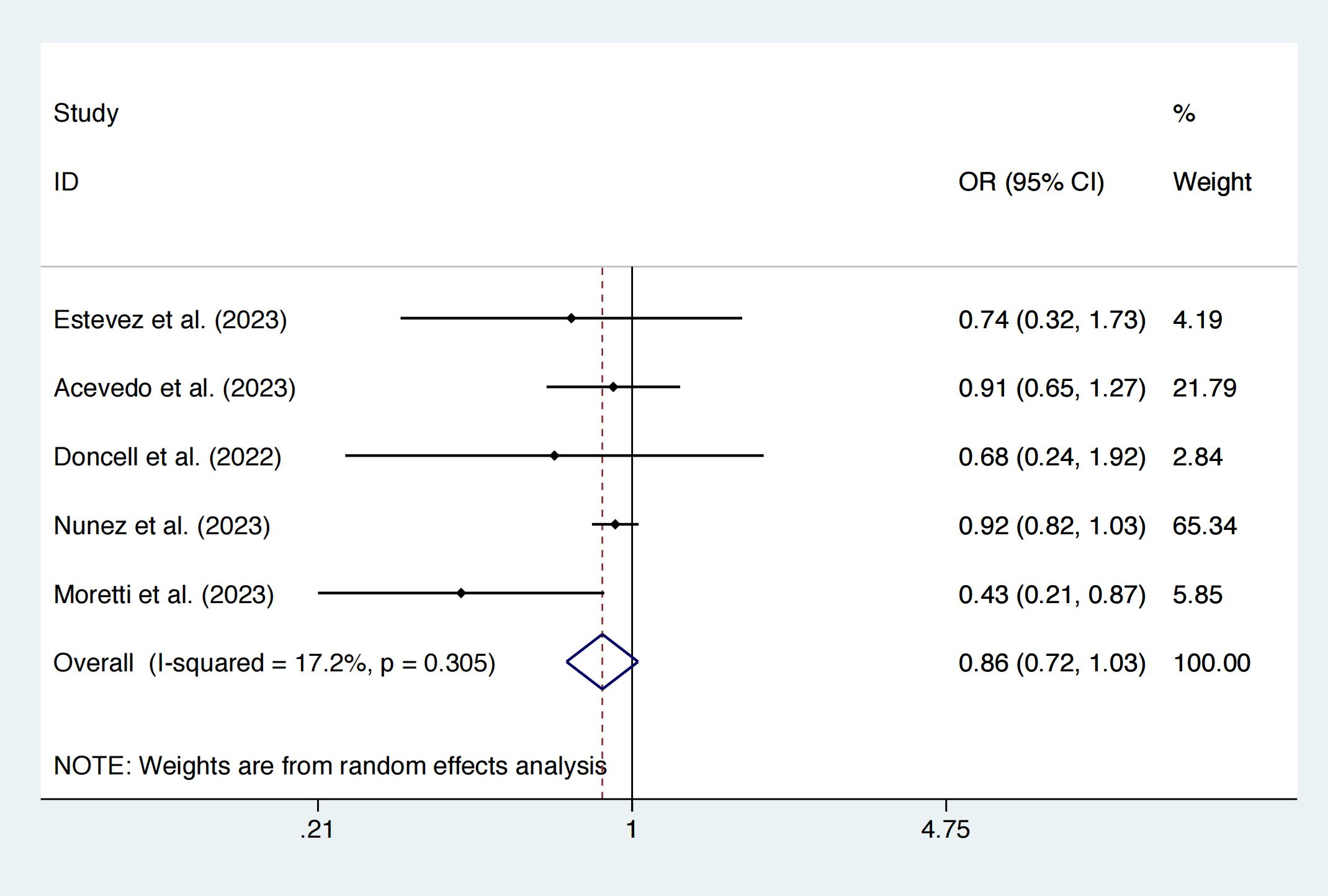


**Figure S33:** Forest plot of differences in the locations of lesions between mpox patients and non-mpox patients: legs.


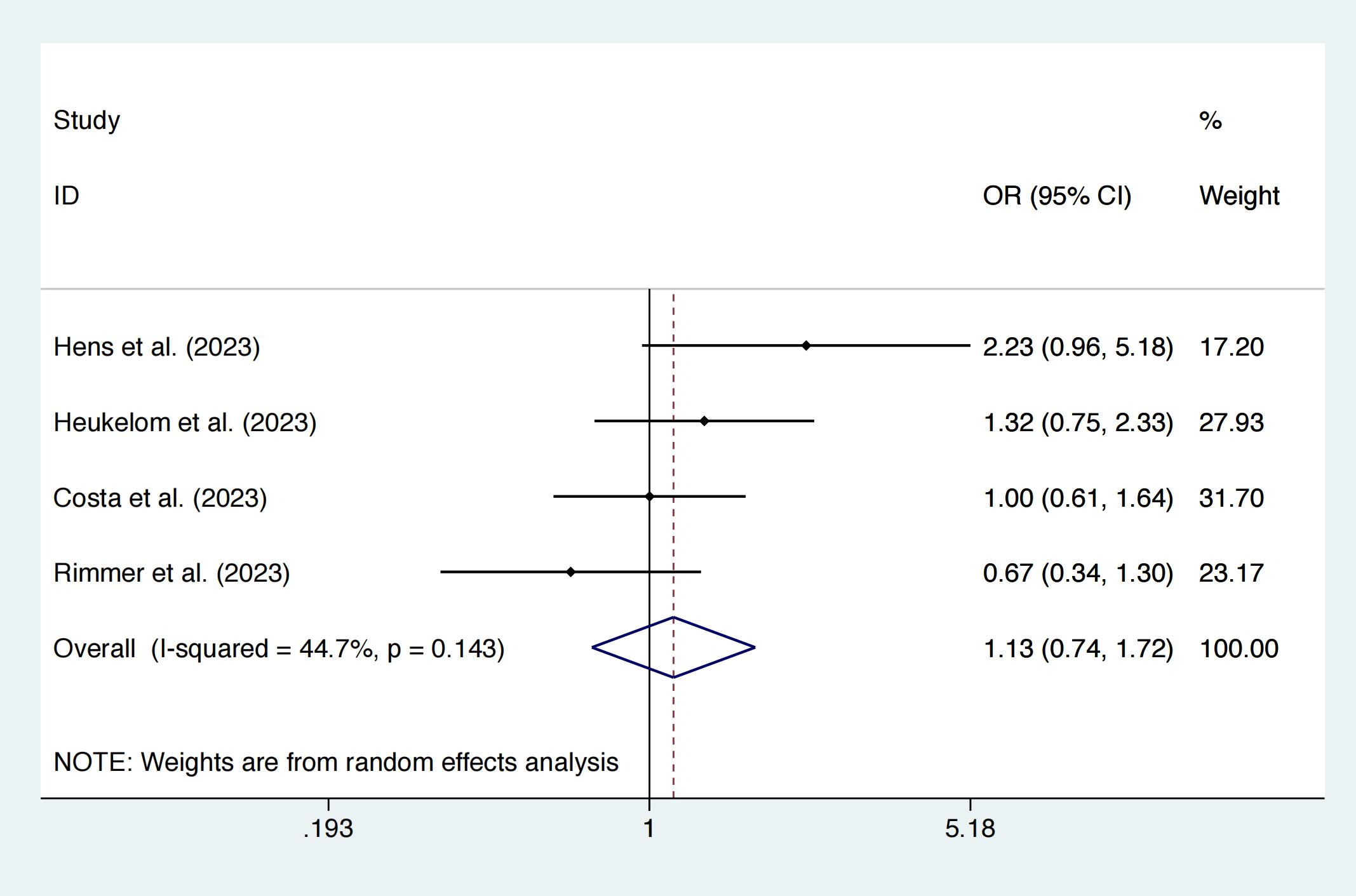


**Figure S34:** Forest plot of differences in the locations of lesions between mpox patients and non-mpox patients: trunk.


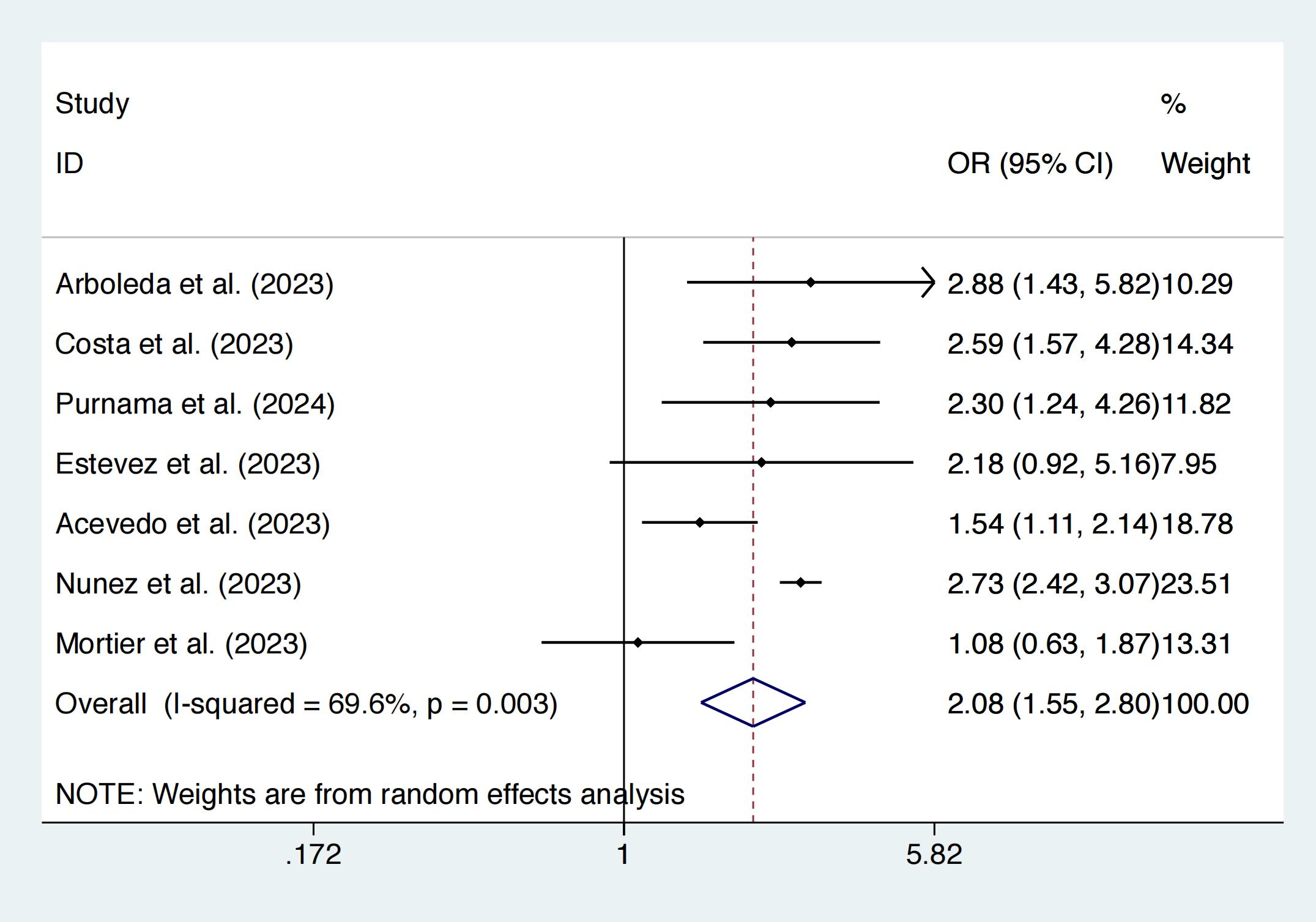


**Figure S35:** Forest plot of differences in the types of lesions between mpox patients and non-mpox patients: pustules.


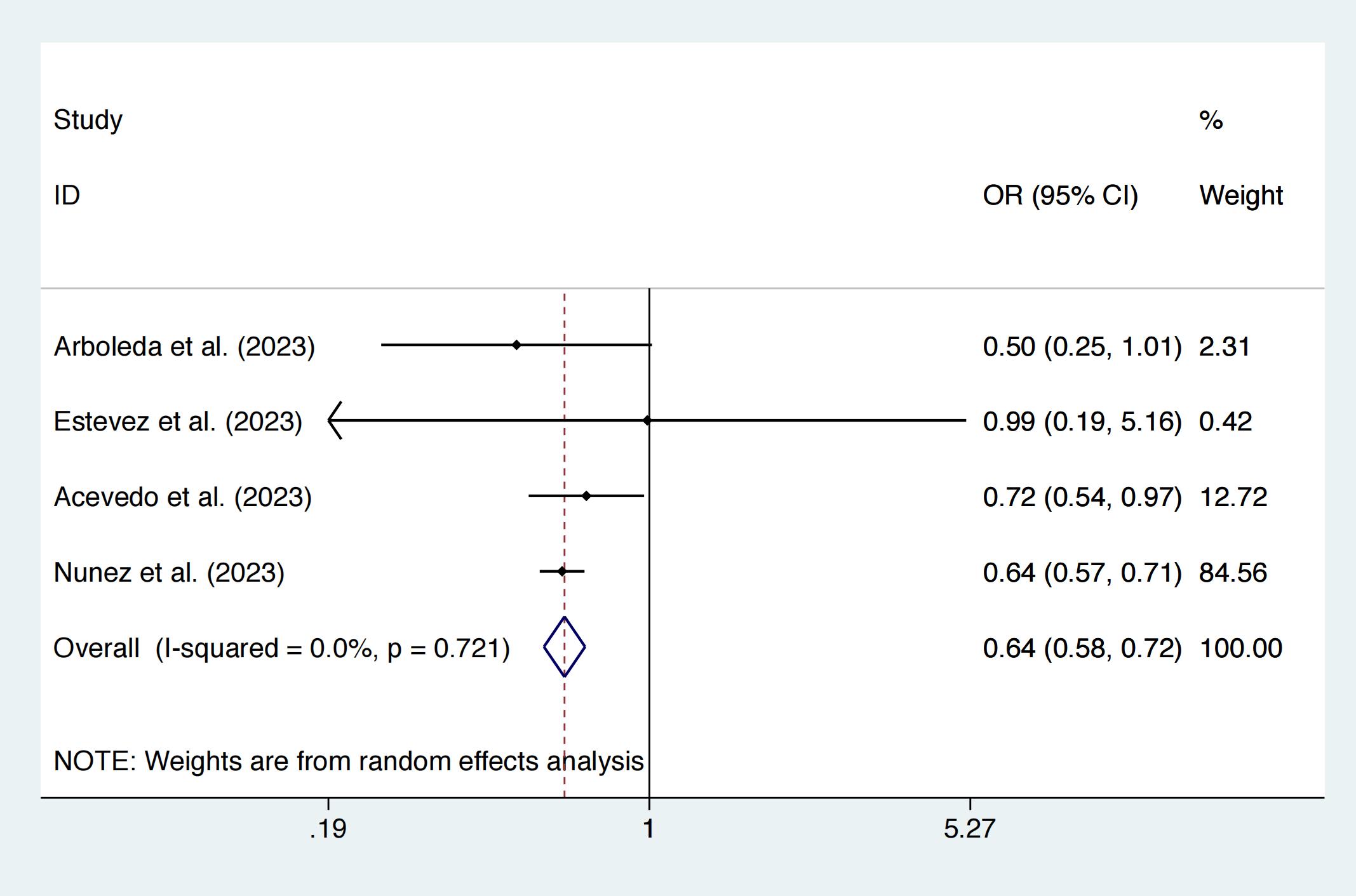


**Figure S36:** Forest plot of differences in the types of lesions between mpox patients and non-mpox patients: maculae.


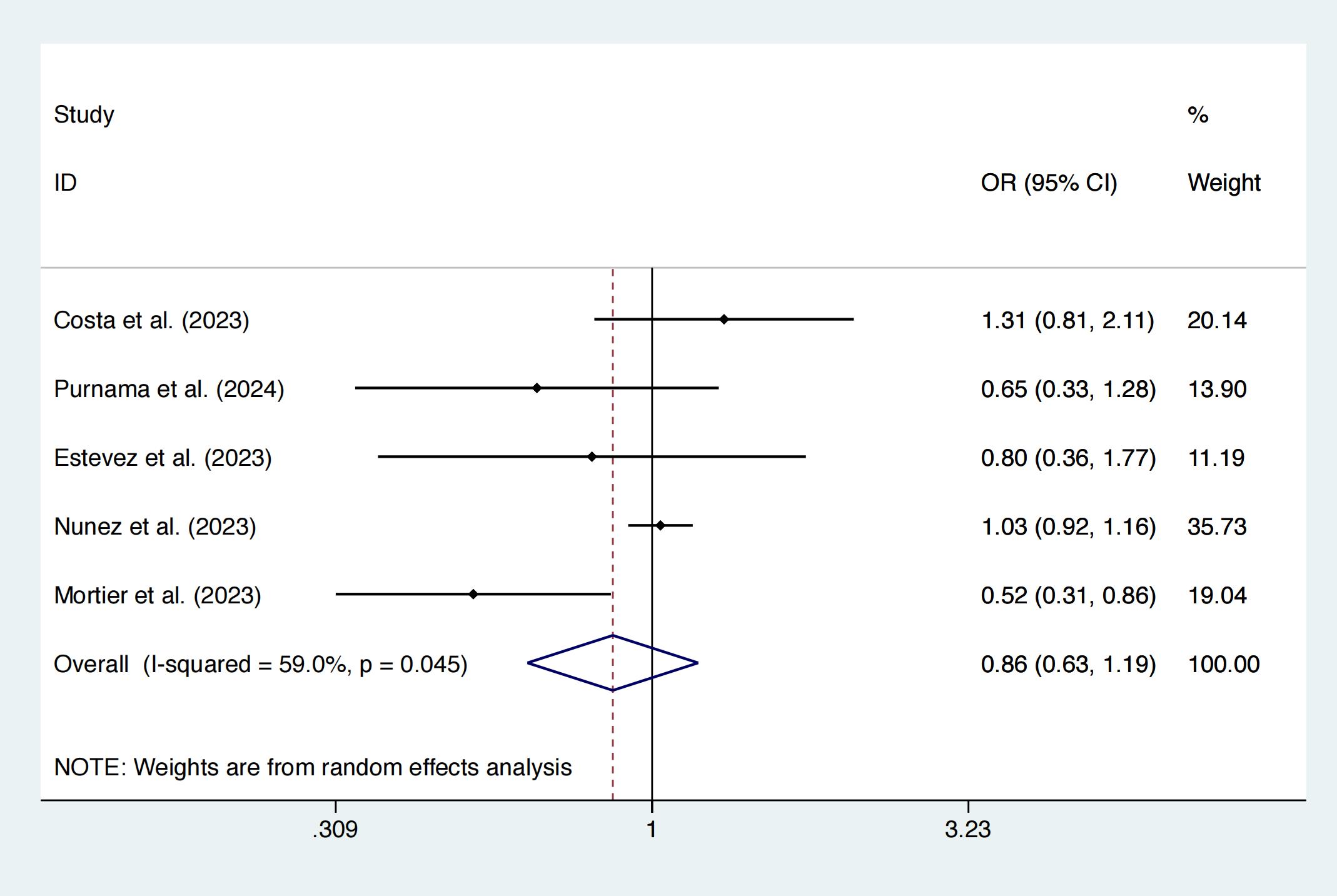


**Figure S37:** Forest plot of differences in the types of lesions between mpox patients and non-mpox patients: papulae.


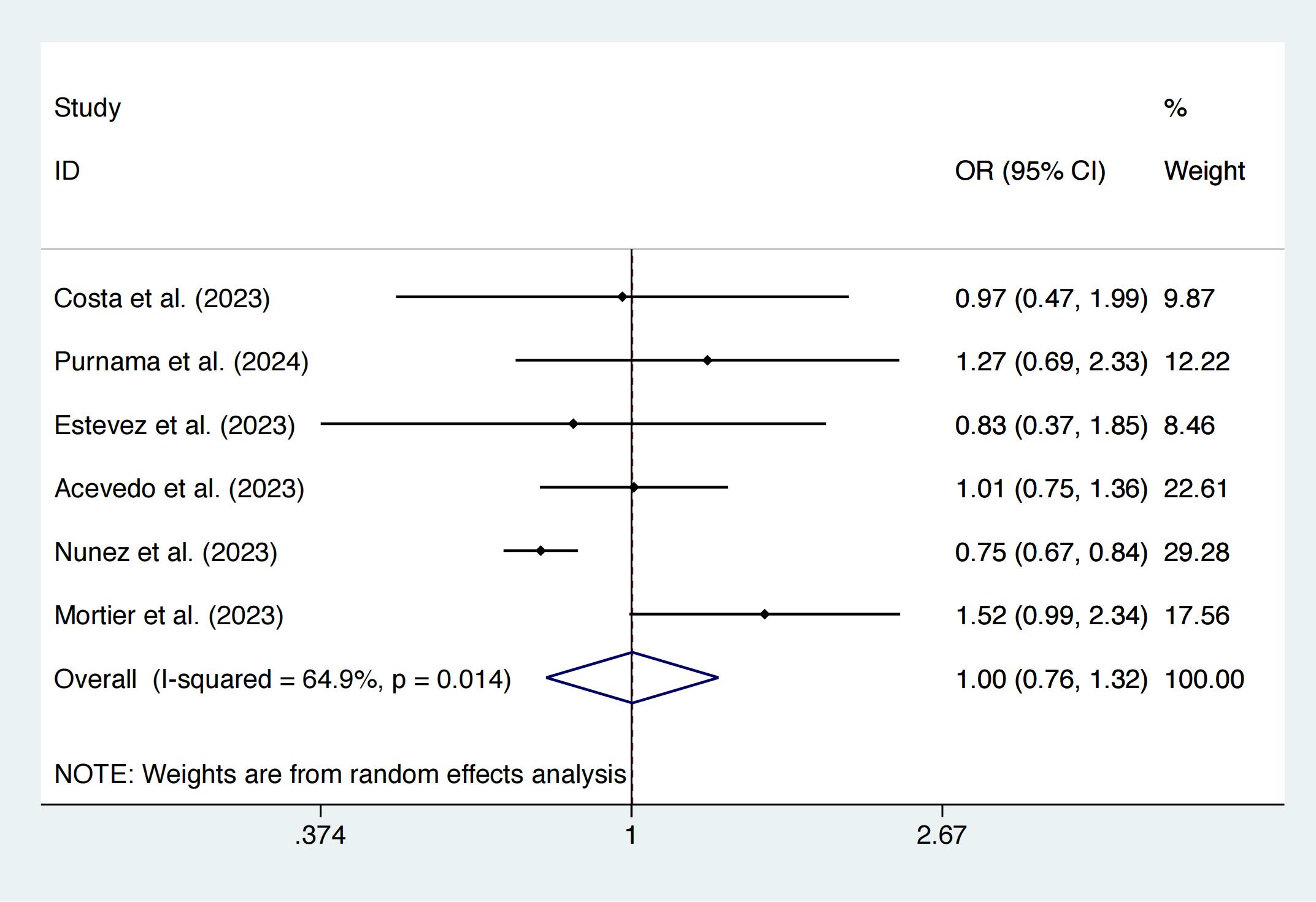


**Figure S38:** Forest plot of differences in the types of lesions between mpox patients and non-mpox patients: vesicles.


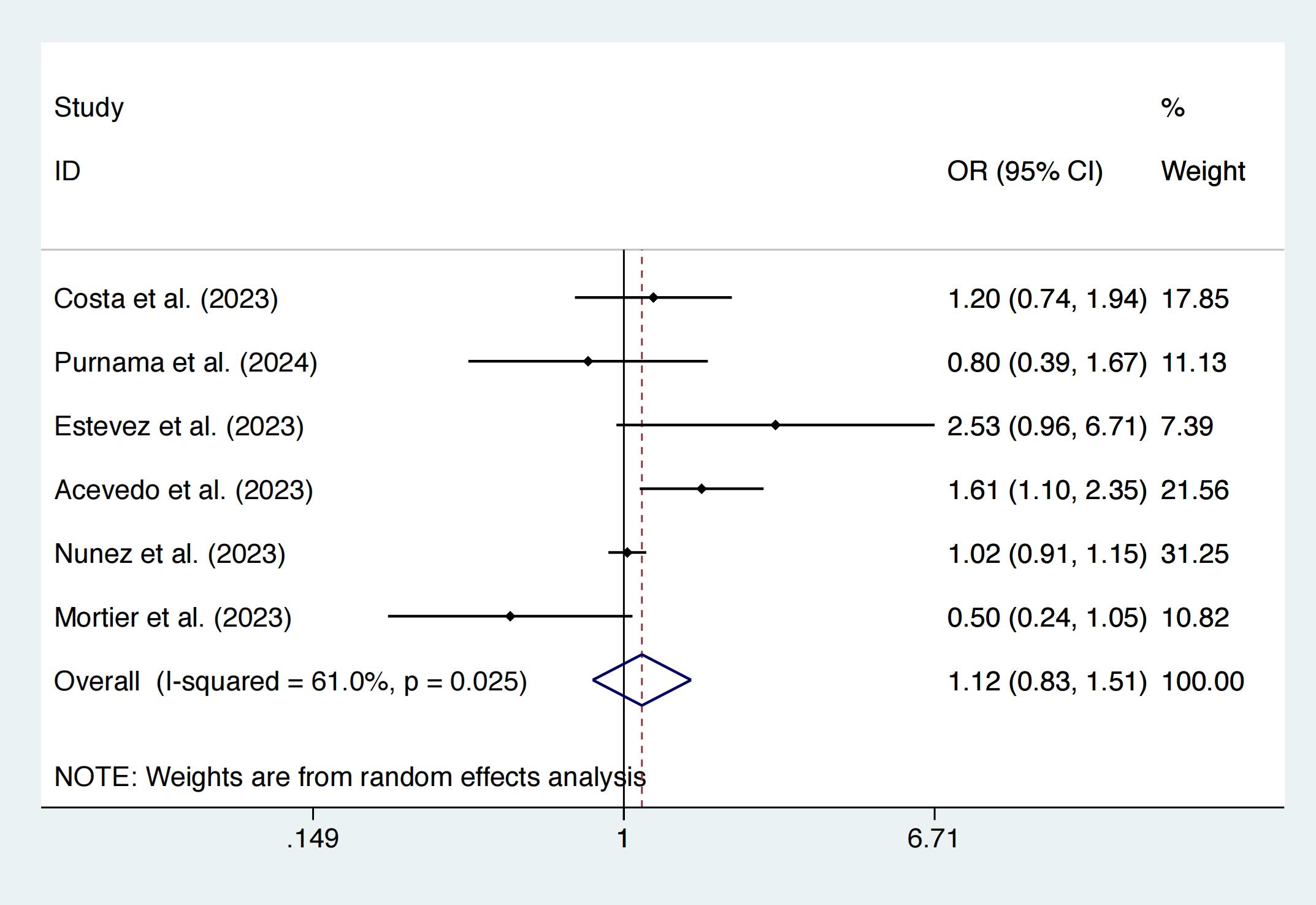


**Figure S39:** Forest plot of differences in the types of lesions between mpox patients and non-mpox patients: scabs.


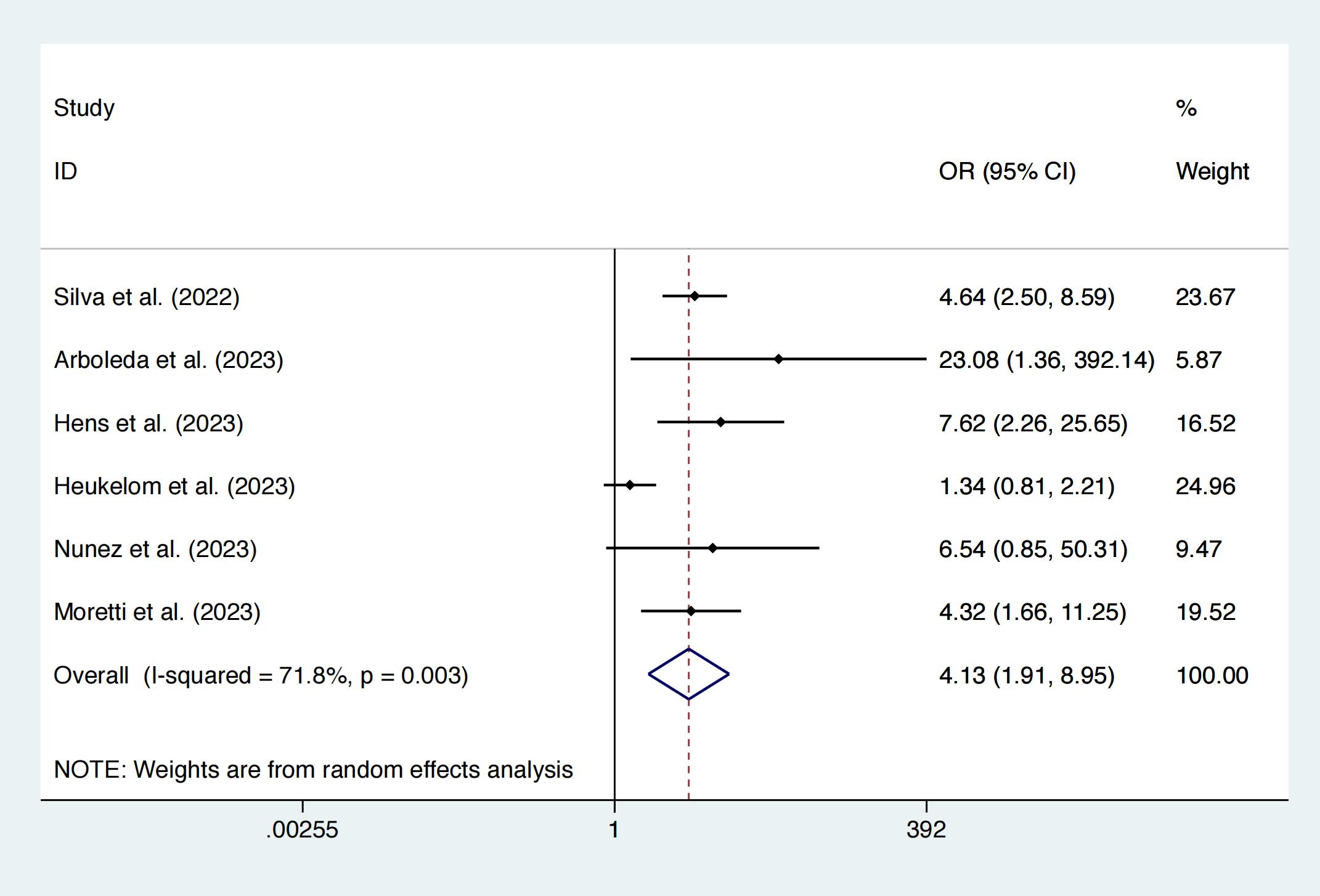


**Figure S40:** Forest plot of differences in complications between mpox patients and non-mpox patients: proctitis.


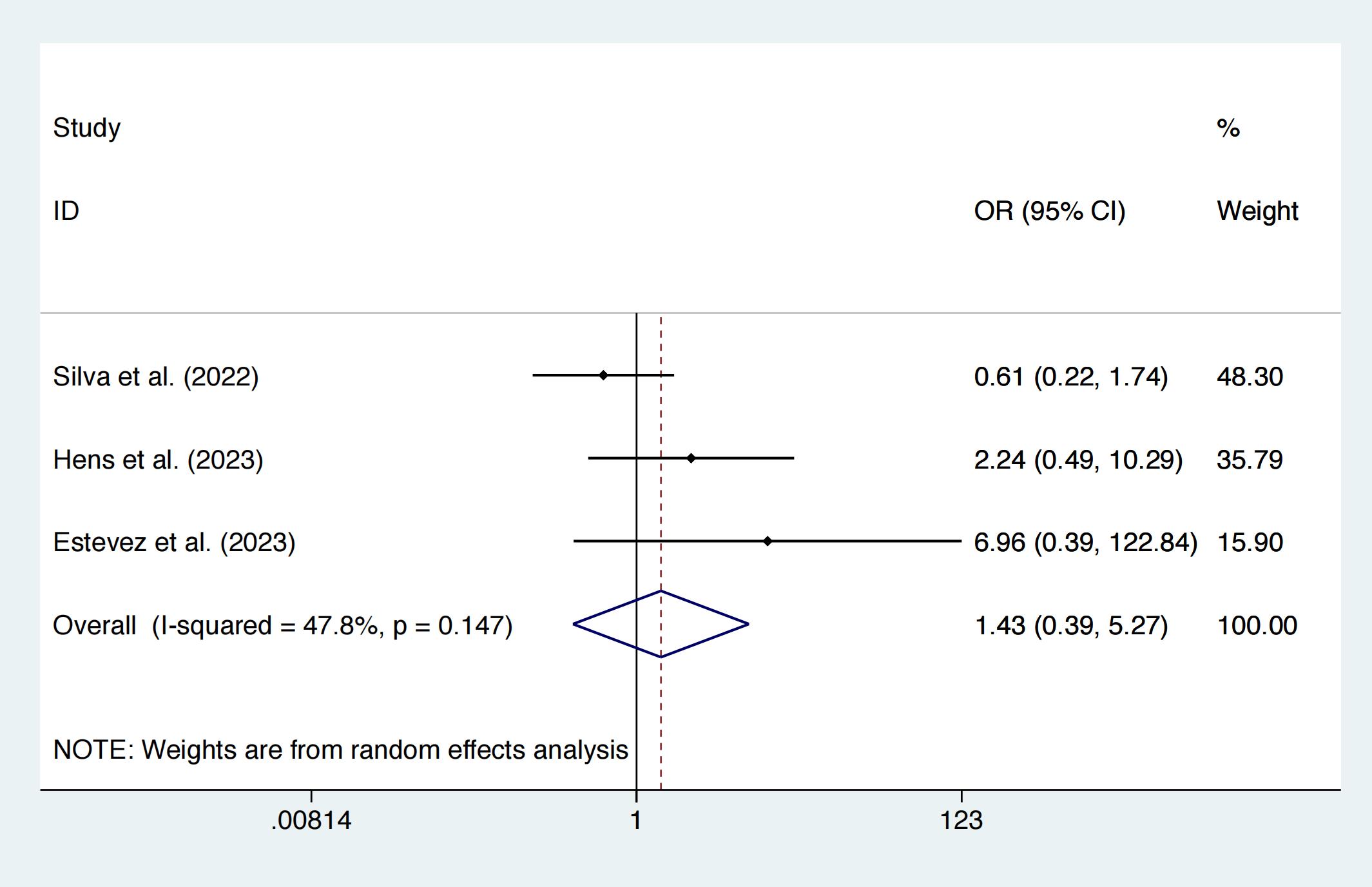


**Figure S41:** Forest plot of differences in complications between mpox patients and non-mpox patients: bacterial infection.


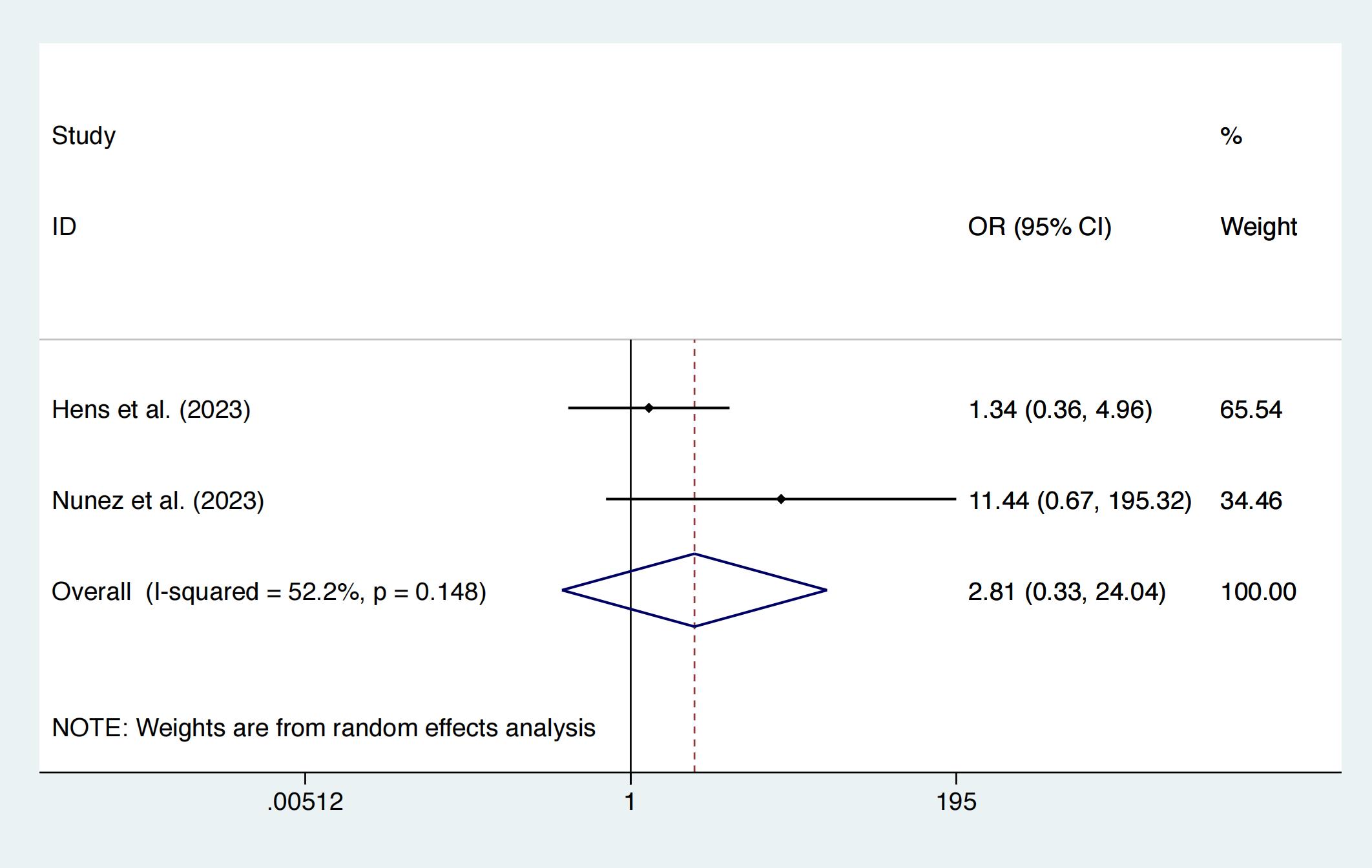


**Figure S42:** Forest plot of differences in complications between mpox patients and non-mpox patients: urethritis.


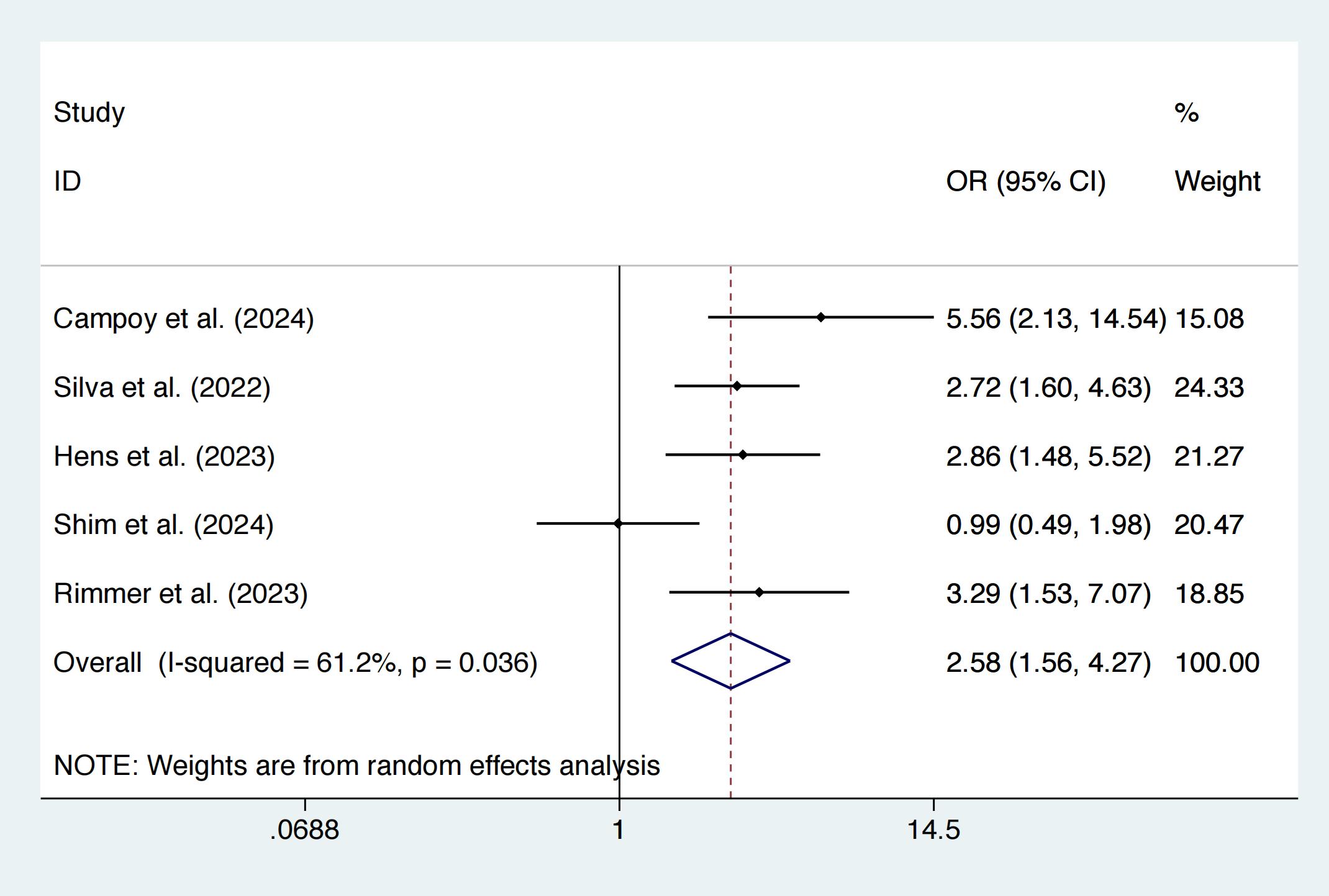


**Figure S43:** Forest plot of differences in symptoms between mpox patients and non-mpox patients: systemic symptoms.


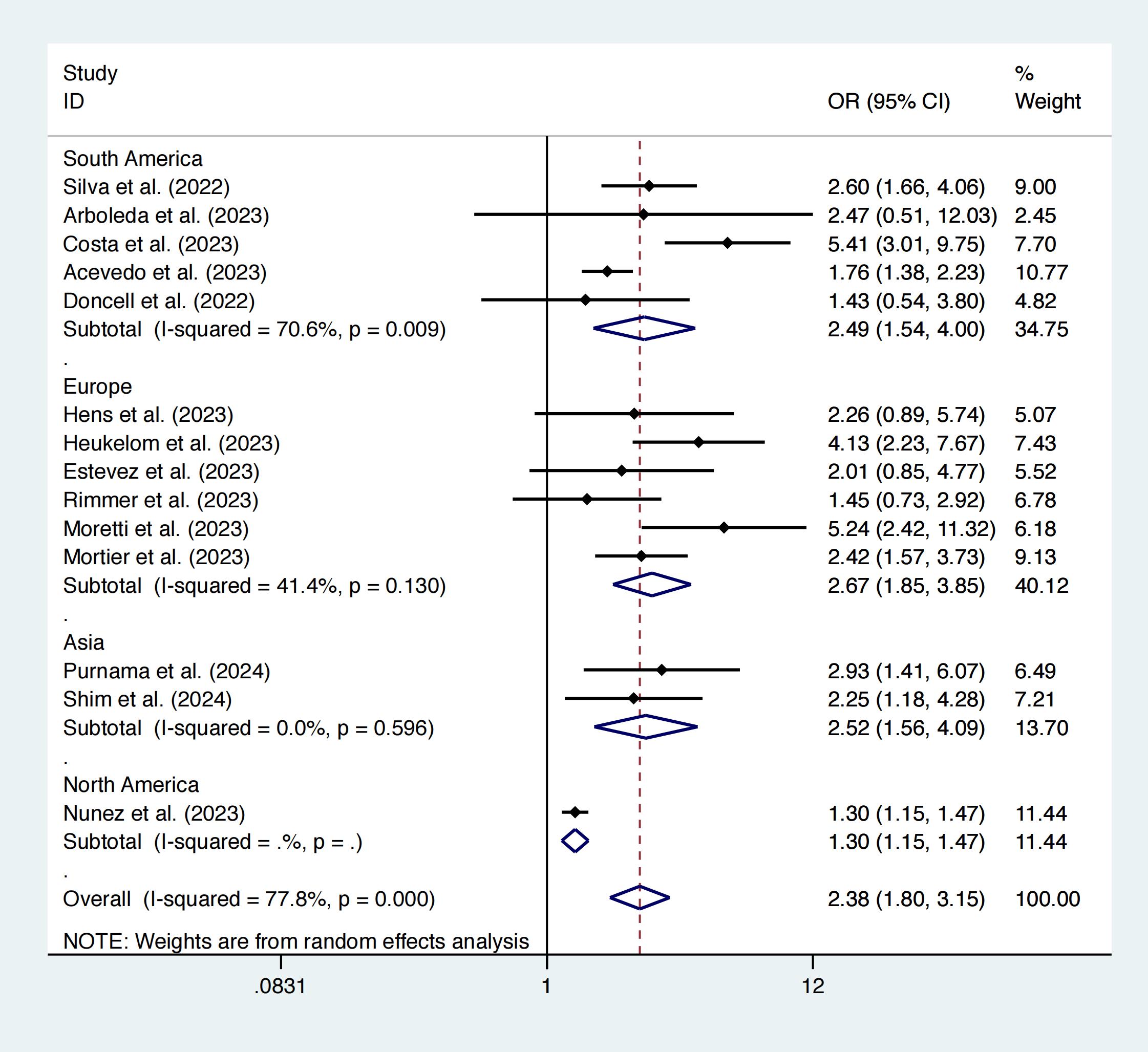


**Figure S44:** Forest plot of differences in symptoms between mpox patients and non-mpox patients: fever.


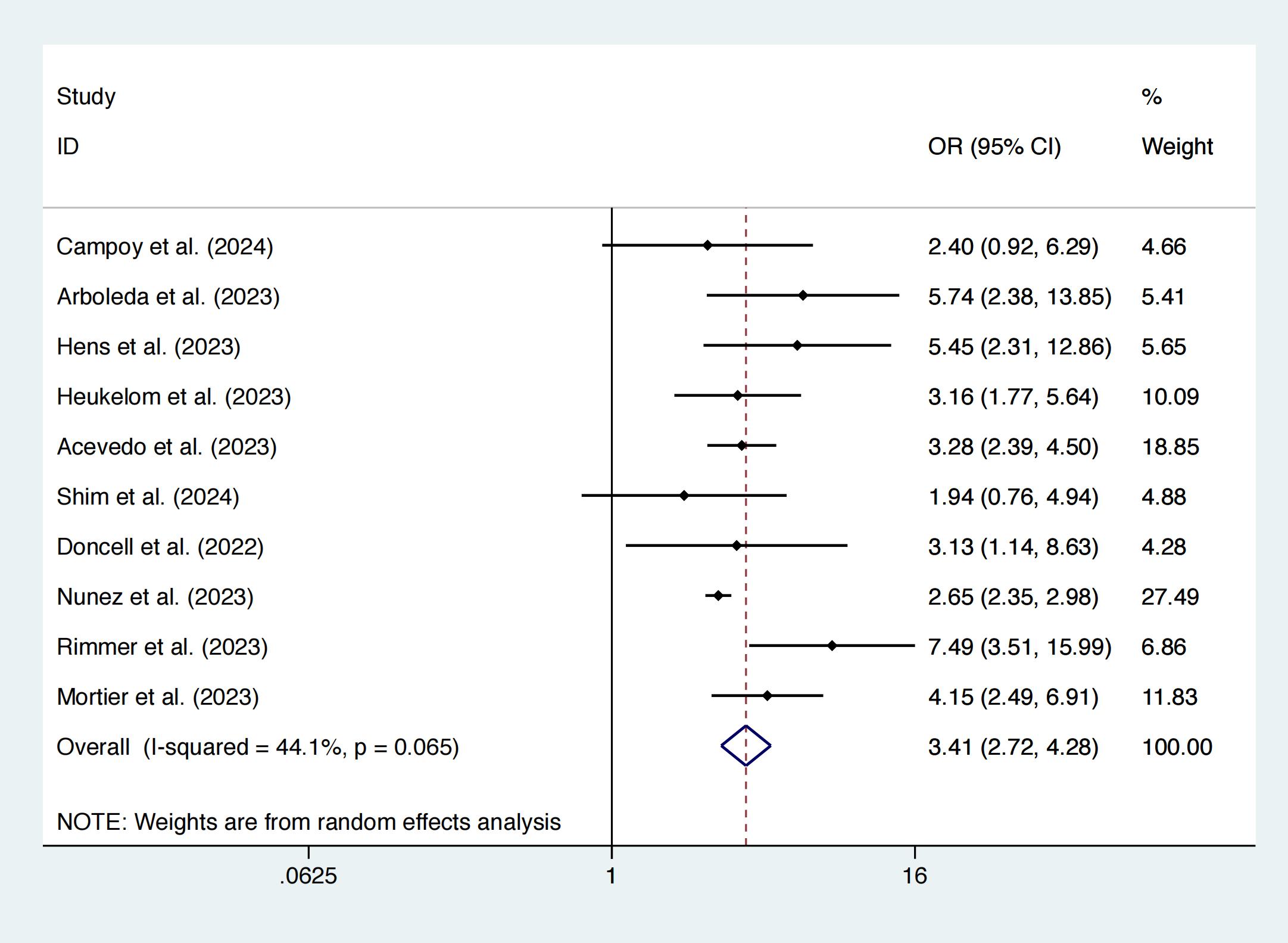


**Figure S45:** Forest plot of differences in symptoms between mpox patients and non-mpox patients: lymphadenopathy.


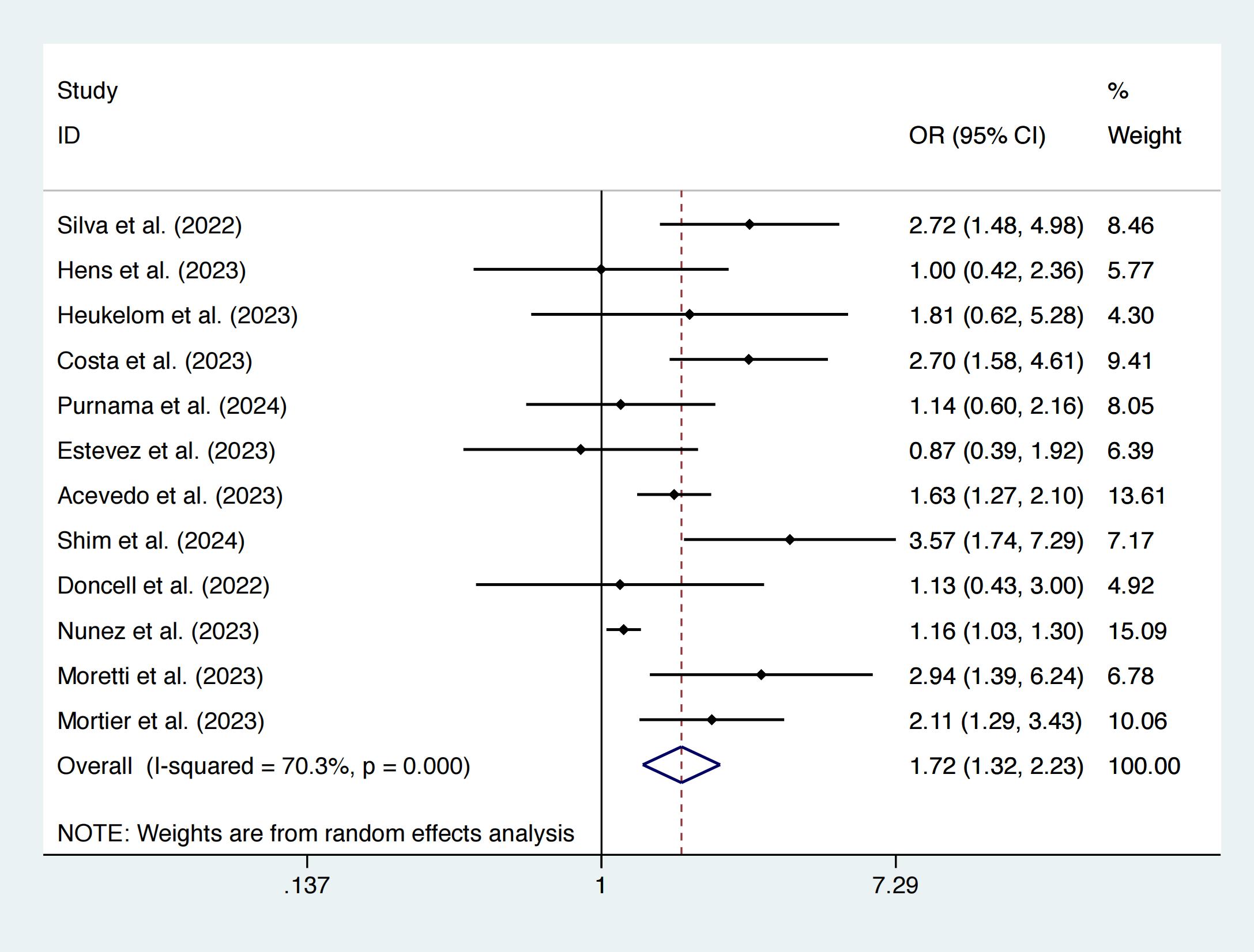


**Figure S46:** Forest plot of differences in symptoms between mpox patients and non-mpox patients:myalgia.


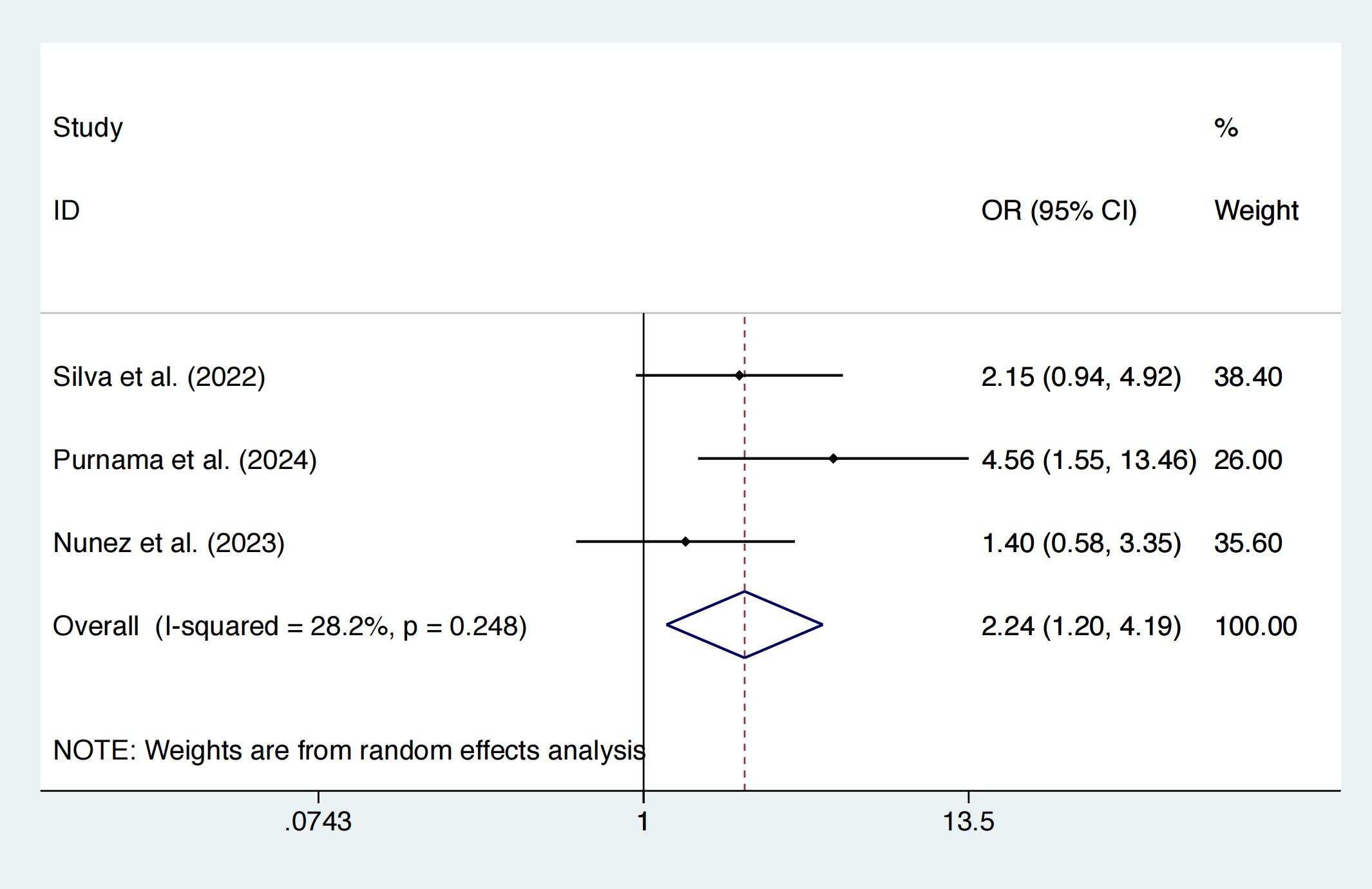


**Figure S47:** Forest plot of differences in symptoms between mpox patients and non-mpox patients: diarrhea.


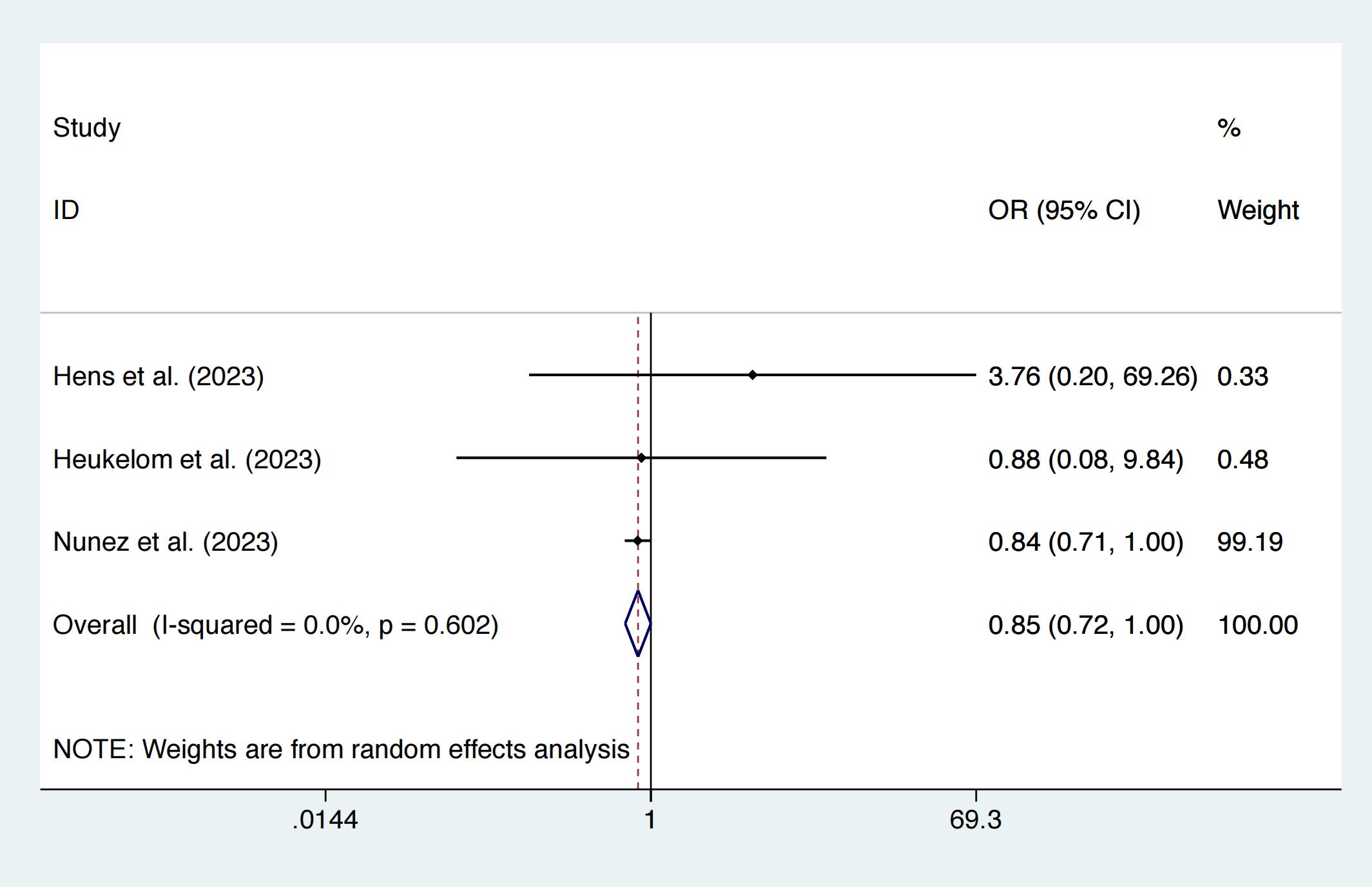


**Figure S48:** Forest plot of differences in symptoms between mpox patients and non-mpox patients: cough.


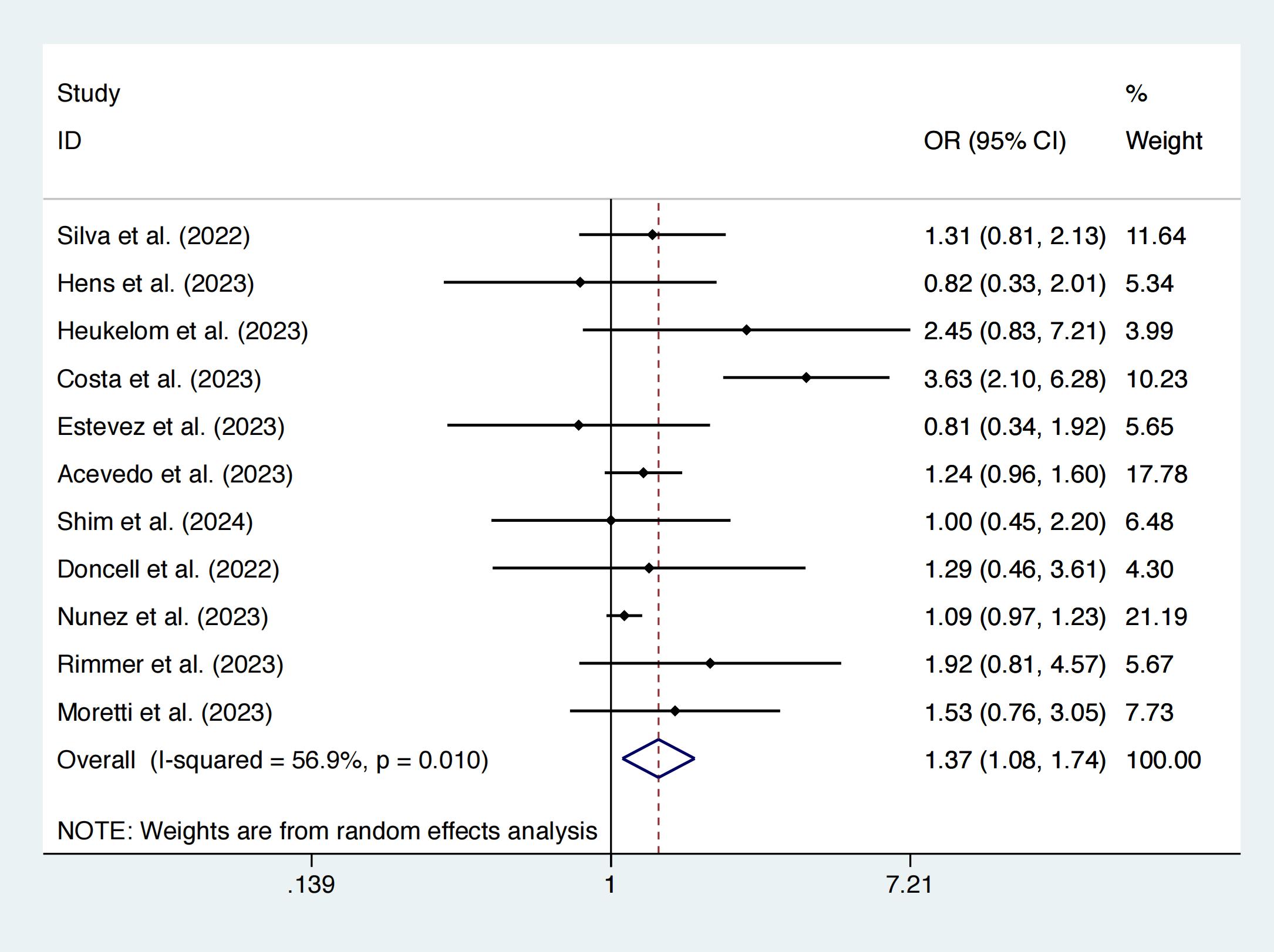


**Figure S49:** Forest plot of differences in symptoms between mpox patients and non-mpox patients: headache.


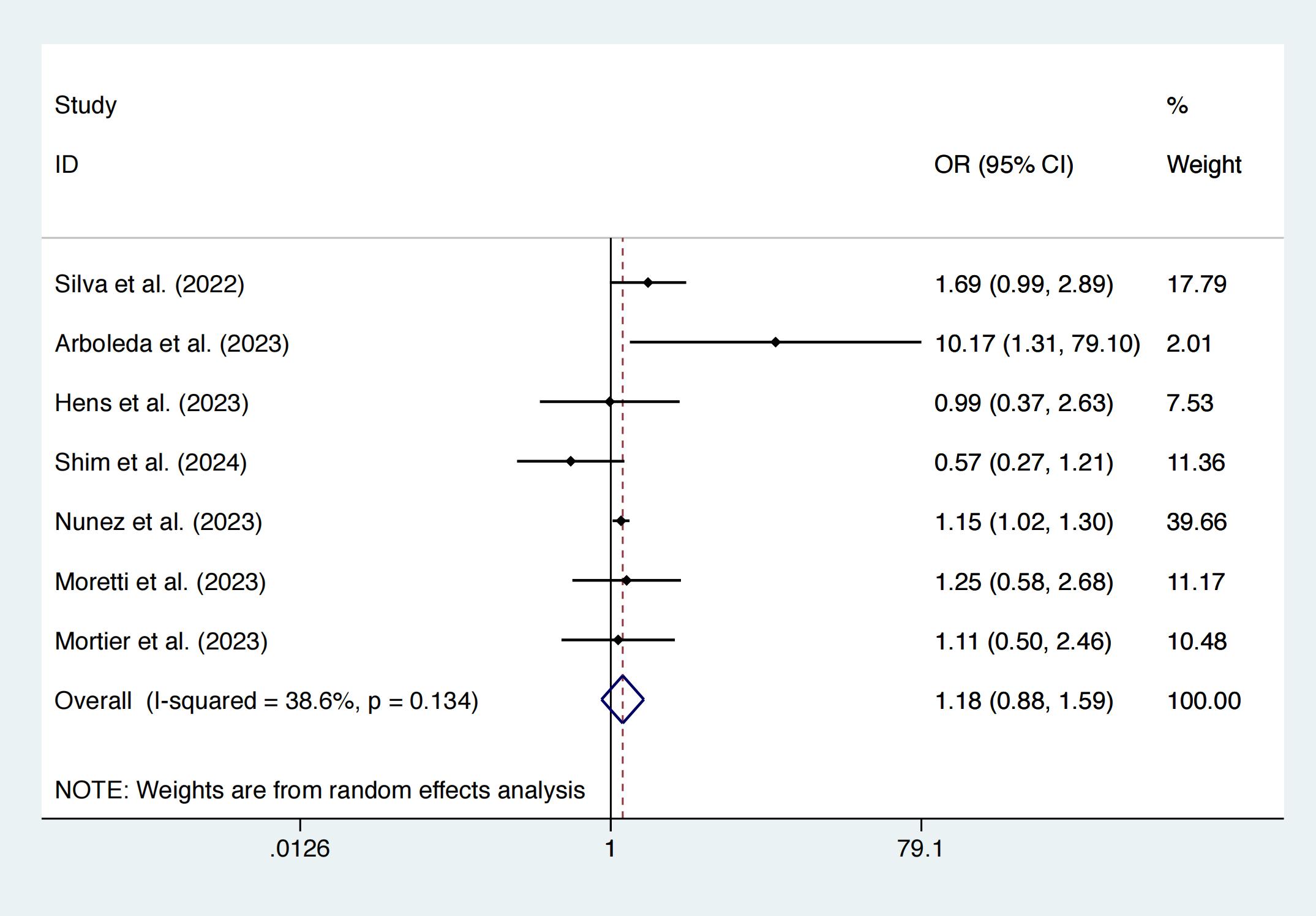


**Figure S50:** Forest plot of differences in symptoms between mpox patients and non-mpox patients: sore throat.


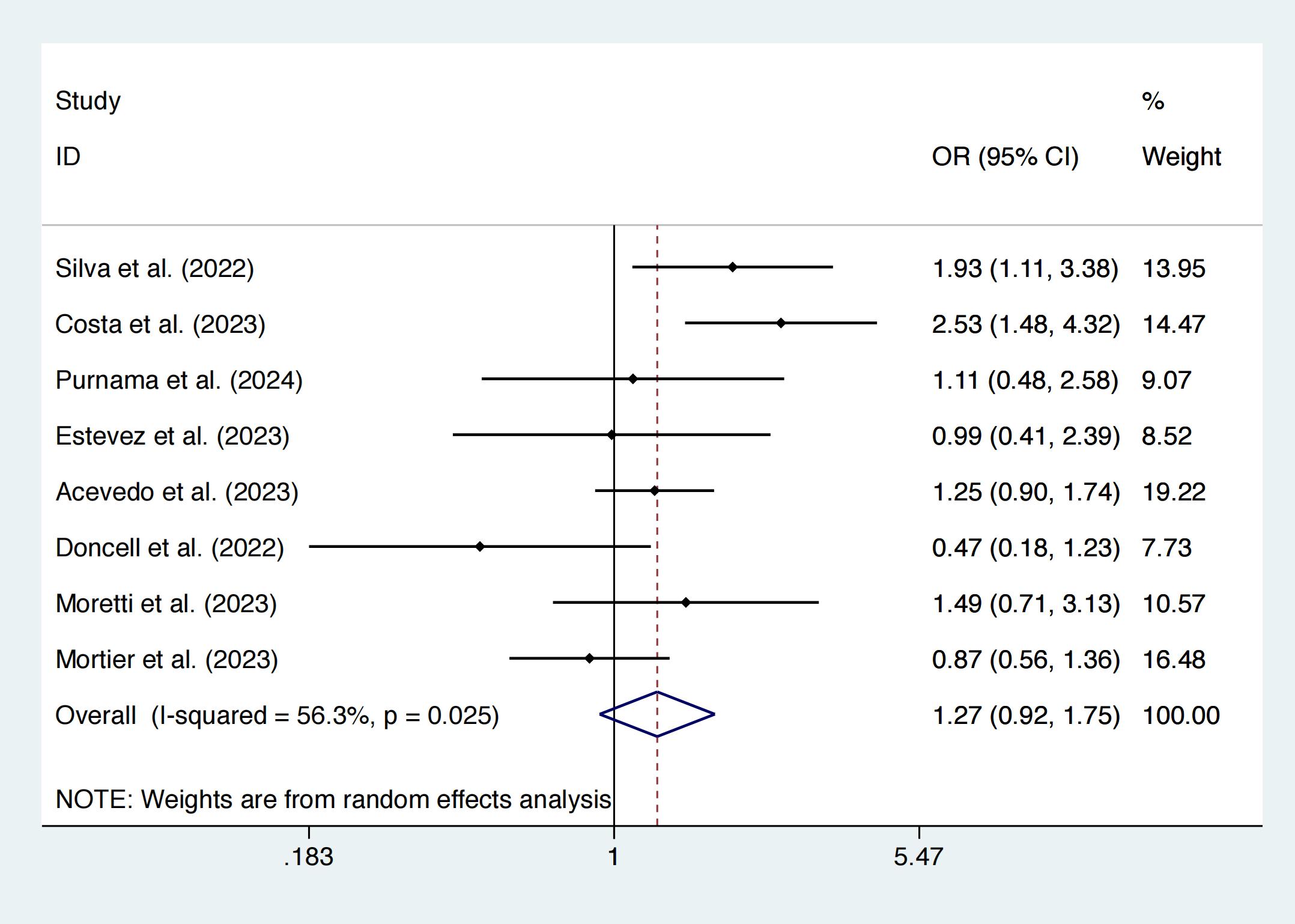


**Figure S51:** Forest plot of differences in symptoms between mpox patients and non-mpox patients: asthenia.


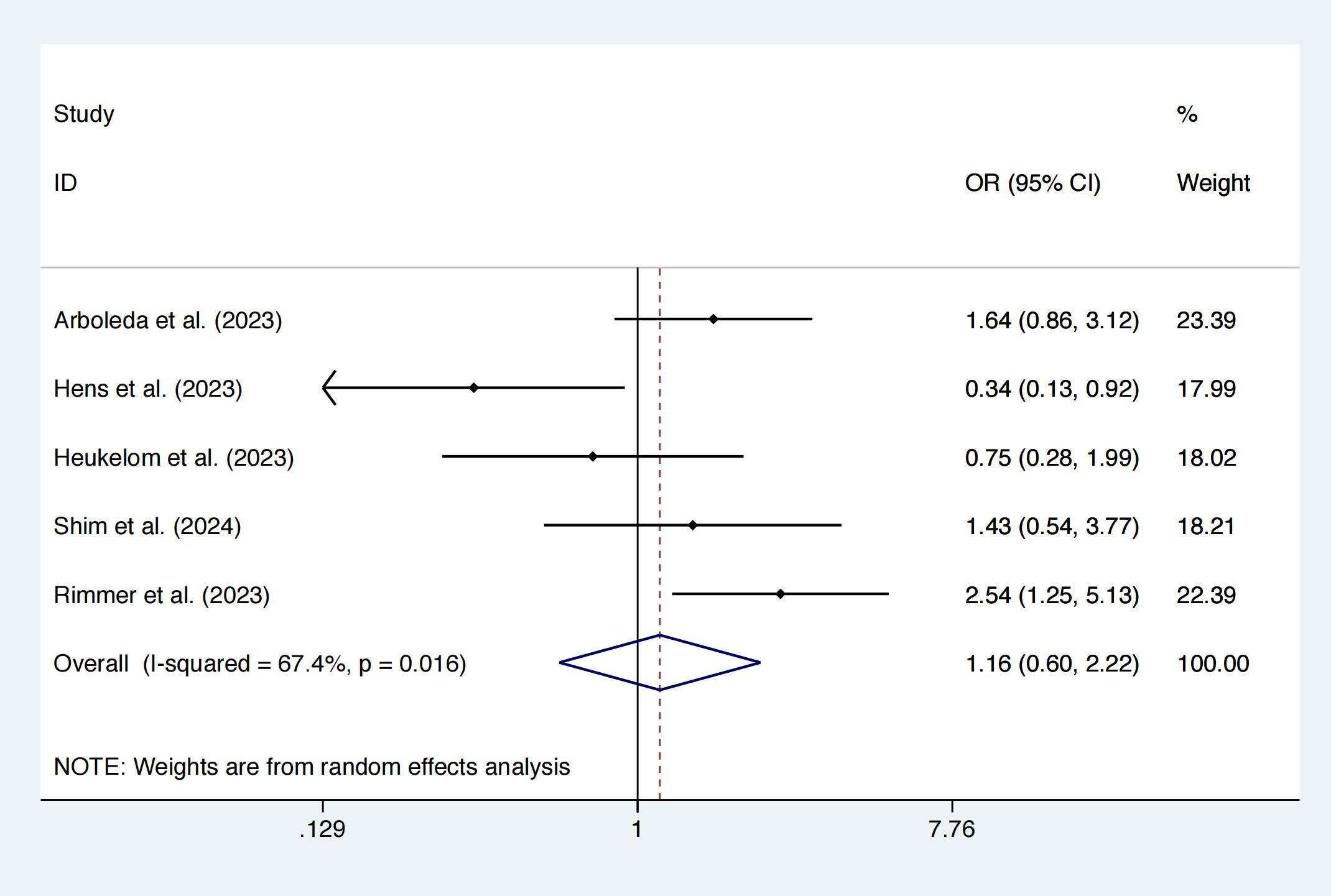


**Figure S52:** Forest plot of differences in symptoms between mpox patients and non-mpox patients: fatigue.


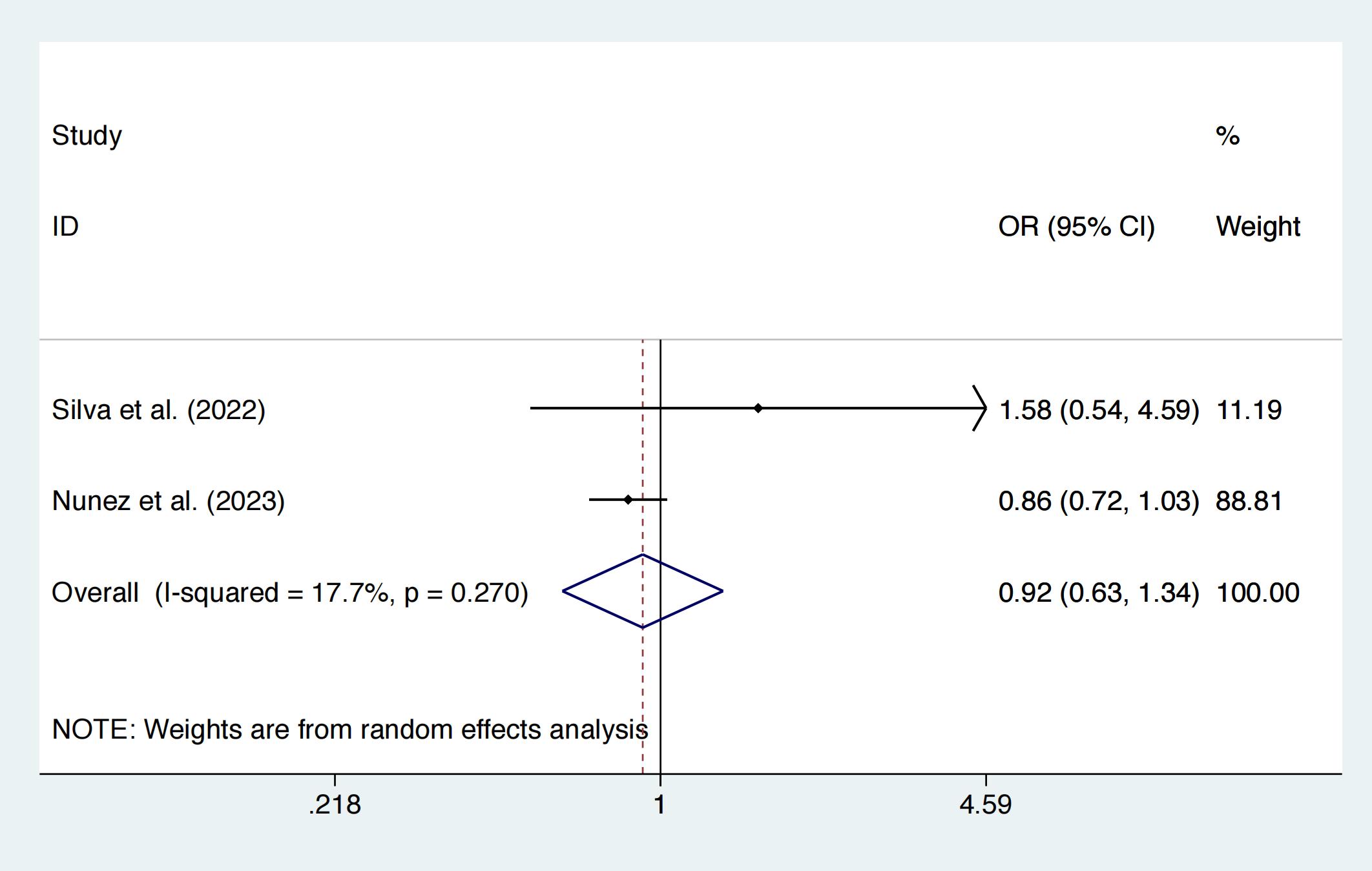


**Figure S53:** Forest plot of differences in symptoms between mpox patients and non-mpox patients: nausea.


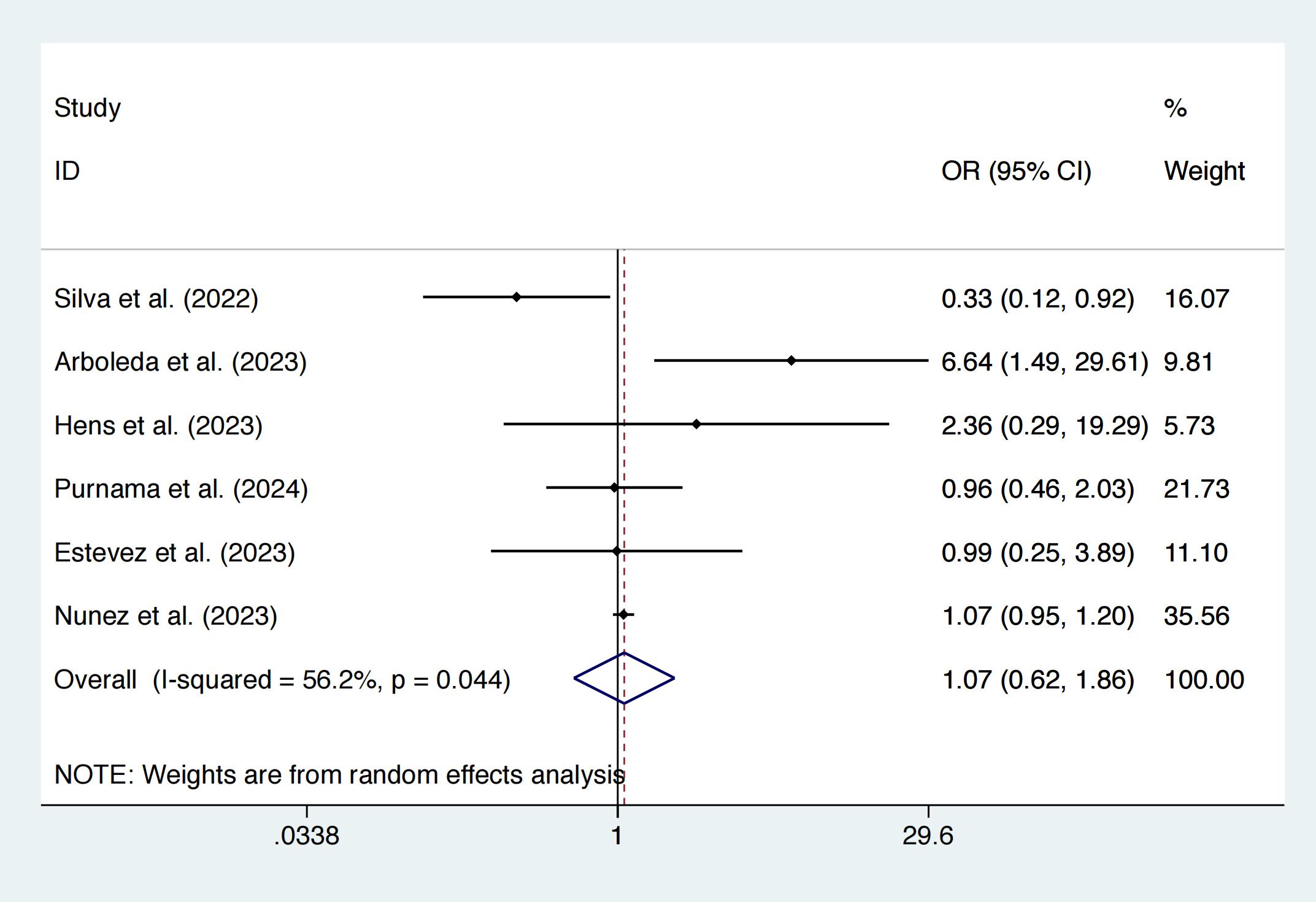


**Figure S54:** Forest plot of differences in symptoms between mpox patients and non-mpox patients: arthralgia.

**Figure S55:** Egger's publication bias plot of differences in age between mpox patients and non-mpox patients (p=0.042).

**Figure S56:** Egger's publication bias plot of differences in race between mpox patients and non-mpox patients: Black (p=0.339).

**Figure S57:** Egger's publication bias plot of differences in race between mpox patients and non-mpox patients: White (p=0.796).

**Figure S58:** Egger's publication bias plot of differences in recent exposure between mpox patients and non-mpox patients: contact with previous mpox patients (p=0.741).

**Figure S59:** Egger's publication bias plot of differences in recent exposure between mpox patients and non-mpox patients: oral sex (p=0.480).

**Figure S60:** Egger's publication bias plot of differences in recent exposure between mpox patients and non-mpox patients: travel (p=0.501).

**Figure S61:** Egger's publication bias plot of differences in transmission routes between mpox patients and non-mpox patients: health services (p=0.901).

**Figure S62:** Egger's publication bias plot of differences in sexually transmitted infections between mpox patients and non-mpox patients: human immunodeficiency virus (p=0.227).

**Figure S63:** Egger's publication bias plot of differences in sexually transmitted infections between mpox patients and non-mpox patients: pre-exposure prophylaxis (p=0.120).

**Figure S64:** Egger's publication bias plot of differences in sexually transmitted infections between mpox patients and non-mpox patients: syphilis (p=0.042).

**Figure S65:** Egger's publication bias plot of differences in sexually transmitted infections between mpox patients and non-mpox patients: gonorrhea (p=0.664).

**Figure S66:** Egger's publication bias plot of differences in sexually transmitted infections between mpox patients and non-mpox patients: hepatitis C virus (p=0.641).

**Figure S67:** Egger's publication bias plot of differences in sexually transmitted infections between mpox patients and non-mpox patients: chlamydia (p=0.843).

**Figure S68:** Egger's publication bias plot of differences in the locations of lesions between mpox patients and non-mpox patients: palms (p=0.996).

**Figure S69:** Egger's publication bias plot of differences in the locations of lesions between mpox patients and non-mpox patients: soles (p=0.703).

**Figure S70:** Egger's publication bias plot of differences in the locations of lesions between mpox patients and non-mpox patients: face (p=0.639).

**Figure S71:** Egger's publication bias plot of differences in the locations of lesions between mpox patients and non-mpox patients: head (p=0.444).

**Figure S72:** Egger's publication bias plot of differences in the locations of lesions between mpox patients and non-mpox patients: arms (p=0.110).

**Figure S73:** Egger's publication bias plot of differences in the locations of lesions between mpox patients and non-mpox patients: legs (p=0.116).

**Figure S74:** Egger's publication bias plot of differences in the locations of lesions between mpox patients and non-mpox patients: trunk (p=0.408).

**Figure S75:** Egger's publication bias plot of differences in the locations of lesions between mpox patients and non-mpox patients: oral cavity (p=0.339).

**Figure S76:** Egger's publication bias plot of differences in the locations of lesions between mpox patients and non-mpox patients: genitals (p=0.682).

**Figure S77:** Egger's publication bias plot of differences in the locations of lesions between mpox patients and non-mpox patients: perianal region (p=0.292).

**Figure S78:** Egger's publication bias plot of differences in the types of lesions between mpox patients and non-mpox patients: maculae (p=0.773).

**Figure S79:** Egger's publication bias plot of differences in the types of lesions between mpox patients and non-mpox patients: papulae (p=0.335).

**Figure S80:** Egger's publication bias plot of differences in the types of lesions between mpox patients and non-mpox patients: vesicles (p=0.096).

**Figure S81:** Egger's publication bias plot of differences in the types of lesions between mpox patients and non-mpox patients: pustules (p=0.220).

**Figure S82:** Egger's publication bias plot of differences in the types of lesions between mpox patients and non-mpox patients: scabs (p=0.710).

**Figure S83:** Egger's publication bias plot of differences in complications between mpox patients and non-mpox patients: proctitis (p=0.115).

**Figure S84:** Egger's publication bias plot of differences in complications between mpox patients and non-mpox patients: bacterial infection (p=0.238).

**Figure S85:** Egger's publication bias plot of differences in symptoms between mpox patients and non-mpox patients: systemic symptoms (p=0.622).

**Figure S86:** Egger's publication bias plot of differences in symptoms between mpox patients and non-mpox patients: cough (p=0.472).

**Figure S87:** Egger's publication bias plot of differences in symptoms between mpox patients and non-mpox patients: fever (p=0.003).

**Figure S88:** Egger's publication bias plot of differences in symptoms between mpox patients and non-mpox patients: lymphadenopathy (p=0.050).

**Figure S89:** Egger's publication bias plot of differences in symptoms between mpox patients and non-mpox patients: headache (p=0.188).

**Figure S90:** Egger's publication bias plot of differences in symptoms between mpox patients and non-mpox patients: sore throat (p=0.671).

**Figure S91:** Egger's publication bias plot of differences in symptoms between mpox patients and non-mpox patients: asthenia (p=0.696).

**Figure S92:** Egger's publication bias plot of differences in symptoms between mpox patients and non-mpox patients: fatigue (p=0.153).

**Figure S93:** Egger's publication bias plot of differences in symptoms between mpox patients and non-mpox patients:myalgia (p=0.053).

**Figure S94:** Egger's publication bias plot of differences in symptoms between mpox patients and non-mpox patients: diarrhea (p=0.407).

**Figure S95:** Egger's publication bias plot of differences in symptoms between mpox patients and non-mpox patients: arthralgia (p=0.856).

**Figure S96:** Egger's publication bias plot of differences in previous mpox vaccination between mpox patients and non-mpox patients (p=0.017).
